# Novel Substituted Purine Isosteres: Synthesis, Structure-Activity Relationships and Cytotoxic Activity Evaluation

**DOI:** 10.3390/molecules27010247

**Published:** 2021-12-31

**Authors:** Spyridon Dimitrakis, Efthymios-Spyridon Gavriil, Athanasios Pousias, Nikolaos Lougiakis, Panagiotis Marakos, Nicole Pouli, Katerina Gioti, Roxane Tenta

**Affiliations:** 1 Department of Pharmacy, Division of Pharmaceutical Chemistry, National and Kapodistrian University of Athens, Panepistimiopolis, 15771 Zografou, Greece; sdimitrakis@pharm.uoa.gr (S.D.); efthymios-spyridon.gavriil@cruk.manchester.ac.uk (E.-S.G.); thanpous@pharm.uoa.gr (A.P.); nlougiak@pharm.uoa.gr (N.L.); pouli@pharm.uoa.gr (N.P.); 2 Department of Nutrition & Dietetics, School of Health Sciences and Education, Harokopio University, 17671 Athens, Greece; catherine_geo@yahoo.com (K.G.); rtenta@hua.gr (R.T.)

**Keywords:** purine isosteres, pyrrolopyridine, pyrrolopyrimidine, pyrazolopyrimidine, synthesis, cytotoxic activity, cell cycle perturbation, apoptosis

## Abstract

A number of pyrrolo[2,3-*c*]pyridines, pyrrolo[3,2-*d*]pyrimidines and pyrazolo[4,3-*d*]pyrimidines were designed and synthesized as antiproliferative agents. The target compounds possessed selected substituents in analogous positions on the central scaffold that allowed the extraction of interesting SARs. The cytotoxic activity of the new derivatives was evaluated against prostatic (PC-3) and colon (HCT116) cell lines, and the most potent analogues showed IC_50_ values in the nM to low µM range, while they were found to be non-toxic against normal human fibroblasts (WI-38). Flow cytometric analysis of DNA content revealed that the most promising derivative **14b** caused a statistically significant accumulation of PC-3 cells at G_2_/M phase and induced apoptosis in PC-3 cells.

## 1. Introduction

Azaindole and related nitrogen-containing fused heterocycles, presenting structural analogy to adenine base of DNA, are important building blocks of bioactive compounds, and they have, therefore, been extensively investigated in the field of medicinal chemistry [1,2,3]. Among this structurally diverse class, many pyrazolopyridine and pyrrolopyrimidine derivatives were found to display potent in vitro and in vivo activity, as receptor modifiers or enzyme inhibitors [4,5], possessing interesting antiproliferative [6,7,8], antibacterial [9,10], antiviral [11,12], antimalarial [13], antileishmanial [14], antidiabetic [15], trypanocidal [16] and neuroprotective properties [17]. The exact heterocyclic isomer, depending on the structural arrangement of nitrogen atoms in the central purine-like ring system, along with the introduction of selected substituents, is important for the interaction of each compound with specific cellular substrates. The classical bioisosteric replacement of a CH group with a N atom (vide infra) in heteroaromatic rings can potentially affect a number of the physicochemical parameters of the central scaffold, as well as its intra- and intermolecular orbital, steric, electrostatic and hydrophobic interactions, which can translate into improved pharmacological profiles. This minor structural modification is usually challenging from a synthetic point of view, but it provides an excellent opportunity for lead optimization. [18] Although the antitumor activity of these compounds can be attributed to a broad range of molecular targets and several mechanisms, the more interesting targets and mechanisms are certainly related to the inhibition of receptor or cytoplasmic protein kinases, which are linked to signal transduction networks associated with carcinogenesis, progression and metastasis of a variety of aggressive human cancers [19]. Purine isosteres usually compete successfully with ATP for interaction with the hinge region of the binding pocket of these enzymes and, therefore, are estimated to be non-selective kinase inhibitors. However, a reasonable degree of selectivity can be achieved as a consequence of the introduction of a variety of substituents, mainly on the respective 2-, 6-and 9-positions of the purine nucleus. For example, the pyrrolopyridine derivative vemurafenib is a mutant specific kinase inhibitor currently used for treatment of metastatic BRAFV600E melanoma [20]. Extensive lead optimization studies are essential in each case to enhance interactions with binding pockets, neighboring the hinge region, in order to improve potency and target selectivity. Nevertheless, the great number of protein kinases, the complexity of cellular signal transduction pathways and the possibility of interactions with additional cellular substrates mean that experimental studies aimed at the clarification of the precise pharmacological function of individual derivatives are challenging.

On the other hand, there is an increasing need for the development of novel, safer and tolerable chemotherapeutic agents for more effective cancer treatment. The identification and targeting of the molecular drivers of cancer remain a major scientific subject and contribute towards the discovery of new compounds, able to inhibit uncontrolled cancer cell proliferation, with low toxicity to normal cells and tissues. These targeted therapies are considered a promising approach for the treatment of human malignancies and have already been proven effective in the clinic, providing impressive—though transitory—responses to patients with advanced cancers. They are well-tolerated drugs, characterized by few off-target effects, low toxicity profiles and fairly broad therapeutic windows. However, the development of therapeutic strategies to improve the long-term effectiveness and limit the emerging resistance in targeted therapies remain a vital challenge in this field. [21].

The starting point for the present study was an attractive pyrazolopyridine hit, namely 3-phenyl-7-(3,4,5-trimethoxyphenyl)aminopyrazolo[3,4-*c*]pyridine, previously identified by our group [22]. This compound was endowed with strong cytotoxic properties against a variety of cancer cell lines, with IC_50_ values in the medium to high nM range. In the course of our involvement in the discovery of new purine analogues with antiproliferative activity [23,24,25], we considered it interesting to modify the main scaffold of the above-mentioned derivative and insert suitable substituents in order to gain insight into the SARs of this class of compounds. More precisely, we have replaced the pyrazolopyridine central scaffold by three purine-like heterocycles, namely pyrrolo[2,3-*c*]pyridine, pyrrolo[3,2-*d*]pyrimidine and pyrazolo[4,3-*d*]pyrimidine. Besides the 7-(3,4,5-trimethoxyphenyl)- substituent of the lead compound, we have also introduced a phenylamino- and a 4-methylpiperazin-1-yl substituent in the corresponding position of the above-mentioned scaffolds. We preserved the lead compound’s 3-phenyl group, and we also prepared the corresponding 3-(3-fluorophenyl)- analogues. We, thus, present herein the synthesis of these series of derivatives and the evaluation of their biological activity.

## 2. Results and Discussion

### 2.1. Chemistry

**Scheme 1:**
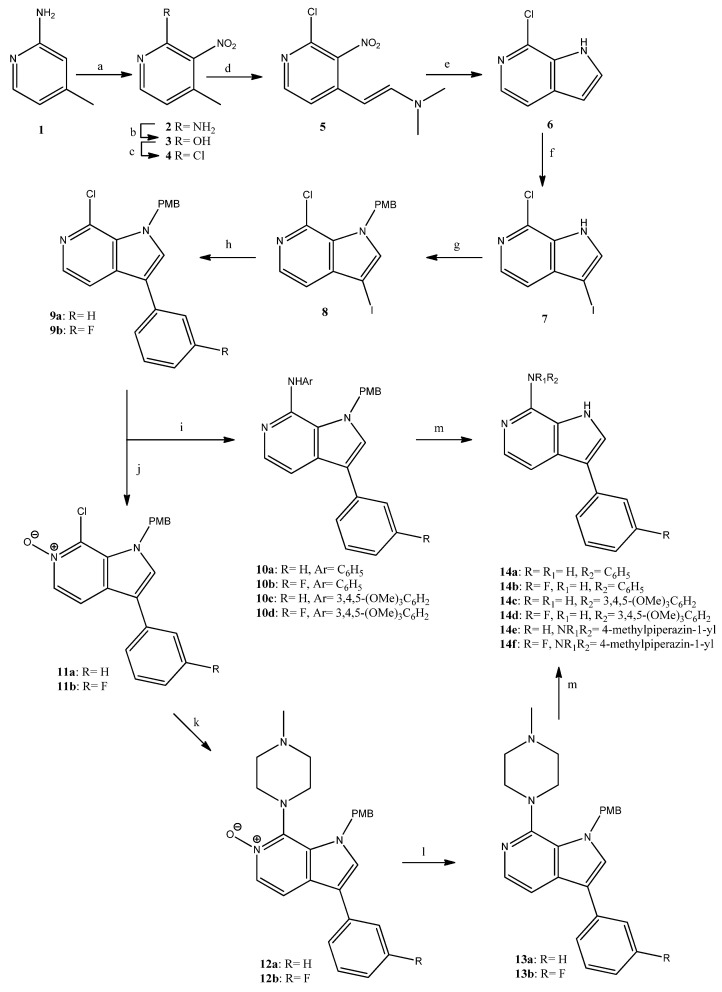
Reagents and conditions. a: HNO_3_, H_2_SO_4_, 60 °C, 4 h, 44%; b: NaNO_2_, H_2_SO_4_, rt, 2 h, 90%; c: POCl_3_, reflux, 4 h, 94%; d: DMF-DMA, DMF, 120 °C, 2 h, 80%; e: Fe, AcOH, AcONa, EtOH/H_2_O, reflux, 2 h, 82%; f: NIS, MeOH, rt, 1 h, 99%; g: NaH, 4-methoxybenzylchloride (PMB-Cl), DMF, rt, 90 min, 99%; h: phenylboronic acid or 3-fluorophenylboronic acid, K_2_CO_3_, tetrakis(triphenylphosphine)palladium, toluene/EtOH, reflux, 3 h, 88–98%; i: aniline or 3,4,5-trimethoxyaniline, Pd_2_(dba)_3_, BINAP, *t*-BuOK, toluene, reflux, 3.5 h, 60–91%; j: m-chloroperoxybenzoic acid, CH_2_Cl_2_, rt, 72 h, 88%; k: 1-methylpiperazine, EtOH, autoclave, 120 °C, 24 h, 50%; l: PCl_3_, CHCl_3_, rt, 20 h, 72–99%; m: CF_3_CO_2_H, 60 °C, 20 h, 91–99%.

For the preparation of the series of pyrrolopyridines, we used the intermediate chlorides **9a,b**, which were synthesized as depicted in Scheme 1. 2-Aminopicoline (**1**) was successively nitrated, diazotized and chlorinated to provide the nitropyridine **4** [26]. The reaction of **4** with DMF-dimethylacetal provided the enamine **5** that upon reduction was cyclized to result in the 7-chloropyrrolopyridine **6** [27] in 25% overall yield. The latter was iodinated, the pyrrole nitrogen was protected upon treatment with 4-methoxybenzylchloride (PMB-Cl) and the resulting iodide **8** underwent a Suzuki-type coupling with suitable boronic acids to give the chlorides **9a,b**. All these reactions proceeded in high yields, and compounds were rationally characterized via ^1^H-NMR data. It is of interest to notice the characteristic chemical shift of the iodine bearing carbon (C-3) of compounds **7** and **8**, which resonated at 56 ppm in the corresponding ^13^C-NMR spectra. The chlorides **9a,b** were then treated with aniline, or 3,4,5-trimethoxyaniline in the presence of potassium *tert*-butoxide, tris(dibenzylideneacetone)dipalladium(0) [Pd_2_(dba)_3_] and 2,2′-bis(diphenylphosphino)-1,1′-dinapthalene (BINAP), to result in the amines **10a–d** in good yields (60–91%). In parallel, since the nucleophilic substitution of the 7-chloro group of compounds **9** by alkylamines upon heating in DMF or DMSO was not successful, **9a,b** were treated with m-chloroperoxybenzoic acid and were converted in good yield (88%) to the corresponding *N*-oxides **11a,b**. These derivatives underwent nucleophilic substitution by *N*-methylpiperazine to provide the aminosubstituted compounds **12a,b** in moderate yield (50%), which upon reduction gave the intermediates **13a,b**. An interesting and indicative feature in the ^13^C-NMR spectral data of the *N*-oxides **11a,b** and **12a,b** was the chemical shift of C-5, which appears upfield, at 133–134 ppm, when compared to the corresponding peak of compounds **9a,b** and **13a,b** (C-5 resonance in this case is 137–138 ppm); this was obviously due to the presence of the neighboring *N*-oxide. Finally, the *N*1-substituted derivatives **10a–d** and **13a,b** were efficiently deprotected to result in the target pyrrolopyridines **14a–f** in good to quantitative yields. In the ^1^H-NMR spectra of compounds **14a–f**, the pyrrole H was detectable as a broad singlet at 10.5–11.8 ppm, whereas the NH of the 7-substituent of **14a–d** was detected upfield at 8.6–9.4 ppm.

**Scheme 2:**
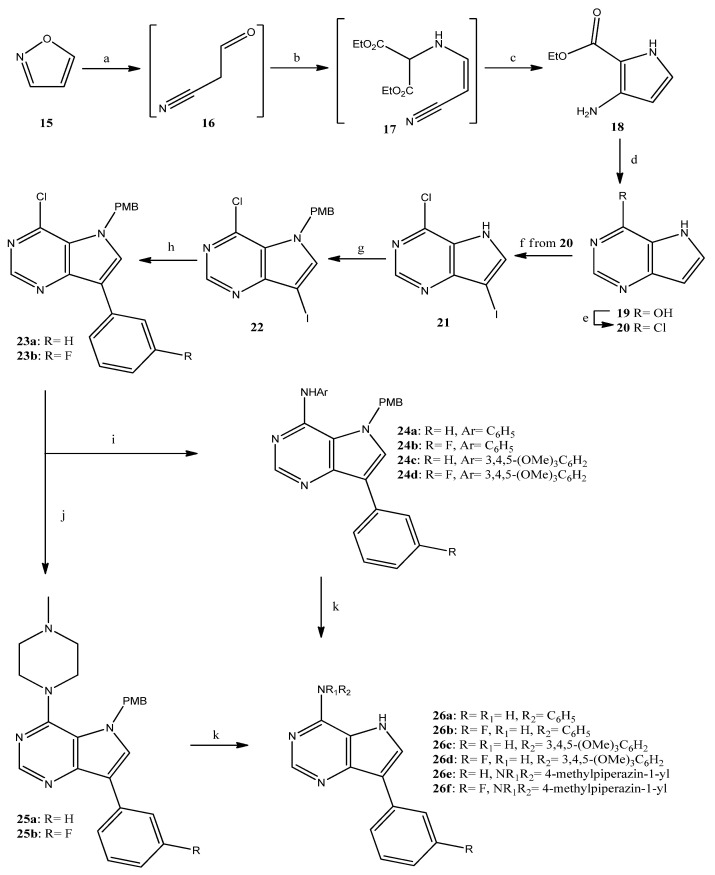
Reagents and conditions. a: EtONa, EtOH, rt, 30 min; b: ethyl aminomalonate hydrochloride, AcOH, AcONa, rt, 48 h; c: EtONa, EtOH, rt, 5 days, 37% for 3 steps; d: formamidine acetate, EtOH, reflux, 20 h, 82%; e: POCl_3_, reflux, 90 min, 88%; f: NIS, MeOH, rt, 1 h, 76%; g: NaH, 4-methoxybenzylchloride (PMB-Cl), DMF, rt, 90 min, 99%; h: phenylboronic acid or 3-fluorophenylboronic acid, K_2_CO_3_, tetrakis(triphenylphosphine)palladium, toluene/EtOH, reflux, 3 h, 90–94%; i: aniline or 3,4,5-trimethoxyaniline, Pd_2_(dba)_3_, BINAP, *t*-BuOK, toluene, reflux, 5 h, 60–87%; j: 1-methylpiperazine, DMSO, 120 °C, 20 h, 97–99%; k: CF_3_CO_2_H, 60 °C, 20 h, 91–99%.

The pyrrolopyrimidine analogues were prepared according to the reaction sequence presented in Scheme 2 from isoxazole (**15**), which—following a previously reported procedure—was converted to the pyrrolecarboxylate **18** [28]. The latter was, in turn, ring-closed; the resulting pyrimidinone **19** [29] was converted to the chloride **20** [30], which was successively iodinated to give **21** [31]. The iodide **21** was then protected to provide almost quantitatively **22**, which was introduced to a Suzuki-type coupling, resulting in high yields of the 3-arylsubstituted chlorides **23a,b**. The above-mentioned chlorides were used for the synthesis of the target pyrrolopyrimidines **26a**–**f**, obtained in very good overall yields, following an analogous procedure to that reported for the corresponding pyrrolopyridines. A common pattern was obvious at the ^1^H-NMR spectra of **26a**–**f**, concerning the pyrrole NH that appeared as a broad singlet at 10.7–11.1 ppm and the NH of the 4-substituent of compounds **26a**–**d** (8.5–8.7 ppm), as well as the characteristic chemical shifts of H-2 (8.4–8.5 ppm) and H-6 (7.9–8.1 ppm).

**Scheme 3:**
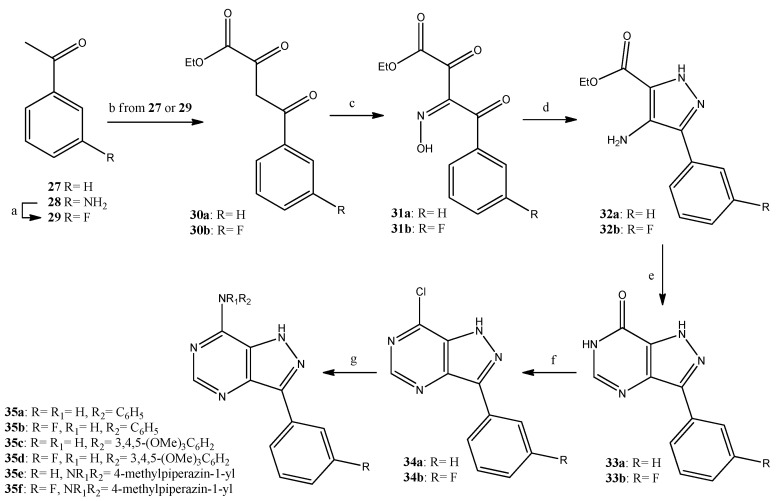
Reagents and conditions. a: BF_3_^.^(Et_2_O)_2_, isoamyl nitrite, 1,2-dichlorobenzene, 90 °C, 45 min, 54%; b: NaH, diethyl oxalate, toluene, 50 °C, 1 h, 99%; c: NaNO_2_, AcOH, 10 °C, 1 h, 85%; d: hydrazine hydrate, EtOH, 45 °C, 2 h, 40%; e: formamidine acetate, BuOH, reflux, 20 h, 61%; f: POCl_3_, PCl_5_, reflux, 3 h, 80%; g: aniline or 3,4,5-trimethoxyaniline or 1-methylpiperazine, EtOH, reflux, 2 h, 57–80%.

Finally, the pyrazolopyrimidine analogues were synthesized, as shown in Scheme 3, starting from the acetophenones **27** (commercially available) or **29** [32]. The acetophenones were subjected to side chain elongation upon reaction with ethyl oxalate, and the resulting dioxobutyrates **30a** [33] and **30b** [34] were converted in good yields (85%) to the corresponding α-(hydroxyimino)benzopyruvates **31a** [35] and **31b,** respectively. Compounds **31a,b** were treated with hydrazine hydrate and were ring-closed with the concomitant reduction of the oxime group to result in the aminopyrazole-carboxylates **32a** [36] and **32b**, in moderate yields (40%). The reaction of the carboxylates **32a,b** with formamidine gave the pyrazolopyrimidinones **33a** [37] and **33b**, which—through the corresponding chlorides **34a,b** and the displacement of the chlorine by suitable amines—resulted in the target pyrazolopyrimidines **35a–f** in good overall yields (over 40% for three steps). All new compounds were unambiguously identified using NMR and mass spectrometry data.

### 2.2. Biological Evaluation

The cytotoxic activity of the new compounds was tested against two cancer cell lines, prostatic (PC-3) and colon (HCT116) cell lines (Table 1), while the most potent derivatives were further tested against normal human fibroblasts (WI-38) (Table 2).

Concerning the *N*-(p-methoxybenzyl) substituted pyrrolopyridines **10a**–**d** and **13a,b**, only a few of them possessed moderate antiproliferative activity, namely the 7-phenylamino derivative **10a**, together with both the 7-(*N*-methylpiperazine) substituted derivatives **13a,b**, with IC_50_ values in the range of 2.4–9.3 µM. On the other hand, the deprotection of the above-mentioned compounds provided derivatives **14a**–**f**, which were endowed with very potent cytotoxic properties, with IC_50_ values in the nM to low µM range against both cell lines tested. The most potent compound of this group is the 3-(3-fluorophenyl) analogue **14b**, possessing IC_50_ values of 55nM and 90 nM against the prostate and colon cell lines, respectively.

In the pyrrolopyrimidine series, the *N*-(p-methoxybenzyl) substituted analogues **44a**–**d** and **25a,b** are, in analogy to the previous series, devoid of activity, with the sole exception of **24c,** which appeared to be cytotoxic against PC-3 cells (IC_50_: 2.25 µM). Concerning the corresponding deprotected analogues, the 7-(3-fluorophenyl)-4-phenylamino derivative **26b** is the most potent compound of this group, followed by the corresponding 7-phenyl-substituted compound **26a**. The 4-(3,4,5-trimethoxyphenyl)amino-substituted derivatives **26c,d** had moderate activity, while a complete loss of activity was observed in the corresponding 4-(*N*-methylpiperazine)-substituted analogues.

Finally, in the case of pyrazolopyrimidines, the 3-(3-fluorophenyl)-7-phenylamino derivative **35b** possessed very interesting cytotoxicity, whereas among the remaining compounds, only **35a** and **35c** showed moderate cytotoxicity, and again the *N*-methylpiperazine substituted analogues were inactive.

Physicochemical characteristics of the thirty compounds tested were calculated using SwissADME platform [38] and are summarized in Table S1. They were further entered as inputs along with the IC_50_s in the SIMCA software [39]. A multivariate analysis showed that there is a very weak correlation (R2 < 0.25) between activity and molar refractivity, while there was no correlation between activity and logP.

As a general remark, it could be stated that the cytotoxic activity is enhanced in the absence of substituents on the pyrrole or pyrazole nitrogen of the studied compounds and that the pyrrolopyridines possess the most interesting profile, since all six derivatives (**14a–f**) are potent against both cell lines tested. As already mentioned, **14b** is the most active derivative, and at the same time, the analogously substituted pyrrolopyrimidine **26b** and pyrazolopyrimidine **35b** are also endowed with strong cytotoxic properties. It is of interest to notice that the prostatic cell line appears the most sensitive to the compounds.

**Figure 1:**
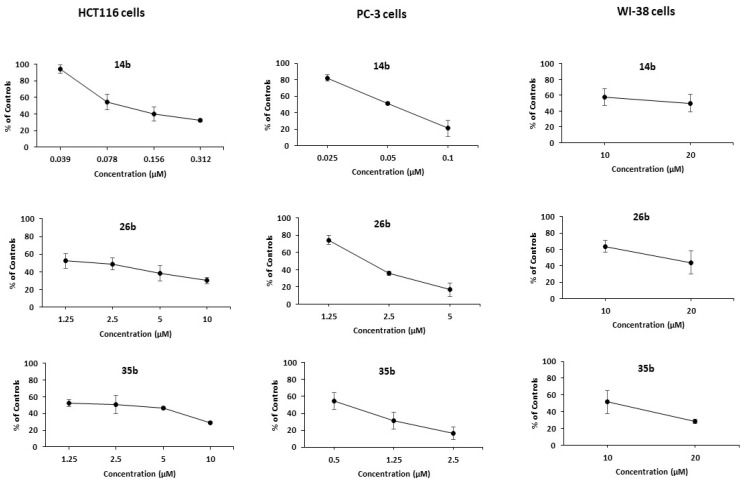
Dose-response curves for the administration of **14b**, **26b**, **35b** on HCT116, PC-3 and WI-38 cells, as assessed by the MTT assay. Results are expressed as a percentage of controls. Statistical analysis was performed in triplicate determination at *p* < 0.05.

The cytotoxicity of the most potent derivatives, i.e., the majority of the pyrrolopyridines **14**, together with **26b** and **35b**, was then examined towards normal human fibroblasts (WI-38); the results are presented in Figure 1 and Table 2.

Among them, **14a** and **14c**–**e** appear to be rather cytotoxic to normal cells. By contrast, it is important to note that the 7-phenylamino substituted compound **14b** that bears a 3-(3-fluorophenyl)-group, while being highly cytotoxic against both cancer cell lines, appears practically non-toxic against the normal cell line, presenting a selectivity index higher than 200. The derivatives **26b** and **35b** that possess the same substitution pattern in the central scaffold retain potent activity against the cancer cell lines and appear non-cytotoxic to the normal cell line as well, albeit to a lesser extent than **14b**.

**Figure 2:**
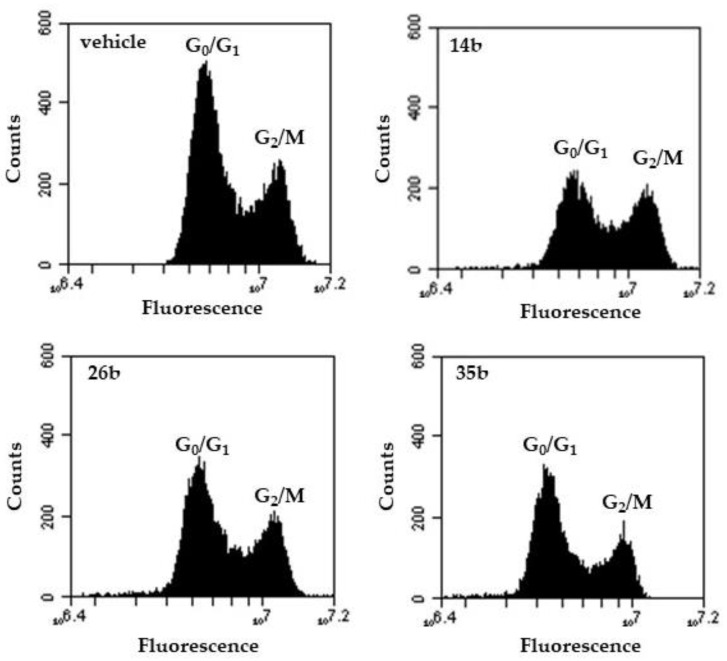
Flow cytometric graphs of DNA content. Indicative DNA content histograms of PC-3 cells treated for 72 h with **14b**, **26b** and **35b**, in comparison to control cells (vehicle). Quantification of cell cycle phase distribution was based on staining by the DNA binding dye propidium iodide.

Cell-cycle perturbations induced after the incubation of exponentially growing PC-3 cells with compounds **14b, 26b** and **35b** for 72 h are given in Figure 2 and Table 3. Compound **14b** caused a statistically significant accumulation of PC-3 cells at G_2_/M phase, significantly reducing, in parallel, the percentage of cells at G_0_/G_1_ phase (the accumulation of cells at S phase was marginally non-significant).

**Figure 3:**
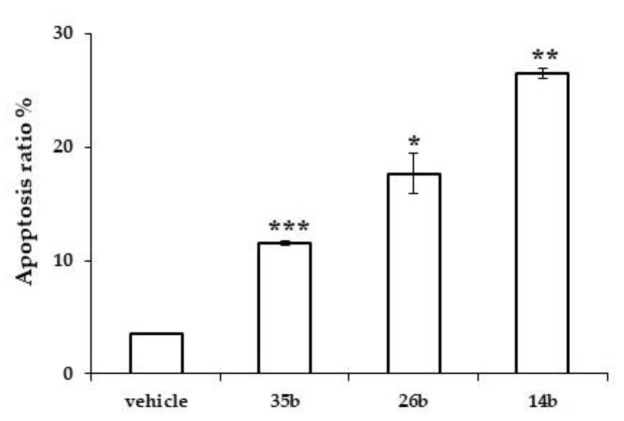
Induction of apoptosis by the test compounds. Percentage of apoptotic nuclei in PC-3 cells treated for 72 h with the indicated compounds or with vehicle. Bars represent the mean of two independent experiments (error bars: SD); * indicates *p* < 0.05, ** indicates *p* < 0.005, *** indicates *p* < 0.001 (Student’s *t*-test).

Doxorubicin, which was used as a positive control at a concentration of 25 nM, showed the expected G2/M phase blockade (58%) and the induction of apoptosis (28%), as previously reported [40]. Furthermore, **14b**, **26b** and **35b** induced apoptosis in PC-3 cells after 72 h of treatment, as estimated by AnnexinV-7AAD staining. In particular, compound **14b** induced the appearance of 26.5% (±0.47) apoptotic nuclei, **26b** induced the appearance of 17.7% (±1.8) apoptotic nuclei and **35b** induced the appearance of 11.5% (±0.19) apoptotic nuclei, compared to 3.5% (±0.05) induced by the vehicle (Figure 3). These data give an indication that the mechanism of action of compound **14b** may include blocking of the cell cycle at the G_2_/M phase and inducing apoptosis.

## 3. Materials and Methods

### 3.1. General Information

The reagents and solvents were purchased from Sigma-Aldrich Chemical Co. (Darmstadt, Germany) or Fluorochem (Derbyshire’s Peak District, UK). Reagents were used without further purification. Concerning the dry solvents, methanol and dimethylformamide were dried over 3A and 4A molecular sieves, respectively; toluene was pre-dried using CaH_2_ and then placed over sodium. Hydrazine is considered a suspect carcinogen in humans, may cause serious damage and should be handled carefully. Melting points were determined on a Büchi apparatus (Flawil, Switzerland) and are uncorrected. ^1^HNMR spectra and 2D spectra were recorded on a Bruker Avance III 600 or a Bruker Avance DRX 400 instrument (Bruker BioSpin, Baden-Württemberg, Germany), whereas ^13^CNMR spectra were recorded on a Bruker Avance III 600 spectrometer in deuterated solvents and were referenced to TMS (*δ* scale). The signals of ^1^H and ^13^C spectra were unambiguously assigned by using 2D NMR techniques: ^1^H^1^H COSY, NOESY, HMQC and HMBC. Mass spectra were recorded with a LTQ Orbitrap Discovery instrument (Thermo Scientific, Dreieich, Germany), possessing an Ionmax ionization source. Flash chromatography was performed on Merck silica gel 60 (0.040–0.063 mm). The purity of all the target compounds that underwent biological evaluation was >95%, as ascertained by elemental analysis. Elemental analyses were undertaken using a PerkinElmer PE 240C elemental analyzer (Norwalk, CT, U.S.) and the measured values for C, H and N were within ± 0.4% of the theoretical values. Analytical thin layer chromatography (TLC) was carried out on precoated (0.25 mm) Merck silica gel F-254 plates (Merck KGa, Darmstadt, Germany). The controlled injection of solutions was performed with the Bioblock Scientific device (Illkirch, France).

### 3.2. Synthesis

7-Chloro-3-iodo-1*H*-pyrrolo[2,3-*c*]pyridine (**7**): This compound is mentioned in a patent [41]; here, we provide the methodology and identification data. To a solution of **6** (1.40 g, 9.15 mmol) in anhydrous methanol (50 mL), *N*-iodosuccinimide (2.80 g, 12.4 mmol) was added, and the mixture was stirred at rt for 1 h. Then, the organic solvent was evaporated under vacuo, and the residue was extracted with ethyl acetate. The organic layer was washed with a 10% sodium thiosulfate aqueous solution, dried (Na_2_SO_4_) and evaporated to dryness to result in **7** (2.56 g, 99%), m.p. 278–279 °C (CH_2_Cl_2_/Et_2_O). ^1^H NMR (600 MHz, DMSO-*d_6_*) δ 12.56-12.40 (brs, 1H, NH), 8.00 (d, *J* = 5.4Hz, 1H, H-5), 7.89 (s, 1H, H-2), 7.31 (d, *J* = 5.4 Hz, 1H, H-4). ^13^C NMR (151 MHz, DMSO-*d_6_*) δ 137.95 (C-5), 136.50 (C-3a), 134.66 (C-2), 133.72 (C-7), 129.68 (C-7a), 114.63 (C-4), 56.54 (C-3). HR-MS (ESI) *m*/*z*: Calcd for C_7_H_5_ClIN_2_: [M + H]^+^ = 278.9181, found 278.9193.

7-Chloro-3-iodo-1-(4-methoxybenzyl)-1*H*-pyrrolo[2,3-*c*]pyridine (**8**): To a solution of **7** (2.40 g, 8.62 mmol) in anhydrous *N*,*N*-dimethylformamide (10 mL), sodium hydride (300 mg, 60% in hexanes) was added under cooling in an argon atmosphere, and the resulting solution was stirred at rt for 15 min. Then, 4-methoxybenzyl chloride (1.40 mL, 10.4 mmol) was added, and the solution was stirred at rt for 90 min. The solvent was vacuum-evaporated, ice-water was added to the residue and the precipitate was filtered and air-dried to provide pure **8** (3.40 g, 99%), m.p. 98–99 °C (CH_2_Cl_2_/Et_2_O). ^1^H NMR (600 MHz, DMSO-*d_6_*) δ 8.06 (s, 1H, H-2), 8.02 (d, *J* = 5.3 Hz, 1H, H-5), 7.34 (d, *J* = 5.3 Hz, 1H, H-4), 7.04 (d, *J* = 8.6 Hz, 2H, methoxybenzyl H-2,6), 6.87 (d, *J* = 8.6 Hz, 2H, methoxybenzyl H-3,5), 5.72 (s, 2H, CH_2_), 3.69 (s, 3H, CH_3_O). ^13^C NMR (151 MHz, DMSO-*d_6_*) δ 158.63 (methoxybenzyl C-4), 139.37 (C-2), 138.56 (C-5), 138.22 (C-3a), 132.76 (C-7), 130.14 (methoxybenzyl C-1), 128.39 (C-7a), 127.66 (methoxybenzyl C-2,6), 115.34 (C-4), 114.08 (methoxybenzyl C-3,5), 56.36 (C-3), 55.03 (CH_3_O), 50.60 (CH_2_). HR-MS (ESI) *m*/*z*: Calcd for C_15_H_13_ClIN_2_O: [M + H]^+^ = 398.9756, found 398.9766.

7-Chloro-1-(4-methoxybenzyl)-3-phenyl-1*H*-pyrrolo[2,3-*c*]pyridine (**9a**): To a solution of **8** (1.00 g, 2.51 mmol) in toluene (40 mL) and absolute ethanol (5 mL), phenylboronic acid (360 mg, 2.95 mmol), potassium carbonate (700 mg, 5.07 mmol) and tetrakis(triphenylphosphine)palladium (70 mg, 60.6 μmol) were added sequentially, and the solution was heated at reflux for 3 h. The solvents were evaporated under vacuo, water was added to the residue and it was extracted with ethyl acetate. The organic layer was dried (Na_2_SO_4_) and concentrated to dryness, and the residue was purified by silica gel column chromatography (cyclohexane/ethyl acetate: 4/1) to give **9a** (770 mg, 88%), m.p. 85–86 °C (CHCl_3_/*n*-hexane). ^1^H NMR (600 MHz, CDCl_3_) δ 8.08 (d, *J* = 5.5 Hz, 1H, H-5), 7.78 (d, *J* = 5.5 Hz, 1H, H-4), 7.55 (d, *J* = 7.4 Hz, 2H, phenyl H-2,6), 7.47-7.44 (m, 3H, H-2, phenyl H-3,5), 7.34 (t, *J* = 7.4 Hz, 1H, phenyl H-4), 7.11 (d, *J* = 8.7 Hz, 2H, methoxybenzyl H-2,6), 6.87 (d, *J* = 8.7 Hz, 2H, methoxybenzyl H-3,5), 5.78 (s, 2H, CH_2_), 3.78 (s, 3H, CH_3_O). ^13^C NMR (151 MHz, CDCl_3_) δ 159.62 (methoxybenzyl C-4), 137.03 (C-5), 135.24 (C-7), 133.57 (phenyl C-1), 133.25 (C-3a), 132.74 (C-2), 129.42 (methoxybenzyl C-1), 129.21 (phenyl C-3,5), 129.13 (C-7a), 128.45 (methoxybenzyl C-2,6), 127.73 (phenyl C-2,6), 127.23 (phenyl C-4), 118.25 (C-3), 114.57 (methoxybenzyl C-3,5), 114.55 (C-4), 55.46 (CH_3_O), 51.80 (CH_2_). HR-MS (ESI) *m*/*z*: Calcd for C_21_H_18_ClN_2_O: [M + H]^+^ = 349.1103, found 349.1115.

7-Chloro-3-(3-fluorophenyl)-1-(4-methoxybenzyl)-1*H*-pyrrolo[2,3-*c*]pyridine (**9b**): This compound was synthesized using a procedure analogous to that of **9a**, starting from **8**. Purification was carried out by silica gel column chromatography (cyclohexane/ethyl acetate: 7/3). Oil, yield: 98%. ^1^H NMR (600 MHz, CDCl_3_) δ 8.11 (d, *J* = 5.5 Hz, 1H, H-5), 7.75 (d, *J* = 5.5 Hz, 1H, H-4), 7.45-7.41 (m, 2H, H-2, fluorophenyl H-5), 7.38-7.34 (m, 1H, fluorophenyl H-6), 7.29-7.26 (m, 1H, fluorophenyl H-2), 7.14 (d, *J* = 8.9 Hz, 2H, methoxybenzyl H-2,6), 7.06-7.01 (m, 1H, fluorophenyl H-4), 6.90 (d, *J*= 8.9 Hz, 2H, methoxybenzyl H-3,5), 5.80 (s, 2H, CH_2_), 3.82 (s, 3H, CH_3_O). ^13^C NMR (151 MHz, CDCl_3_) δ 164.20, 162.57 (fluorophenyl C-3), 159.55 (methoxybenzyl C-4), 138.61 (C-5), 134.48, 134.41 (fluorophenyl C-1), 134.24 (C-7), 131.72 (C-2), 130.64, 130.58 (fluorophenyl C-5), 129.64 (C-7a), 129.37 (methoxybenzyl C-1), 128.55 (C-3a), 128.44 (methoxybenzyl C-2,6), 123.20 (fluorophenyl C-6), 116.70 (C-3), 114.53 (methoxybenzyl C-3,5), 114.43, 114.29 (fluorophenyl C-2), 114.15 (C-4), 113.78, 113.64 (fluorophenyl C-4), 55.44 (CH_3_O), 51.68 (CH_2_). HR-MS (ESI) *m/z*: Calcd for C_21_H_17_ClFN_2_O: [M + H]^+^ = 367.1008, found 367.0995.

1-(4-Methoxybenzyl)-*Ν*,3-diphenyl-1*H*-pyrrolo[2,3-*c*]pyridin-7-amine (**10a**): To a solution of **9a** (120 mg, 0.34 mmol) in anhydrous toluene (2 mL), tris(dibenzylideneacetone)dipalladium(0) (8.0 mg, 8.7 μmol), 2,2′-bis(diphenylphosphino)-1,1′-dinapthalene (16 mg, 0.26 mmol), potassium *tert*-butoxide (60 mg, 0.50 mmol) and aniline (46 μL, 0.50 mmol) were added sequentially, and the solution was heated at reflux for 3.5 h. It was then extracted with ethyl acetate,the organic layer was dried (Na_2_SO_4_) and concentrated to dryness, and the residue was purified by silica gel column chromatography (cyclohexane/ethyl acetate: 1/1) to give **10a** in 87% yield, m.p. 180–181 °C (EtOAc/Et_2_O). ^1^H NMR (600 MHz, Acetone-*d_6_*) δ 7.87 (d, *J* = 5.3 Hz, 1H, H-5), 7.81 (s, 1H, H-2), 7.70 (d, *J* = 7.5 Hz, 2H, 3-phenyl H-2,6), 7.49-7.44 (m, 3H, H-4, 3-phenyl H-3,5), 7.29 (t, *J* = 7.4 Hz, 1H, 3-phenyl H-4), 7.25-7.18 (m, 6H, N^7^-phenyl H-2,6, methoxybenzyl H-2,6, N^7^-phenyl H-3,5), 7.16 (brs, 1H, ΝH), 6.92 (d, *J* = 8.7 Hz, 2H, methoxybenzyl H-3,5), 6.87 (t, *J* = 7.0 Hz, 1H, N^7^-phenyl H-4), 5.69 (s, 2H, CH_2_), 3.75 (s, 3H, CH_3_O). ^13^C NMR (151 MHz, Acetone-*d_6_*) δ 160.62 (methoxybenzyl C-4), 144.01 (N^7^-phenyl C-1), 143.60 (C-7), 138.19 (C-5), 135.83 (3-phenyl C-1), 134.39 (C-3a), 131.95 (C-2), 131.25 (methoxybenzyl C-1), 129.81 (3-phenyl C-3,5), 129.52 (N^7^-phenyl C-3,5), 128.94 (methoxybenzyl C-2,6), 128.02 (3-phenyl C-2,6), 126.99 (3-phenyl C-4), 124.93 (C-7a), 121.51 (N^7^-phenyl C-4), 119.09 (N^7^-phenyl C-2,6), 117.51 (C-3), 115.41 (methoxybenzyl C-3,5), 110.05 (C-4), 55.68 (CH_3_O), 52.53 (CH_2_). HR-MS (ESI) *m/z*: Calcd for C_27_H_24_N_3_O: [M + H]^+^ = 406.1914, found 406.1900. *Anal.* Calcd for C_27_H_23_N_3_O: C, 79.97; H, 5.72; N, 10.36. Found: C, 80.06; H, 5.77; N, 10.21.

3-(3-Fluorophenyl)-1-(4-methoxybenzyl)-*Ν*-phenyl-1*H*-pyrrolo[2,3-*c*]pyridin-7-amine (**10b**): This compound was synthesized using a procedure analogous to that of **10a**, starting from **9b**. Purification was carried out by silica gel column chromatography (dichloromethane/ethyl acetate: 9/1). Yield: 91%, m.p. 165–166 °C (CH_2_Cl_2_/*n*-pentane). ^1^H NMR (600 MHz, Acetone-*d_6_*) δ 7.91 (s, 1H, H-2), 7.89 (d, *J* = 5.4 Hz, 1H, H-5), 7.58-7.55 (m, 1H, fluorophenyl H-6), 7.51-7.48 (m, 2H, H-4, fluorophenyl H-5), 7.47-7.43 (m, 1H, fluorophenyl H-2), 7.25-7.16 (m, 7H, phenyl H-2,6, methoxybenzyl H-2,6, phenyl H-3,5,NH), 7.07-7.03 (m, 1H, fluorophenyl H-4), 6.92 (d, *J* = 8.2 Hz, 2H, methoxybenzyl H-3,5), 6.88 (t, *J* = 7.0 Hz, 1H, phenyl H-4), 5.71 (s, 2H, CH_2_), 3.76 (s, 3H, CH_3_O). ^13^C NMR (151 MHz, Acetone-*d_6_*) δ 165.09, 163.48 (fluorophenyl C-3), 160.67 (methoxybenzyl C-4), 143.89 (phenyl C-1), 143.70 (C-7), 138.51 (C-5), 138.32 (fluorophenyl C-1), 134.16 (C-3a), 132.59 (C-2), 131.65, 131.59 (fluorophenyl C-5), 131.07 (methoxybenzyl C-1), 129.52 (phenyl C-3,5), 128.96 (methoxybenzyl C-2,6), 124.90 (C-7a), 123.80 (fluorophenyl C-6), 121.61 (phenyl C-4), 119.18 (phenyl C-2,6), 116.21 (C-3), 115.44 (methoxybenzyl C-3,5), 114.35, 114.21 (fluorophenyl C-2), 113.52, 113.38 (fluorophenyl C-4), 109.81 (C-4), 55.69 (CH_3_O), 52.65 (CH_2_). HR-MS (ESI) *m/z*: Calcd for C_27_H_23_FN_3_O: [M + H]^+^ = 424.1820, found 424.1804. *Anal.* Calcd for C_27_H_22_FN_3_O: C, 76.58; H, 5.24; N, 9.92. Found: C, 76.73; H, 5.29; N, 9.66.

1-(4-Methoxybenzyl)-*Ν*-(3,4,5-trimethoxyphenyl)-3-phenyl-1*H*-pyrrolo[2,3-*c*]pyridin-7-amine (**10c**): This compound was synthesized using a procedure analogous to that of **10a**, starting from **9a**. Purification was carried out by silica gel column chromatography (cyclohexane/ethyl acetate: 1/1). Yield: 60%, m.p. 136–137 °C (EtOAc/Et_2_O). ^1^H NMR (600 MHz, DMSO-*d_6_*) δ 7.99 (s, 1H, H-2), 7.89 (s, 1H, NH), 7.82 (d, *J* = 5.5 Hz, 1H, H-5), 7.66 (d, *J* = 7.5 Hz, 2H, phenyl H-2,6), 7.46 (t, *J* = 7.6 Hz, 2H, phenyl H-3,5), 7.42 (d, *J* = 5.5 Hz, 1H, H-4), 7.28 (t, *J* = 7.3 Hz, 1H, phenyl H-4), 7.10 (d, *J* = 8.5 Hz, 2H, methoxybenzyl H-2,6), 6.83 (d, *J* = 8.5 Hz, 2H, methoxybenzyl H-3,5), 6.54 (s, 2H, trimethoxyphenyl H-2,6), 5.65 (s, 2H, CH_2_), 3.69 (s, 6H, trimethoxyphenyl CH_3_O-3,5), 3.66 (s, 3H, methoxybenzyl CH_3_O), 3.61 (s, 3H, trimethoxyphenyl CH_3_O-4). ^13^C NMR (151 MHz, DMSO-*d_6_*) δ 158.66 (methoxybenzyl C-4), 152.75 (trimethoxyphenyl C-3,5), 142.70 (C-7), 138.92 (trimethoxyphenyl C-1), 137.02 (C-5), 134.34 (phenyl C-1), 132.77 (C-3a), 131.59 (trimethoxyphenyl C-4), 131.46 (C-2), 130.23 (methoxybenzyl C-1), 128.92 (phenyl C-3,5), 128.13 (methoxybenzyl C-2,6), 126.59 (phenyl C-2,6), 125.92 (phenyl C-4), 123.40 (C-7a), 115.51 (C-3), 114.07 (methoxybenzyl C-3,5), 108.67 (C-4), 96.24 (trimethoxyphenyl C-2,6), 60.12 (trimethoxyphenyl CH_3_O-4), 55.64 (trimethoxyphenyl CH_3_O-3,5), 55.00 (methoxybenzyl CH_3_O), 51.06 (CH_2_). HR-MS (ESI) *m/z*: Calcd for C_30_H_30_N_3_O_4_: [M + H]^+^ = 496.2231, found 496.2224. *Anal.* Calcd for C_30_H_29_N_3_O_4_: C, 72.71; H, 5.90; N, 8.48. Found: C, 72.80; H, 5.94; N, 8.34.

3-(3-Fluorophenyl)-1-(4-methoxybenzyl)-*Ν*-(3,4,5-trimethoxyphenyl)-1*H*-pyrrolo[2,3-*c*]pyridin-7-amine (**10d**): This compound was synthesized using a procedure analogous to that of **10a**, starting from **9b**.The reaction was completed at 20 h. Purification was carried out by silica gel column chromatography (cyclohexane/ethyl acetate: 1/1). Yield: 60%, m.p. 93–94 °C (CH_2_Cl_2_/*n*-pentane). ^1^H NMR (600 MHz, Acetone-*d_6_*) δ 7.91 (s, 1H, H-2), 7.87 (d, *J* = 5.2 Hz, 1H, H-5), 7.56-7.54 (m, 1H, fluorophenyl H-6), 7.49-7.46 (m, 1H, fluorophenyl H-5), 7.45-7.42 (m, 2H, H-4, fluorophenyl H-2), 7.26 (d, *J* = 8.8 Hz, 2H, methoxybenzyl H-2,6), 7.07-7.02 (m, 1H, fluorophenyl H-4), 6.98 (d, *J* = 8.8 Hz, 2H, methoxybenzyl H-3,5), 6.58 (s, 2H, trimethoxyphenyl H-2,6), 5.74 (s, 2H, CH_2_), 3.77 (s, 3H, methoxybenzyl CH_3_O), 3.73 (s, 6H, trimethoxyphenyl CH_3_O-3,5), 3.66 (s, 3H, trimethoxyphenyl CH_3_O-4). ^13^C NMR (151 MHz, Acetone-*d_6_*) δ 165.08, 163.47 (fluorophenyl C-3), 160.76 (methoxybenzyl C-4), 154.40 (trimethoxyphenyl C-3,5), 143.99 (C-7), 139.50 (trimethoxyphenyl C-1), 138.38, 138.32 (fluorophenyl C-1), 133.95 (trimethoxyphenyl C-4), 133.86 (C-3a), 132.64 (C-2), 131.65, 131.59 (fluorophenyl C-5), 131.01 (methoxybenzyl C-1), 128.92 (methoxybenzyl C-2,6), 124.30 (C-7a), 123.79 (fluorophenyl C-6), 116.18(C-3), 115.56 (methoxybenzyl C-3,5), 114.34, 114.20 (fluorophenyl C-2), 113.51, 113.37 (fluorophenyl C-4), 109.16 (C-4), 97.69 (trimethoxyphenyl C-2,6), 60.71 (trimethoxyphenyl CH_3_O-4), 56.40 (trimethoxyphenyl CH_3_O-3,5), 55.72 (methoxybenzyl CH_3_O), 52.77 (CH_2_). HR-MS (ESI) *m/z*: Calcd for C_30_H_29_FN_3_O_4_: [M + H]^+^ = 514.2137, found 514.2114. *Anal.* Calcd for C_30_H_28_FN_3_O_4_: C, 70.16; H, 5.50; N, 8.18. Found: C, 70.01; H, 5.44; N, 8.37.

7-Chloro-1-(4-methoxybenzyl)-3-phenyl-1*H*-pyrrolo[2,3-*c*]pyridin-6-oxide (**11a**): To a solution of **9a** (240 mg, 0.69 mmol) in dichloromethane (5 mL), m-chloroperoxybenzoic acid (180 mg, 1.04 mmol) was added, and the solution was stirred at rt for 72h. The solvent was then vacuum-evaporated, and a saturated sodium bicarbonate solution was added to the residue and extracted with ethyl acetate. The organic layer was dried (Na_2_SO_4_) and concentrated to dryness, and the residue was purified by silica gel column chromatography (ethyl acetate/methanol: 95/5) to yield **11a** (220 mg, 88%), m.p. 160–161 °C (EtOAc/n-pentane). ^1^H NMR (600 MHz, CDCl_3_) δ 8.16 (d, *J* = 6.8 Hz, 1H, H-5), 7.60 (d, *J* = 6.8 Hz, 1H, H-4), 7.49 (d, *J* = 7.6 Hz, 2H, phenyl H-2,6), 7.43 (t, *J* = 7,5 Hz, 2H, phenyl H-3,5), 7.37 (s, 1H, H-2), 7.33 (t, *J* = 7.3 Hz, 1H, phenyl H-4), 7.04 (d, *J* = 7.8 Hz, 2H, methoxybenzyl H-2,6), 6.84 (d, *J* = 7.8 Hz, 2H, methoxybenzyl H-3,5), 5.66 (s, 2H, CH_2_), 3.76 (s, 3H, CH_3_O). ^13^C NMR (151 MHz, CDCl_3_) δ 159.68 (methoxybenzyl C-4), 133.45 (C-5), 132.65 (C-2), 132.11 (phenyl C-4), 130.45 (C-7a), 129.28 (phenyl C-1), 128.79 (phenyl C-3,5), 128.23 (methoxybenzyl C-2,6), 127.67 (phenyl C-2,6), 127.50 (C-7), 126.04 (C-3a), 118.83 (C-3), 114.65 (methoxybenzyl C-3,5), 114.47 (methoxybenzyl C-1), 114.03 (C-4), 55.47 (CH_3_O), 51.83 (CH_2_). HR-MS (ESI) *m/z*: Calcd for C_21_H_18_ClN_2_O_2_: [M + H]^+^ = 365.1052, found 365.1036.

7-Chloro-3-(3-fluorophenyl)-1-(4-methoxybenzyl)-1*H*-pyrrolo[2,3-*c*]pyridin-6-oxide (**11b**): This compound was synthesized using a procedure analogous to that of **11a**, starting from **9b**. Purification was carried out by silica gel column chromatography (ethyl acetate/methanol: 9/1). Yield: 88%, m.p. 124–125 °C (EtOAc/n-pentane). ^1^H NMR (600 MHz, Acetone-*d_6_*) δ 8.23 (d, *J* = 6.7 Hz, 1H, H-5), 7.63 (d, *J* = 6.7 Hz, 1H, H-4), 7.51 (td, *J* = 7.9, 4.2 Hz, 1H, fluorophenyl H-5), 7.41 (s, 1H, H-2), 7.37 (dt, *J* = 7.8, 1.2 Hz, 1H, fluorophenyl H-6), 7.28 (ddd, *J* = 10.0, 2.5, 1.7 Hz, 1H, fluorophenyl H-2), 7.17 (tdd, *J* = 8.4, 2.7, 0.9 Hz, 1H, fluorophenyl H-4), 7.07 (d, *J* = 8.1 Hz, 2H, methoxybenzyl H-2,6), 6.87 (d, *J* = 8.1 Hz, 2H, methoxybenzyl H-3,5), 5.68 (s, 2H, CH_2_), 3.78 (s, 3H, CH_3_O). ^13^C NMR (151 MHz, Acetone-*d_6_*) δ 163.73, 162.11 (fluorophenyl C-3), 159.84 (methoxybenzyl C-4), 136.00, 135.94 (fluorophenyl C-1), 134.63 (C-7), 133.21 (C-5), 132.27 (C-2), 130.23 (C-7a), 130.05, 129.99 (fluorophenyl C-5), 128.24 (methoxybenzyl C-2,6), 125.57 (C-3a), 123.33 (fluorophenyl C-6), 119.10 (C-3), 114.77 (methoxybenzyl C-3,5), 114.47 (methoxybenzyl C-1), 114.43, 114.29 (fluorophenyl C-2), 114.25 (C-4), 114.04, 113.90 (fluorophenyl C-4), 55.48 (CH_3_O), 52.08 (CH_2_). HR-MS (ESI) *m/z*: Calcd for C_21_H_17_ClFN_2_O_2_: [M + H]^+^ = 383.0958, found 383.0967.

1-(4-Methoxybenzyl)-7-(4-methylpiperazin-1-yl)-3-phenyl-1*H*-pyrrolo[2,3-*c*]pyridine-6-oxide (**12a**): To a solution of **11a** (120 mg, 0.33 mmol) in absolute ethanol (5 mL), 1-methylpiperazine (0.20 mL, 2.00 mmol) was added, and the solution was heated in an autoclave at 120 °C for 24 h. The mixture was extracted with ethyl acetate, the organic phase was dried (Na_2_SO_4_) and concentrated to dryness and the residue was purified by silica gel column chromatography (dichloromethane/methanol: 8/2) to give **12a** (40 mg, 50%), m.p. 127–128 °C (EtOAc/*n*-pentane). ^1^H NMR (600 MHz, CD_3_OD) 8.00 (d, *J* = 6.9 Hz, 1H, H-5), 7.85 (d, *J* = 6.9 Hz, 1H, H-4), 7.79 (s, 1H, H-2), 7.62 (d, *J* = 7.7 Hz, 2H, phenyl H-2,6), 7.46 (t, *J* = 7.4 Hz, 2H, phenyl H-3,5), 7.32 (t, *J* = 7.4 Hz, 1H, phenyl H-4), 6.90 (m, 4H, methoxybenzyl H-2,3,5,6), 5.89 (s, 2H, CH_2_), 4.17-4.14 (m, 2H, methylpiperazine H), 3.75 (s, 3H, CH_3_O), 2.89-2.85 (m, 2H, methylpiperazine H), 2.80-2.75 (m, 2H, methylpiperazine H), 2.40 (s, 3H, methylpiperazine CH_3_), 2.24-2.18 (m, 2H, methylpiperazine H). ^13^C NMR (600 MHz, CD_3_OD) 160.65 (methoxybenzyl C-4), 142.92 (C-7), 135.49 (C-2), 134.34 (phenyl C-1), 134.17 (C-5), 131.90 (methoxybenzyl C-1), 131.56 (C-3a), 130.43 (C-7a), 130.14 (phenyl C-3,5), 128.41 (phenyl C-2,6), 128.09 (phenyl C-4), 127.62 (methoxybenzyl C-2,6), 119.51 (C-3), 115.69 (C-4), 115.40 (methoxybenzyl C-3,5), 55.78 (CH_3_O), 55.11 (methylpiperazine C-3,5), 52.30 (CH_2_), 47.81 (methylpiperazine C-2,6), 45.67 (methylpiperazine CH_3_). HR-MS (ESI) *m/z*: Calcd for C_26_H_29_N_4_O_2_: [M + H]^+^ = 429.2286, found 429.2273.

3-(3-Fluorophenyl)-1-(4-methoxybenzyl)-7-(4-methylpiperazin-1-yl)-1*H*-pyrrolo[2,3-*c*]pyridine-6-oxide (**12b**): This compound was synthesized using a procedure analogous to that of **12a**, starting from **11b**. Purification was carried out by silica gel column chromatography (dichloromethane/methanol: 85/15). Yield: 50%, m.p. 91–92 °C (EtOAc/*n*-pentane). ^1^H NMR (600 MHz, CD_3_OD) δ 8.01 (d, *J* = 6.9 Hz, 1H, H-5), 7.85 (d, *J* = 6.9 Hz, 1H, H-4), 7.84 (s, 1H, H-2), 7.49-7.45 (m, 1H, fluorophenyl H-5), 7.48-7.44 (m, 1H, fluorophenyl H-6), 7.39-7.34 (m, 1H, fluorophenyl H-2), 7.07-7.01 (m, 1H, fluorophenyl H-4), 6.90-6.88 (m, 4H, methoxybenzyl H-2,3,5,6), 5.91 (s, 2H, CH_2_), 4.14-4.10 (m, 2H, methylpiperazine H), 3.75 (s, 3H, CH_3_O), 2.77-2.72 (m, 4H, methylpiperazine H), 2.29 (s, 3H, methylpiperazine CH_3_), 2.10-2.06 (m, 2H, methylpiperazine H). ^13^C NMR (151 MHz, CD_3_OD) δ 165.55, 163.93 (fluorophenyl C-3), 160.63 (methoxybenzyl C-4), 143.34 (C-7), 136.81, 136.76 (fluorophenyl C-1), 135.89 (C-2), 134.46 (C-5), 131.95, 131.89 (fluorophenyl C-5), 131.75 (C-7a), 131.69 (C-3a), 129.72 (methoxybenzyl C-1), 127.66 (methoxybenzyl C-2,6), 124.16 (fluorophenyl C-6), 118.20 (C-3), 115.44 (C-4), 115.37 (methoxybenzyl C-3,5), 114.92, 114.77 (fluorophenyl C-2), 114.66, 114.52 (fluorophenyl C-4), 55.78 (CH_3_O), 55.26 (methylpiperazine C-3,5), 52.31 (CH_2_), 48.13 (methylpiperazine C-2,6), 46.10 (methylpiperazine CH_3_). HR-MS (ESI) *m/z*: Calcd for C_26_H_28_FN_4_O_2_: [M + H]^+^ = 447.2191, found 447.2176.

1-(4-Methoxybenzyl)-7-(4-methylpiperazin-1-yl)-3-phenyl-1*H*-pyrrolo[2,3-*c*]pyridine (**13a**): To a solution of **12a** (70 mg, 0.16 mmol) in chloroform (3 mL), phosphorus trichloride (0.10 mL, 0.67 mmol) was added, and the solution was stirred at rt for 20 h. The solvent was then vacuum-evaporated and the mixture was neutralized with a sodium bicarbonate solution and extracted with ethyl acetate. The organic phase was dried (Na_2_SO_4_) and concentrated to dryness, and the residue was purified by silica gel column chromatography (dichloromethane/methanol: 9/1) to give **13a** (50 mg, 72%), m.p. 88–89 °C (Et_2_O/*n*-pentane). ^1^H NMR (600 MHz, CDCl_3_) 8.04 (d, *J* = 5.5 Hz, 1H, H-5), 7.59-7.54 (m, 3H, H-4, phenyl H-2,6), 7.42 (t, *J* = 7.8 Hz, 2H, phenyl H-3,5), 7.30-7.27 (m, 2H, H-2, phenyl H-4), 7.08 (d, *J* = 8.7 Hz, 2H, methoxybenzyl H-2,6), 6.83 (d, *J* = 8.7 Hz, 2H, methoxybenzyl H-3,5), 5.70 (s, 2H, CH_2_), 3.78 (s, 3H, CH_3_O), 3.32-3.25 (brs, 4H, methylpiperazine H-2,6), 3.02-2.57 (brs, 4H, methylpiperazine H-3,5), 2.38 (s, 3H, methylpiperazine CH_3_). ^13^C NMR (600 MHz, CDCl_3_) 159.25 (methoxybenzyl C-4), 150.88 (C-7), 137.69 (C-5), 134.75 (phenyl C-1), 133.87 (C-3a), 130.62 (methoxybenzyl C-1), 129.77 (C-2), 129.02 (phenyl C-3,5), 128.45 (methoxybenzyl C-2,6), 127.51 (phenyl C-2,6), 126.56 (phenyl C-4), 126.49 (C-7a), 118.30 (C-3), 114.37 (methoxybenzyl C-3,5), 111.55 (C-4), 55.47 (CH_3_O), 55.26 (methylpiperazine C-3,5), 51.14 (methylpiperazine C-2,6), 50.38 (CH_2_), 46.38 (methylpiperazine CH_3_). HR-MS (ESI) *m/z*: Calcd for C_26_H_29_N_4_O: [M + H]^+^ = 413.2336, found 413.2319. *Anal.* Calcd for C_26_H_28_N_4_O: C, 75.70; H, 6.84; N, 13.58. Found: C, 75.52; H, 6.69; N, 13.66.

3-(3-Fluorophenyl)-1-(4-methoxybenzyl)-7-(4-methylpiperazin-1-yl)-1*H*-pyrrolo[2,3-*c*]pyridine (**13b**): This compound was synthesized using a procedure analogous to that of **13a**, starting from **12b**. Purification was carried out by silica gel column chromatography (dichloromethane/methanol: 9/1). Oil, yield: 99%. ^1^H NMR (600 MHz, Acetone-*d_6_*) δ 7.98 (d, *J* = 5.6 Hz, 1H, H-5), 7.85 (s, 1H, H-2), 7.57 (d, *J* = 5.5 Hz, 1H, H-4), 7.52-7.50 (m, 1H, fluorophenyl H-6), 7.47-7.45 (m, 1H, fluorophenyl H-5), 7.41-7.37 (m, 1H, fluorophenyl H-2), 7.21 (d, *J* = 8.5 Hz, 2H, methoxybenzyl H-2,6), 7.06-7.02 (m, 1H, fluorophenyl H-4), 6.83 (d, *J* = 8.5 Hz, 2H, methoxybenzyl H-3,5), 5.76 (s, 2H, CH_2_), 3.72 (s, 3H, CH_3_O), 3.27-3.20 (brs, 4H, methylpiperazine H-2,6), 2.75-2.50 (brs, 4H, methylpiperazine H-3,5), 2.32 (s, 3H, methylpiperazine CH_3_). ^13^C NMR (151 MHz, Acetone-*d_6_*) δ 165.04, 163.43 (fluorophenyl C-3), 160.16 (methoxybenzyl C-4), 151.99 (C-7), 138.71 (C-5), 138.32, 138.26 (fluorophenyl C-1), 134.31 (C-3a), 132.02 (C-2), 131.91 (methoxybenzyl C-1), 131.61, 131.55 (fluorophenyl C-5), 129.48 (methoxybenzyl C-2,6), 127.07 (C-7a), 123.70, 123.69 (fluorophenyl C-6), 117.01 (C-3), 114.85 (methoxybenzyl C-3,5), 114.25, 114.10 (fluorophenyl C-2), 113.50, 113.36 (fluorophenyl C-4), 111.79 (C-4), 55.83 (CH_3_O), 55.55 (methylpiperazine C-3,5), 51.88 (methylpiperazine C-2,6), 51.15 (CH_2_), 46.44 (methylpiperazine CH_3_). HR-MS (ESI) m/z: Calcd for C_26_H_28_FN_4_O: [M + H]^+^ = 431.2242, found 431.2231. *Anal.* Calcd for C_26_H_27_FN_4_O: C, 72.54; H, 6.32; N, 13.01. Found: C, 72.33; H, 6.19; N, 13.26.

*Ν*,3-Diphenyl-1*H*-pyrrolo[2,3-*c*]pyridin-7-amine (**14a**): A solution of **10a** (50 mg, 0.12 mmol) in trifluoroacetic acid (2mL) was heated at 60 °C for 20h. The mixture was then vacuum-evaporated, neutralized with a sodium bicarbonate solution, extracted with ethyl acetate, dried (Na_2_SO_4_) and concentrated to dryness. The residue was purified by silica gel column chromatography (dichloromethane/methanol: 9/1) to give **14a** (yield 99%), m.p. 139-140 °C (EtOAc/Et_2_O). ^1^H NMR (600 MHz, Acetone-*d_6_*) δ 11.69-11.18 (brs, 1H, pyrrole NH), 9.45-8.50 (brs, 1H, pyridinamine NH), 7.89 (d, *J* = 8.0 Hz, 2H, N^7^-phenyl H-2,6), 7.82 (d, *J* = 5.8 Hz, 1H, H-5), 7.77 (s, 1H, H-2), 7.69 (d, *J* = 7.8 Hz, 2H, 3-phenyl H-2,6), 7.45 (t, *J* = 7.5 Hz, 2H, 3-phenyl H-3,5), 7.39 (d, *J* = 5.8 Hz, 1H, H-4), 7.31 (t, *J* = 7.5 Hz, 2H, N^7^-phenyl H-3,5), 7.28 (t, *J* = 7.4 Hz, 1H, 3-phenyl H-4), 6.99 (t, *J* = 7.4 Hz, 1H, N^7^-phenyl H-4). ^13^C NMR (151 MHz, Acetone-*d_6_*) δ 143.78 (C-7), 141.86 (N^7^-phenyl C-1), 136.08 (C-5), 135.36 (3-phenyl C-1), 131.38 (C-3a), 129.79 (3-phenyl C-3,5), 129.76 (N^7^-phenyl C-3,5), 127.93 (3-phenyl C-2,6), 126.95 (3-phenyl C-4), 126.12 (N^7^-phenyl C-4), 122.84 (C-2), 122.52 (C-7a), 120.55 (N^7^-phenyl C-2,6), 118.92 (C-3), 108.00 (C-4). HR-MS (ESI) *m/z*: Calcd for C_19_H_16_N_3_: [M + H]^+^ = 286.1339, found 286.1340. *Anal.* Calcd for C_19_H_15_N_3_: C, 79.98; H, 5.30; N, 14.72. Found: C, 79.77; H, 5.16; N, 14.89.

3-(3-Fluorophenyl)-*Ν*-phenyl-1*H*-pyrrolo[2,3-*c*]pyridin-7-amine (**14b**): This compound was synthesized using a procedure analogous to that of **14a**, starting from **10b**. Purification was carried out by silica gel column chromatography (dichloromethane/methanol: 9/1). Yield: 99%, m.p. 168–169 °C (EtOAc/Et_2_O). ^1^H NMR (600 MHz, Acetone-*d_6_*) δ 11.66-11.23 (brs, 1H, pyrrole NH), 8.94-8.58 (brs, 1H, pyridinamine NH), 7.90-7.85 (m, 4H, H-5, H-2, phenyl H-2,6), 7.57-7.54 (m, 1H, fluorophenyl H-6), 7.51-7.48 (m, 1H, fluorophenyl H-5), 7.46-7.43 (m, 1H, fluorophenyl H-2), 7.40 (d, *J* = 5.6 Hz, 1H, H-4), 7.31 (t, *J* = 7.5 Hz, 2H, phenyl H-3,5), 7.06-7.02 (m, 1H, fluorophenyl H-4), 6.98 (t, *J* = 7.1 Hz, 1H, phenyl H-4). ^13^C NMR (151 MHz, Acetone-*d_6_*) δ 165.10, 163.48 (fluorophenyl C-3), 143.94 (C-5, C-7), 142.19 (phenyl C-1), 138.76, 138.70 (fluorophenyl C-1), 136.40 (C-3a), 131.62, 131.56 (fluorophenyl C-5), 131.14 (C-2), 129.69 (phenyl C-3,5), 126.49 (C-7a), 123.69 (phenyl C-4), 122.72, 122.55 (fluorophenyl C-6), 120.22 (phenyl C-2,6), 117.57 (C-3), 114.24, 114.09 (fluorophenyl C-2), 113.44, 113.30 (fluorophenyl C-4), 107.82 (C-4). HR-MS (ESI) m/z: Calcd for C_19_H_15_FN_3_: [M + H]^+^ = 304.1245, found 304.1245. *Anal.* Calcd for C_19_H_14_FN_3_: C, 75.23; H, 4.65; N, 13.85. Found: C, 75.40; H, 4.71; N, 13.69.

*N*-(3,4,5-trimethoxyphenyl)-3-phenyl-1*H*-pyrrolo[2,3-*c*]pyridin-7-amine (**14c**): This compound was synthesized using a procedure analogous to that of **14a**, starting from **10c**. Purification was carried out by silica gel column chromatography (dichloromethane/methanol: 95/5).Yield: 99%, m.p. 153–154 °C (EtOAc/*n*-pentane). ^1^H NMR (600 MHz, DMSO-*d_6_*) δ 11.56-11.49 (brs, 1H, pyrrole NH), 8.73-8.65 (brs, 1H, pyridinamine NH), 7.90 (s, 1H, H-2), 7.80 (d, *J* = 5.7 Hz, 1H, H-5), 7.68 (d, *J* = 7.6 Hz, 2H, phenyl H-2,6), 7.45 (t, *J* = 7.6Hz, 2H, phenyl H-3,5), 7.30 (d, *J* = 5.7Hz, 1H, H-4), 7.28-7.25 (m, 3H, trimethoxyphenyl H-2,6, phenyl H-4), 3.81 (s, 6H, trimethoxyphenyl CH_3_O-3,5), 3.64 (s, 3H, trimethoxyphenyl CH_3_O-4).^13^C NMR (151 MHz, DMSO-*d_6_*) δ 152.83 (trimethoxyphenyl C-3,5), 142.93 (C-7), 137.89 (trimethoxyphenyl C-1), 136.24 (C-5), 135.06 (phenyl C-1), 131.83 (trimethoxyphenyl C-4), 129.21 (C-3a), 128.91 (phenyl C-3,5), 126.41 (phenyl C-2,6), 125.70 (phenyl C-4), 124.75 (C-2), 121.31 (C-7a), 116.39 (C-3), 106.62 (C-4), 96.13 (trimethoxyphenyl C-2,6), 60.18 (trimethoxyphenyl CH_3_O-4), 55.76 (trimethoxyphenyl CH_3_O-3,5). HR-MS (ESI) m/z: Calcd for C_22_H_20_N_3_O_3_: [M-H]^-^ = 374.1510, found 374.1508. *Anal.* Calcd for C_22_H_21_N_3_O_3_: C, 70.38; H, 5.64; N, 11.19. Found: C, 70.44; H, 5.68; N, 11.11.

3-(3-Fluorophenyl)-*N*-(3,4,5-trimethoxyphenyl)-1*H*-pyrrolo[2,3-*c*]pyridin-7-amine (**14d**): This compound was synthesized using a procedure analogous to that of **14a**, starting from **10d**. Purification was carried out by silica gel column chromatography (dichloromethane/methanol: 95/5).Yield: 99%, m.p. 149–150 °C (EtOAc/*n*-pentane). ^1^H NMR (600 MHz, Acetone-*d_6_*) δ 11.52-10,85 (brs, 1H, pyrrole NH), 8.90-8.14 (brs, 1H, pyridinamine NH), 7.87 (d, *J* = 5.7 Hz, 1H, H-5), 7.82 (s, 1H, H-2), 7.56-7.53 (m, 1H, fluorophenyl H-6), 7.50-7.47 (m, 1H, fluorophenyl H-5), 7.46-7.42 (m, 1H, fluorophenyl H-2), 7.37 (d, *J* = 5.7 Hz, 1H, H-4), 7.27 (s, 2H, trimethoxyphenyl H-2,6), 7.06-7.02 (m, 1H, fluorophenyl H-4), 3.78 (s, 6H, trimethoxyphenyl CH_3_O-3,5), 3.67 (s, 3H, trimethoxyphenyl CH_3_O-4). ^13^C NMR (151 MHz, Acetone-*d_6_*) δ 165.08, 163.47 (fluorophenyl C-3), 154.49 (trimethoxyphenyl C-3,5), 144.26 (C-6), 138.81, 138.75 (fluorophenyl C-1), 138.31 (trimethoxyphenyl C-1), 136.86 (C-5), 134.33 (trimethoxyphenyl C-4), 131.59, 131.54 (fluorophenyl C-5), 131.03 (C-3a), 126.22 (C-2), 123.65 (fluorophenyl C-6), 122.64 (C-7a), 117.47 (C-3), 114.18, 114.04 (fluorophenyl C-2), 113.38, 113.24 (fluorophenyl C-4), 107.61 (C-4), 98.40 (trimethoxyphenyl C-2,6), 60.73 (trimethoxyphenyl CH_3_O-4), 56.40 (trimethoxyphenyl CH_3_O-3,5). HR-MS (ESI) *m/z*: Calcd for C_22_H_21_FN_3_O_3_: [M + H]^+^ = 394.1562, found 394.1561. *Anal.* Calcd for C_22_H_20_FN_3_O_3_: C, 67.17; H, 5.12; N, 10.68. Found: C, 67.33; H, 5.24; N, 10.59.

7-(4-Methylpiperazin-1-yl)-3-phenyl-1*H*-pyrrolo[2,3-*c*]pyridine (**14e**): This compound was synthesized using a procedure analogous to that of **14a**, starting from **13a**. Purification was carried out by silica gel column chromatography (dichloromethane/methanol: 85/15). Yield: 91%, m.p. 220–221 °C (EtOAc/Et_2_O). ^1^H NMR (600 MHz, Acetone-*d_6_*) δ 10.80-10.70 (brs, 1H, ΝH), 7.88 (d, *J* = 5.6 Hz, 1H, H-5), 7.70-7.65 (m, 3H, phenyl H-2,6, H-2), 7.47-7.40 (m, 3H, phenyl H-3,5, H-4), 7.26 (t, *J* = 7.7 Hz, 1H, phenyl H-4), 3.52-3.46 (m, 4H, methylpiperazine H-2,6), 2.64-2.60 (m, 4H, methylpiperazine H-3,5), 2.32 (s, 3H, methylpiperazine CH_3_). ^13^C NMR (600 MHz, Acetone-*d_6_*) δ 150.27 (C-7), 137.95 (C-5), 136.39 (phenyl C-1), 132.33 (C-3a), 129.72 (phenyl C-3,5), 127.91 (phenyl C-2,6), 126.76 (phenyl C-4), 125.24 (C-7a), 125.08 (C-2), 118.36 (C-3), 109.71 (C-4), 55.98 (methylpiperazine C-3,5), 49.52 (methylpiperazine C-2,6), 46.44 (methylpiperazine CH_3_). HR-MS (ESI) *m/z*: Calcd for C_18_H_19_N_4_: [M-H]^-^ =291.1615, found 291.1612. *Anal.* Calcd for C_18_H_20_N_4_: C, 73.94; H, 6.89; N, 19.17. Found: C, 74.03; H, 6.92; N, 19.02.

3-(3-Fluorophenyl)-7-(4-methylpiperazin-1-yl)-1*H*-pyrrolo[2,3-*c*]pyridine (**14f**): This compound was synthesized using a procedure analogous to that of **14a**, starting from **13b**. Purification was carried out by silica gel column chromatography (dichloromethane/methanol: 85/15). Yield: 93%, m.p. 209–210 °C (EtOAc/*n*-pentane). ^1^H NMR (600 MHz, Acetone-*d_6_*) δ 11.08-10.87 (brs, 1H, NH), 7.90 (d, *J* = 5.7 Hz, 1H, H-5), 7.77 (s, 1H, H-2), 7.56-7.52 (m, 1H, fluorophenyl H-6), 7.50-7.40 (m, 3H, fluorophenyl H-5, H-4, fluorophenyl H-2), 7.05-6.98 (m, 1H, fluorophenyl H-4), 3.55-3.48 (m, 4H, methylpiperazine H-2,6), 2.72-2.65 (m, 4H, methylpiperazine H-3,5), 2.36 (s, 3H, methylpiperazine CH_3_). ^13^C NMR (151 MHz, Acetone-*d_6_*) δ 165.07, 163.46 (fluorophenyl C-3), 150.10 (C-7), 138.92, 138.87 (fluorophenyl C-1), 138.21 (C-5), 132.12 (C-3a), 131.54, 131.48 (fluorophenyl C-5), 125.93 (C-7a), 125.33 (C-2), 123.67 (fluorophenyl C-6), 117.06 (C-3), 114.20, 114.05 (fluorophenyl C-2), 113.28, 113.14 (fluorophenyl C-4), 109.62 (C-4), 55.65 (methylpiperazine C-3,5), 49.14 (methylpiperazine C-2,6), 46.02 (methylpiperazine CH_3_). HR-MS (ESI) *m/z*: Calcd for C_18_H_20_FN_4_: [M + H]^+^ = 311.1667, found 311.1670. *Anal.* Calcd for C_18_H_19_FN_4_: C, 69.66; H, 6.17; N, 18.05. Found: C, 69.43; H, 6.14; N, 18.21.

4-Chloro-7-iodo-5-(4-methoxybenzyl)-5*H*-pyrrolo[3,2-*d*]pyrimidine (**22**): This compound was synthesized using a procedure analogous to that of **8**, starting from **21**. Yield: 99%, m.p. 180–181 °C (CH_2_Cl_2_/Et_2_O). ^1^H NMR (600 MHz, CDCl_3_) δ 8.78 (s, 1H, H-2), 7.52 (s, 1H, H-6), 7.08 (d, *J* = 8.6 Hz, 2H, methoxybenzyl H-2,6), 6.88 (d, *J* = 8.6 Hz, 2H, methoxybenzyl H-3,5), 5.64 (s, 2H, methoxybenzyl CH_2_), 3.79 (s, 3H, methoxybenzyl CH_3_O). ^13^C NMR (151 MHz, CDCl_3_) δ 159.88 (methoxybenzyl C-4), 152.65 (C-7a), 150.64 (C-2), 142.62 (C-4), 140.09 (C-6), 128.76 (methoxybenzyl C-2,6), 127.81 (methoxybenzyl C-1), 124.45 (C-4a), 114.69 (methoxybenzyl C-3,5), 57.91 (C-7), 55.45 (CH_3_O), 52.29 (CH_2_). HR-MS (ESI) *m/z*: Calcd for C_14_H_12_ClIN_3_O: [M + H]^+^ = 399.9709, found 399.9718.

4-Chloro-5-(4-methoxybenzyl)-7-phenyl-5*H*-pyrrolo[3,2-*d*]pyrimidine (**23a**): This compound was synthesized using a procedure analogous to that of **9a**, starting from **22**. Purification was carried out by silica gel column chromatography (cyclohexane/ethyl acetate: 9/1). Yield: 94%, m.p. 93–94 °C (CHCl_3_/*n*-hexane). ^1^H NMR (600 MHz, CDCl_3_) δ 8.80 (s, 1H, H-2), 8.00 (d, *J* = 7.4 Hz, 2H, phenyl H-2,6), 7.71 (s, 1H, H-6), 7.45 (t, *J* = 7.7 Hz, 2H, phenyl H-3,5), 7.31 (t, *J* = 7.7 Hz, 1H, phenyl H-4), 7.11 (d, *J* = 8.7 Hz, 2H, methoxybenzyl H-2,6), 6.88 (d, *J* = 8.7 Hz, 2H, methoxybenzyl H-3,5), 5.70 (s, 2H, CH_2_), 3.79 (s, 3H, CH_3_O). ^13^C NMR (151 MHz, CDCl_3_) δ 159.76 (methoxybenzyl C-4), 150.17 (C-2), 149.91 (C-7a), 142.67 (C-4), 133.65 (C-6), 132.13 (phenyl C-1), 128.97 (phenyl C-3,5), 128.53 (methoxybenzyl C-2,6), 127.12 (phenyl C-4), 127.10 (phenyl C-2,6), 124.91 (C-4a), 117.21 (C-7), 114.75 (methoxybenzyl C-1), 114.65 (methoxybenzyl C-3,5), 55.47 (CH_3_O), 51.89 (CH_2_). HR-MS (ESI) *m/z*: Calcd for C_20_H_17_ClN_3_O: [M + H]^+^ = 350.1055, found 350.1065.

4-Chloro-7-(3-fluorophenyl)-5-(4-methoxybenzyl)-5*H*-pyrrolo[3,2-*d*]pyrimidine (**23b**): This compound was synthesized using a procedure analogous to that of **9a**, starting from **22**. Purification was carried out by silica gel column chromatography (cyclohexane/ethyl acetate: 8/2). Yield: 90%, m.p. 88–89 °C (CHCl_3_/*n*-hexane). ^1^H NMR (600 MHz, CDCl_3_) δ 8.81 (s, 1H, H-2), 7.82-7.79 (m, 1H fluorophenyl H-2), 7.79-7.76 (m, 1H, fluorophenyl H-6), 7.71 (s, 1H, H-6), 7.40-7.37 (m, 1H, fluorophenyl H-5), 7.12 (d, *J* = 8.5 Hz, 2H, methoxybenzyl H-2,6), 7.00-6.96 (m, 1H, fluorophenyl H-4), 6.89 (d, *J* = 8.6 Hz, 2H, methoxybenzyl H-3,5), 5.70 (s, 2H, CH_2_), 3.80 (s, 3H, CH_3_O). ^13^C NMR (151 MHz, CDCl_3_) δ 164.10, 162.48 (fluorophenyl C-3), 159.81 (methoxybenzyl C-4), 150.28 (C-2), 149.73 (C-7a), 142.80 (C-4), 134.28, 134.23 (fluorophenyl C-1) 133.77 (C-6), 130.37, 130.31 (fluorophenyl C-5), 128.59 (methoxybenzyl C-2,6), 124.96 (C-4a), 122.44 (fluorophenyl C-6), 115.92 (C-7), 114.75 (methoxybenzyl C-1), 114.69 (methoxybenzyl C-3,5), 113.94, 113.87 (fluorophenyl C-2), 113.79, 113.73 (fluorophenyl C-4), 55.46 (CH_3_O), 51.96 (CH_2_). HR-MS (ESI) *m/z*: Calcd for C_20_H_16_ClFN_3_O: [M + H]^+^ = 368.0961, found 368.0970.

5-(4-Methoxybenzyl)-*Ν*,7-diphenyl-5*H*-pyrrolo[3,2-*d*]pyrimidin-4-amine (**24a**): This compound was synthesized using a procedure analogous to that of **10a**, starting from **23a**. Purification was carried out by silica gel column chromatography (cyclohexane/ethyl acetate: 7/3).Yield: 87%, m.p. 57–58 °C (CH_2_Cl_2_/Et_2_O). ^1^H NMR (600 MHz, Acetone-*d_6_*) δ 8.44 (s, 1H, H-2), 8.28 (d, *J* = 7.6 Hz, 2H, 7-phenyl H-2,6), 8.16 (s, 1H, H-6), 7.49 (d, *J* = 7.9 Hz, 2H, N^4^-phenyl H-2,6), 7.41 (t, *J* = 7.7 Hz, 2H, 7-phenyl H-3,5), 7.33 (brs, 1H, NH), 7.30-7.19 (m, 5H, N^4^-phenyl H-3,5, methoxybenzyl H-2,6, 7-phenyl H-4), 7.06-6.97 (m, 3H, N^4^-phenyl H-4, methoxybenzyl H-3,5), 5.81 (s, 2H, CH_2_), 3.77 (s, 3H, CH_3_O). ^13^C NMR (151 MHz, Acetone-*d_6_*) δ 160.91 (methoxybenzyl C-4), 151.09 (C-2), 149.36 (C-4), 148.44 (C-7a), 140.77 (N^4^-phenyl C-1), 134.92 (7-phenyl C-1), 132.90 (C-6), 130.46 (methoxybenzyl C-1), 129.43 (7-phenyl C-3,5), 129.28 (N^4^-phenyl C-3,5), 128.81 (methoxybenzyl C-2,6), 127.39 (7-phenyl C-2,6), 126.74 (7-phenyl C-4), 123.61 (N^4^-phenyl C-4), 121.63 (N^4^-phenyl C-2,6), 117.03 (C-4a), 116.11 (C-7), 115.71 (methoxybenzyl C-3,5), 55.75 (CH_3_O), 53.24 (CH_2_). HR-MS (ESI) *m/z*: Calcd for C_26_H_23_N_4_O: [M + H]^+^ = 407.1867, found 407.1864. *Anal.* Calcd for C_26_H_22_N_4_O: C, 76.83; H, 5.46; N, 13.78. Found: C, 76.99; H, 5.45; N, 13.60.

7-(3-Fluorophenyl)-5-(4-methoxybenzyl)-*Ν*-phenyl-5*H*-pyrrolo[3,2-*d*]pyrimidin-4-amine (**24b**): This compound was synthesized using a procedure analogous to that of **10a**, starting from **23b**. Purification was carried out by silica gel column chromatography (cyclohexane/ethyl acetate: 8/2). Yield: 85%, m.p. 131–132 °C (CH_2_Cl_2_/Et_2_O). ^1^H NMR (600 MHz, Acetone-*d_6_*) δ 8.46 (s, 1H, H-2), 8.26-8.22 (m, 2H, fluorophenyl H-2, H-6), 8.06-8.02 (m, 1H, fluorophenyl H-6), 7.48 (d, *J* = 7.9 Hz, 2H, phenyl H-2,6), 7.46-7.42 (m, 1H, fluorophenyl H-5), 7.33 (brs, 1H, NH), 7.31-7.24 (m, 4H, phenyl H-3,5, methoxybenzyl H-2,6), 7.05-6.96 (m, 4H, phenyl H-4, methoxybenzyl H-3,5, fluorophenyl H-4), 5.80 (s, 2H, CH_2_), 3.77 (s, 3H, CH_3_O). ^13^C NMR (151 MHz, Acetone-*d_6_*) δ 164.92, 163.32 (fluorophenyl C-3), 160.95 (methoxybenzyl C-4), 151.30 (C-2), 149.25 (C-4), 148.51 (C-7a), 140.63 (phenyl C-1), 137.44, 137.38 (fluorophenyl C-1), 133.43 (C-6), 131.01, 130.95 (fluorophenyl C-5), 130.26 (methoxybenzyl C-1), 129.44 (phenyl C-3,5), 128.84 (methoxybenzyl C-2,6), 123.74 (phenyl C-4), 122.79 (fluorophenyl C-6), 121.74 (phenyl C-2,6), 117.06 (C-4a), 115.74 (methoxybenzyl C-3,5), 114.69 (C-7), 113.93, 113.78 (fluorophenyl C-2), 113.14, 113.00 (fluorophenyl C-4), 55.75 (CH_3_O), 53.34 (CH_2_). HR-MS (ESI) *m/z*: Calcd for C_26_H_22_FN_4_O: [M + H]^+^ = 425.1773, found 425.1767. *Anal.* Calcd for C_26_H_21_FN_4_O: C, 73.57; H, 4.99; N, 13.20. Found: C, 73.73; H, 5.08; N, 13.02.

5-(4-Methoxybenzyl)-*N*-(3,4,5-trimethoxyphenyl)-7-phenyl-5*H*-pyrrolo[3,2-*d*]pyrimidin-4-amine (**24c**): This compound was synthesized using a procedure analogous to that of **10a**, starting from **23a**. Purification was carried out by silica gel column chromatography (cyclohexane/ethyl acetate: 7/3). Yield: 60%, m.p. 187–188 °C (CH_2_Cl_2_/Et_2_O). ^1^H NMR (600 MHz, Acetone-*d_6_*) δ 8.45 (s, 1H, H-2), 8.28 (d, *J* = 8.1 Hz, 2H, phenyl H-2,6), 8.15 (s, 1H, H-6), 7.41 (t, *J* = 7.8 Hz, 2H, phenyl H-3,5), 7.31 (d, *J* = 8.7 Hz, 2H, methoxybenzyl H-2,6), 7.23 (t, *J* = 7.4 Hz, 1H, phenyl H-4), 7.12 (brs, 1H, NH), 7.05 (d, *J* = 8.7 Hz, 2H, methoxybenzyl H-3,5), 6.78 (s, 2H, trimethoxyphenyl H-2,6), 5.80 (s, 2H, CH_2_), 3.79 (s, 3H, methoxybenzyl CH_3_O), 3.78 (s, 6H, trimethoxyphenyl CH_3_O-3,5), 3.68 (s, 3H, trimethoxyphenyl CH_3_O-4). ^13^C NMR (151 MHz, Acetone-*d_6_*) δ 161.00 (methoxybenzyl C-4), 154.22 (trimethoxyphenyl C-3,5), 151.16 (C-2), 149.17 (C-4), 148.48 (C-7a), 136.56 (trimethoxyphenyl C-1), 135.08 (trimethoxyphenyl C-4), 134.93 (phenyl C-1), 132.94 (C-6), 130.45 (methoxybenzyl C-1), 129.28 (phenyl C-3,5), 128.84 (methoxybenzyl C-2,6), 127.39 (phenyl C-2,6), 126.74 (phenyl C-4), 116.91 (C-4a), 116.07 (C-7), 115.83 (methoxybenzyl C-3,5), 99.69 (trimethoxyphenyl C-2,6), 60.71 (trimethoxyphenyl CH_3_O-4), 56.51 (trimethoxyphenyl CH_3_O-3,5), 55.77 (methoxybenzyl CH_3_O), 53.29 (CH_2_). HR-MS (ESI) *m/z*: Calcd for C_29_H_29_N_4_O_4_: [M + H]^+^ = 497.2184, found 497.2165. *Anal.* Calcd for C_29_H_28_N_4_O_4_: C, 70.15; H, 5.68; N, 11.28. Found: C, 69.99; H, 5.51; N, 11.44.

7-(3-Fluorophenyl)-5-(4-methoxybenzyl)-*N*-(3,4,5-trimethoxyphenyl)-5*H*-pyrrolo[3,2-*d*]pyrimidin-4-amine (**24d**): This compound was synthesized using a procedure analogous to that of **10a**, starting from **23b**. Purification was carried out by silica gel column chromatography (cyclohexane/ethyl acetate: 6/4). Yield: 63%, m.p. 96–97 °C (CH_2_Cl_2_/*n*-pentane). ^1^H NMR (600 MHz, Acetone-*d_6_*) δ 8.47 (s, 1H, H-2), 8.26-8.20 (m, 2H, H-6, fluorophenyl H-2), 8.07-8.03 (m, 1H, fluorophenyl H-6), 7.47-7.40 (m, 1H, fluorophenyl H-5), 7.30 (d, *J* = 8.7 Hz, 2H, methoxybenzyl H-2,6), 7.15 (brs, 1H, NH), 7.05 (d, *J* = 8.7 Hz, 2H, methoxybenzyl H-3,5), 7.02-6.97 (m, 1H, fluorophenyl H-4), 6.77 (s, 2H, trimethoxyphenyl H-2,6), 5.81 (s, 2H, CH_2_), 3.79 (s, 3H, methoxybenzyl CH_3_O), 3.78 (s, 6H, trimethoxyphenyl CH_3_O-3,5), 3.67 (s, 3H, trimethoxyphenyl CH_3_O-4). ^13^C NMR (151 MHz, Acetone-*d_6_*) δ 164.92, 163.32 (fluorophenyl C-3), 161.05 (methoxybenzyl C-4), 154.23 (trimethoxyphenyl C-3,5), 151.39 (C-2), 149.07 (C-4), 148.53 (C-7a), 137.46, 137.40 (fluorophenyl C-1), 136.41 (trimethoxyphenyl C-1), 135.17 (trimethoxyphenyl C-4), 133.47 (C-6), 131.02, 130.96 (fluorophenyl C-5), 130.24 (methoxybenzyl C-1), 128.86 (methoxybenzyl C-2,6), 122.78 (fluorophenyl C-6), 116.94 (C-4a), 115.87 (methoxybenzyl C-3,5), 114.65 (C-7), 11393, 113.78 (fluorophenyl C-2), 113.15, 113.01 (fluorophenyl C-4), 99.78 (trimethoxyphenyl C-2,6), 60.71 (trimethoxyphenyl CH_3_O-4), 56.51 (trimethoxyphenyl CH_3_O-3,5), 55.78 (methoxybenzyl CH_3_O), 53.39 (CH_2_). HR-MS (ESI) *m/z*: Calcd for C_29_H_28_FN_4_O_4_: [M + H]^+^ = 515.2090, found 515.2075. *Anal.* Calcd for C_29_H_27_FN_4_O_4_: C, 67.69; H, 5.29; N, 10.89. Found: C, 67.48; H, 5.16; N, 11.03.

5-(4-Methoxybenzyl)-4-(4-methylpiperazin-1-yl)-7-phenyl-5*H*-pyrrolo[3,2-*d*]pyrimidine (**25a**): To a solution of **23a** (30 mg, 0.09 mmol) in dimethylsulfoxide (1 mL), 1-methylpiperazine (0.1 mL, 0.90 mmol) was added, and the solution was heated at 120 °C for 20 h. Then, the mixture was diluted with water and extracted with ethyl acetate. The organic phase was dried (Na_2_SO_4_) and concentrated to dryness, and the residue was purified by silica gel column chromatography (ethyl acetate/methanol: 8/2) to give **25a** in almost quantitative yield. M.p. 150–151 °C (CH_2_Cl_2_/*n*-pentane). ^1^H NMR (600 MHz, Acetone-*d_6_*) δ 8.57 (s, 1H, H-2), 8.22 (d, *J* = 7.6 Hz, 2H, phenyl H-2,6), 8.11 (s, 1H, H-6), 7.37 (t, *J* = 7.7 Hz, 2H, phenyl H-3,5), 7.23-7.19 (m, 3H, phenyl H-4, methoxybenzyl H-2,6), 6.82 (d, *J* = 8.6 Hz, 2H, methoxybenzyl H-3,5), 5.52 (s, 2H, CH_2_), 3.71 (s, 3H, CH_3_O), 3.46-3.37 (brs, 4H, methylpiperazine H-2,6), 2.68-2.60 (brs, 4H, methylpiperazine H-3,5), 2.32 (s, 3H, methylpiperazine CH_3_). ^13^C NMR (151 MHz, Acetone-*d_6_*) δ 160.31 (methoxybenzyl C-4), 156.02 (C-4), 151.09 (C-2), 150.79 (C-7a), 134.67 (phenyl C-1), 132.92 (C-6), 131.00 (methoxybenzyl C-1), 129.67 (phenyl C-3,5), 129.25 (methoxybenzyl C-2,6), 127.29 (phenyl C-2,6), 126.86 (phenyl C-4), 120.77 (C-4a), 117.66 (C-7), 114.90 (methoxybenzyl C-3,5), 55.53 (CH_3_O), 55.46 (methylpiperazine C-3,5), 51.63 (methylpiperazine C-2,6), 50.90 (CH_2_), 46.42 (methylpiperazine CH_3_). HR-MS (ESI) *m/z*: Calcd for C_25_H_28_N_5_O: [M + H]^+^ = 414.2289, found 414.2286. *Anal.* Calcd for C_25_H_27_N_5_O: C, 72.61; H, 6.58; N, 16.94. Found: C, 72.90; H, 6.69; N, 16.63.

7-(3-Fluorophenyl)-5-(4-methoxybenzyl)-4-(4-methylpiperazin-1-yl)-5*H*-pyrrolo[3,2-*d*]pyrimidine (**25b**): This compound was synthesized using a procedure analogous to that of **25a**, starting from **23b**. Purification was carried out by silica gel column chromatography (dichloromethane/methanol: 9/1). Yield: 97%, m.p. 151–152 °C (CH_2_Cl_2_/*n*-pentane). ^1^H NMR (600 MHz, Acetone-*d_6_*) δ 8.58 (s, 1H, H-2), 8.20 (s, 1H, H-6), 8.18-8.14 (m, 1H, fluorophenyl H-2), 8.02-7.97 (m, 1H, fluorophenyl H-6), 7.42-7.35 (m, 1H, fluorophenyl H-5), 7.20 (d, *J* = 8.6 Hz, 2H, methoxybenzyl H-2,6), 6.98-6.93 (m, 1H, fluorophenyl H-4), 6.82 (d, *J* = 8.6 Hz, 2H, methoxybenzyl H-3,5), 5.53 (s, 2H, CH_2_), 3.71 (s, 3H,CH_3_O), 3.50-3.38 (brs, 4H, methylpiperazine H-2,6), 2.67-2.58 (brs, 4H, methylpiperazine, H-3,5), 2.30 (s, 3H, methylpiperazine CH_3_). ^13^C NMR (151 MHz, Acetone-*d_6_*) δ 164.86, 163.26 (fluorophenyl C-3), 160.38 (methoxybenzyl C-4), 156.14 (C-4), 151.28 (C-2), 150.65 (C-7a), 137.18, 137.12 (fluorophenyl C-1), 133.49 (C-6), 131.01, 130.95 (fluorophenyl C-5), 130.85 (methoxybenzyl C-1), 129.69 (methoxybenzyl C-2,6), 122.77 (fluorophenyl C-6), 121.89 (C-7), 116.27 (C-4a), 114.96 (methoxybenzyl C-3,5), 113.84, 113.69 (fluorophenyl C-2), 113.29, 113.15 (fluorophenyl C-4), 55.55 (CH_3_O, methylpiperazine C-3,5), 51.79 (CH_2_), 50.96 (methylpiperazine C-2,6), 46.46 (methylpiperazine CH_3_). HR-MS (ESI) *m/z*: Calcd for C_25_H_27_FN_5_O: [M + H]^+^ = 432.2195, found 432.2181. *Anal.* Calcd for C_25_H_26_FN_5_O: C, 69.59; H, 6.07; N, 16.23. Found: C, 69.80; H, 6.11; N, 16.00.

*Ν*,7-Diphenyl-5*H*-pyrrolo[3,2-*d*]pyrimidin-4-amine (**26a**): This compound was synthesized using a procedure analogous to that of **14a**, starting from **24a**. Purification was carried out by silica gel column chromatography (dichloromethane/ethyl acetate: 7/3). Yield: 99%, m.p. > 250 °C (EtOAc). ^1^H NMR (600 MHz, Acetone-*d_6_*) δ 10.76-10.67 (brs, 1H, pyrrole NH), 8.64-8.58 (brs, 1H, pyrimidinamine NH), 8.54 (s, 1H, H-2), 8.26 (dd, *J* = 8.2, 1.1 Hz, 2H, 7-phenyl H-2,6), 8.02 (s, 1H, H-6), 7.93 (dd, *J* = 7.7, 1.0 Hz, 2H, N-phenyl H-2,6), 7.39 (t, *J* = 7.8 Hz, 2H, 7-phenyl H-3,5), 7.35 (t, *J* = 7.5 Hz, 2H, N-phenyl H-3,5), 7.21 (t, *J* = 7.4 Hz, 1H, 7-phenyl H-4), 7.05 (t, *J* = 7.3 Hz, 1H, N-phenyl H-4). ^13^C NMR (151 MHz, Acetone-*d_6_*) δ 151.09 (C-2), 148.19 (C-4), 147.05 (C-7a), 141.33 (N-phenyl C-1), 135.34 (7-phenyl C-1), 129.64 (7-phenyl C-3,5), 129.24 (N-phenyl C-3,5), 127.27 (7-phenyl C-2,6), 126.63 (7-phenyl C-4), 126.42 (C-6), 123.32 (N-phenyl C-4), 120.81 (N-phenyl C-2,6), 117.10 (C-4a), 116.53 (C-7). HR-MS (ESI) *m/z*: Calcd for C_18_H_15_N_4_: [M + H]^+^ =287.1292, found 287.1296. *Anal.* Calcd for C_18_H_14_N_4_: C, 75.50; H, 4.93; N, 19.57. Found: C, 75.36; H, 4.90; N, 19.69.

7-(3-Fluorophenyl)-*Ν*-phenyl-5*H*-pyrrolo[3,2-*d*]pyrimidin-4-amine (**26b**): This compound was synthesized using a procedure analogous to that of **14a**, starting from **24b**. Purification was carried out by silica gel column chromatography (dichloromethane/ethyl acetate: 75/25). Yield: 96%, m.p. > 250 °C (EtOAc). ^1^H NMR (600 MHz, Acetone-*d_6_*) δ 10.87-10.71 (brs, 1H, pyrrole NH), 8.63-8.58 (brs, 1H, pyrimidinamine NH), 8.55 (s, 1H, H-2), 8.24-8.20 (m, 1H, fluorophenyl H-2), 8.12 (s, 1H, H-6), 8.06-8.02 (m, 1H, fluorophenyl H-6), 7.93 (d, *J* = 7.7 Hz, 2H, phenyl H-2,6), 7.39-7.36 (m, 1H, fluorophenyl H-5), 7.34 (t, *J* = 7.5 Hz, 2H, phenyl H-3,5), 7.06 (t, *J* = 7.3 Hz, 1H, phenyl H-4), 6.99-6.93 (m 1H, fluorophenyl H-4). ^13^C NMR (151 MHz, Acetone-*d_6_*) δ 164.93, 163.33 (fluorophenyl C-3), 151.35 (C-2), 148.25 (C-4), 147.07 (C-7a), 141.23 (phenyl C-2,6), 137.90, 137.84 (fluorophenyl C-1), 130.97, 130.91 (fluorophenyl C-5), 129.67 (phenyl C-3,5), 127.06 (C-6), 123.44 (phenyl C-4), 122.69 (fluorophenyl C-6), 120.88 (phenyl C-2,6), 116.58 (C-4a), 115.74 (C-7), 113.79, 113.64 (fluorophenyl C-2), 113.03, 112.89 (fluorophenyl C-4). HR-MS (ESI) *m/z*: Calcd for C_18_H_14_FN_4_: [M + H]^+^ = 305.1197, found 305.1192. *Anal.* Calcd for C_18_H_13_FN_4_: C, 71.04; H, 4.31; N, 18.41. Found: C, 70.79; H, 4.22; N, 18.60.

*N*-(3,4,5-Trimethoxyphenyl)-7-phenyl-5*H*-pyrrolo[3,2-*d*]pyrimidin-4-amine (**26c**): This compound was synthesized using a procedure analogous to that of **14a**, starting from **24c**. Purification was carried out by silica gel column chromatography (cyclohexane/ethyl acetate: 3/7). Yield: 97%, m.p. > 250 °C (EtOAc). ^1^H NMR (600 MHz, Acetone-*d_6_*) δ 10.85-10.79 (brs, 1H, pyrrole NH), 8.70-8.63 (brs, 1H, pyrimidinamine NH), 8.53 (s, 1H, H-2), 8.26 (d, *J* = 7.9 Hz, 2H, phenyl H-2,6), 8.01 (s, 1H, H-6), 7.39 (t, *J* = 7.5 Hz, 2H, phenyl H-3,5), 7.31 (s, 2H, trimethoxyphenyl H-2,6), 7.21 (t, *J* = 7.2 Hz, 1H, phenyl H-4), 3.84 (s, 6H, trimethoxyphenyl CH_3_O-3,5), 3.71 (s, 3H, CH_3_O-4). ^13^C NMR (151 MHz, Acetone-*d_6_*) δ 154.46 (trimethoxyphenyl C-3,5), 151.17 (C-2), 148.32 (C-4), 147.07 (C-7a), 137.37 (trimethoxyphenyl C-1), 135.43 (phenyl C-1), 134.98 (trimethoxyphenyl C-4), 129.24 (phenyl C-3,5), 127.27 (phenyl C-2,6), 126.59 (phenyl C-4), 126.29 (C-6), 117.05 (C-4a), 116.48 (C-7), 99.14 (trimethoxyphenyl C-2,6), 60.75 (trimethoxyphenyl CH_3_O-4), 56.52 (trimethoxyphenyl CH_3_O-3,5). HR-MS (ESI) *m/z*: Calcd for C_21_H_19_N_4_O_3_: [M-H]^-^ =375.1462, found 375.1456. *Anal.* Calcd for C_21_H_20_N_4_O_3_: C, 67.01; H, 5.36; N, 14.88. Found: C, 66.84; H, 5.29; N, 15.09.

7-(3-Fluorophenyl)-*N*-(3,4,5-trimethoxyphenyl)-5*H*-pyrrolo[3,2-*d*]pyrimidin-4-amine (**26d**): This compound was synthesized using a procedure analogous to that of **14a**, starting from **24d**. Purification was carried out by silica gel column chromatography (chloroform/ethyl acetate: 4/6). Yield: 93%, m.p. 139–140 °C (EtOAc/Et_2_O). ^1^H NMR (600 MHz, Acetone-*d_6_*) δ 10.82-10.66 (brs, 1H, pyrrole NH), 8.58-8.54 (m, 2H, pyrimidinamine NH, H-2), 8.25-8.19 (m, 1H, fluorophenyl H-2), 8.11 (s, 1H, H-6), 8.06-8.02 (m, 1H, fluorophenyl H-6), 7.44-7.38 (m, 1H, fluorophenyl H-5), 7.28 (s, 2H, trimethoxyphenyl H-2,6), 7.00-6.93 (m, 1H, fluorophenyl H-4), 3.84 (s, 6H, trimethoxyphenyl CH_3_O-3,5), 3.71 (s, 3H, CH_3_O-4). ^13^C NMR (151 MHz, Acetone-*d_6_*) δ 164.92, 163.32 (fluorophenyl C-3), 154.46 (trimethoxyphenyl C-3,5), 151.38 (C-2), 148.34 (C-4), 146.94 (C-7a), 137.91, 137.85 (fluorophenyl C-1), 137.16 (trimethoxyphenyl C-1), 135.07 (trimethoxyphenyl C-4), 130.97, 130.91 (fluorophenyl C-5), 126.97 (C-6), 122.68 (fluorophenyl C-6), 116.48 (C-4a), 115.69 (C-7), 113.77, 113.62 (fluorophenyl C-2), 113.02, 112.87 (fluorophenyl C-4), 99.19 (trimethoxyphenyl C-2,6), 60.75 (trimethoxyphenyl CH_3_O-4), 56.52 (trimethoxyphenyl CH_3_O-3,5). HR-MS (ESI) *m/z*: Calcd for C_21_H_18_FN_4_O_3_: [M-H]^-^ = 393.1368, found 393.1370. *Anal.* Calcd for C_21_H_19_FN_4_O_3_: C, 63.95; H, 4.86; N, 14.21. Found: C, 64.12; H, 4.89; N, 14.05.

4-(4-Methylpiperazin-1-yl)-7-phenyl-5*H*-pyrrolo[3,2-*d*]pyrimidine (**26e**): This compound was synthesized using a procedure analogous to that of **14a**, starting from **25a**. Purification was carried out by silica gel column chromatography (dichloromethane/methanol: 85/15). Yield: 92%, m.p. > 250 °C (EtOAc/Et_2_O). ^1^H NMR (600 MHz, Acetone-*d_6_*) δ 11.18-10.91 (brs, 1H, NH), 8.44 (s, 1H, H-2), 8.25 (d, *J* = 7.5 Hz, 2H, phenyl H-2,6), 7.96 (s, 1H, H-6), 7.37 (t, *J* = 7.7 Hz, 2H, phenyl H-3,5), 7.19 (t, *J* = 7.4 Hz, 1H, phenyl H-4), 3.96-3.90 (m, 4H, methylpiperazine H-2,6), 2.82-2.76 (m, 4H, methylpiperazine H-3,5), 2.47 (s, 3H, methylpiperazine CH_3_). ^13^C NMR (151 MHz, Acetone-*d_6_*) δ 151.98 (C-2), 150.94 (C-4), 148.69 (C-7a), 135.32 (phenyl C-1), 129.17 (phenyl C-3,5), 127.38 (phenyl C-2,6), 126.79 (C-6), 126.54 (phenyl C-4), 116.95 (C-4a), 116.67 (C-7), 55.05 (methylpiperazine C-3,5), 46.24 (methylpiperazine C-2,6), 45.45 (methylpiperazine CH_3_). HR-MS (ESI) *m/z*: Calcd for C_17_H_20_N_5_: [M + H]^+^ = 294.1714, found 294.1715. *Anal.* Calcd for C_17_H_19_N_5_: C, 69.60; H, 6.53; N, 23.87. Found: C, 69.82; H, 6.58; N, 23.57.

7-(3-Fluorophenyl)-4-(4-methylpiperazin-1-yl)-5*H*-pyrrolo[3,2-*d*]pyrimidine (**26f**): This compound was synthesized using a procedure analogous to that of **14a**, starting from **25b**. Oil purification was carried out by silica gel column chromatography (dichloromethane/methanol: 7/3). Yield: 91%. ^1^H NMR (600 MHz, Acetone-*d_6_*) δ 11.78-11.05 (brs, 1H, NH), 8.44 (s, 1H, H-2), 8.22-8.16 (m, 1H, fluorophenyl H-2), 8.02 (s, 1H, H-6), 8.00-7.96 (m, 1H, fluorophenyl H-6), 7.42-7.34 (m, 1H, fluorophenyl H-5), 6.97-6.89 (m, 1H, fluorophenyl H-4), 3.98-3.87 (m, 4H, methylpiperazine H-2,6), 2.75-2.67 (m, 4H, methylpiperazine H-3,5), 2.39 (s, 3H, methylpiperazine CH_3_). ^13^C NMR (151 MHz, Acetone-*d_6_*) δ 164.86, 163.26 (fluorophenyl C-3), 152.03 (C-4), 151.14 (C-2), 148.55 (C-7a), 137.89, 137.83 (fluorophenyl C-1), 130.84, 130.78 (fluorophenyl C-5), 127.27 (C-6), 122.76 (fluorophenyl C-6), 116.85 (C-4a), 115.21 (C-7), 113.87, 113.72 (fluorophenyl C-2), 112.89, 112.75 (fluorophenyl C-4), 55.21 (methylpiperazine C-3,5), 46.38 (methylpiperazine C-2,6), 45.68 (methylpiperazine CH_3_). HR-MS (ESI) *m/z*: Calcd for C_17_H_19_FN_5_: [M + H]^+^ = 312.1619, found 312.1610. *Anal.* Calcd for C_17_H_18_FN_5_: C, 65.58; H, 5.83; N, 22.49. Found: C, 65.66; H, 5.87; N, 22.34.

Ethyl 4-(3-fluorophenyl)-3-(hydroxyimino)-2,4-dioxobutanoate (**31b**): To a solution of **30b** (1.28 g, 5.36 mmol) in glacial acetic acid (10 mL), a sodium nitrite solution (3.5 mL, 3.80 M) was added at 10 °C, under the surface of the solution with a flow rate of 0.15 mL/min via a controlled infusion device. The solution was stirred at rt for 1 h and then was neutralized with 25% ammonia solution and extracted with ethyl acetate; the solvent was dried (Na_2_SO_4_) and concentrated to dryness to give pure **31b** that corresponded practically to a sole isomer. Yield: 85%. Amorphous solid. ^1^H NMR (600 MHz, DMSO-*d_6_*) δ 12.59 (s, 1H, OH), 7.81-7.78 (m, 1H, fluorophenyl H-6), 7.67-7.63 (m, 1H, fluorophenyl H-2), 7.58-7.55 (m, 1H, fluorophenyl H-5), 7.50-7.45 (m, 1H, fluorophenyl H-4), 4.38 (q, *J* = 7.2 Hz, 2H, ethylester H-1), 1.26 (t, *J* = 7.2 Hz, 3H, ethylester H-2). ^13^C NMR (151 MHz, DMSO-*d_6_*) δ 192.03 (C-4), 166.20 (C-2), 164.47 (C-1), 162.79, 161.17 (fluorophenyl C-3), 152.09 (C-3), 133.28, 133.23 (fluorophenyl C-1), 130.84, 130.78 (fluorophenyl C-5), 125.44, 125.42 (fluorophenyl C-6), 119.94, 119.80 (fluorophenyl C-4), 115.81, 115.66 (fluorophenyl C-2), 62.76 (ethylester C-1), 13.72 (ethylester C-2). HR-MS (ESI) *m/z*: Calcd for C_12_H_9_FNO_5_: [M-H]^-^ =266.0470, found 266.0469.

Ethyl 4-amino-3-(3-fluorophenyl)-1H-pyrazole-5-carboxylate (**32b**): To a solution of **31b** (1.22 g, 4.57 mmol) in absolute ethanol (8 mL), hydrazine hydrate (0.5 mL, 9.14 mmol) was added dropwise, under cooling, and the resulting solution was heated at 45 °C for 2 h. The solvent was then vacuum evaporated ethyl acetate was added to the residue and it was washed with a 3N hydrochloric acid solution. The aqueous phase was neutralized with a 2N sodium hydroxide solution and extracted with ethyl acetate; the organic phase was dried (Na_2_SO_4_) and concentrated to dryness to give pure **32b** as an amorphous solid. Yield: 40%. ^1^H NMR (600 MHz, DMSO-*d_6_*) δ 13.50–13.15 (brs, 1H, NH), 7.62-7.58 (m, 1H, fluorophenyl H-6), 7.55-7.51 (m, 1H, fluorophenyl H-2), 7.49-7.45 (m, 1H, fluorophenyl H-5), 7.14-7.09 (m, 1H, fluorophenyl H-4), 5.15-4.90 (brs, 2H, NH_2_), 4.32 (q, *J* = 7.2 Hz, 2H, ethylester H-1), 1.33 (t, *J* = 7.2 Hz, 3H, ethylester H-2). ^13^C NMR (151 MHz, DMSO-*d_6_*) δ 163.23, 161.62 (fluorophenyl C-3), 160.32 (C=O), 136.90 (C-5), 135.21 (C-3), 133.44 (fluorophenyl C-1), 130.58, 130.53 (fluorophenyl C-5), 121.49 (fluorophenyl C-6), 118.59 (C-4), 113.46, 113.32 (fluorophenyl C-4), 112.08, 111.93 (fluorophenyl C-2), 59.88 (ethylester C-1), 14.28 (ethylester C-2). HR-MS (ESI) *m/z*: Calcd for C_12_H_11_FN_3_O_2_: [M-H]^-^ = 248.0840, found 248.0836.

3-(3-Fluorophenyl)-1*H*-pyrazolo[4,3-*d*]pyrimidin-7(6*H*)-one (**33b**): To a solution of **32b** (540 mg, 2.16 mmol) in butanol (8 mL), formamidine acetate (500 mg, 4.80 mmol) was added, and the solution was refluxed for 20 h. Upon completion of the reaction, chloroform (10 mL) was added into the flask, and the precipitate was filtered and dried to give pure **33b**. Yield: 61%, m.p. > 250 °C (MeOH). ^1^H NMR (600 MHz, DMSO-*d_6_*) δ 14.70–14.24 (brs, 1H, pyrazole NH), 12.67–12.30 (brs, 1H, pyrimidinone NH), 8.15-8.13 (m, 1H, fluorophenyl H-6), 8.11-8.07 (m, 1H, fluorophenyl H-2), 8.00 (s, 1H, H-5), 7.56-7.51 (m, 1H, fluorophenyl H-5), 7.23-7.18 (m, 1H, fluorophenyl H-4). ^13^C NMR (151 MHz, DMSO-*d_6_*) δ 163.15, 161.54 (fluorophenyl C-3), 153.49 (C-7), 143.65 (C-5), 140.13 (C-3), 136.50 (C-3a), 133.91 (fluorophenyl C-1), 130.75, 130.69 (fluorophenyl C-5), 129.55 (C-7a), 121.86 (fluorophenyl C-6), 114.73, 114.59 (fluorophenyl C-4), 112.43, 112.27 (fluorophenyl C-2). HR-MS (ESI) *m*/*z*: Calcd for C_11_H_6_FN_4_O: [M-H]^-^ = 229.0531, found 229.0540.

7-Chloro-3-phenyl-1*H*-pyrazolo[4,3-*d*]pyrimidine (**34a**): Compound **33a** (100 mg, 0.47 mmol) was added under cooling to phosphorous oxychloride (1.5 mL). Then, *N*,*N*-diisopropylethylamine (122 µL, 0.71 mmol) was added, and the mixture was refluxed for 3 h under argon. The bulk of the volatile material was then vacuum-evaporated, water was added into the flask and the pH was adjusted to 5 upon addition of a saturated sodium bicarbonate solution. The precipitate was filtered, washed with water and air-dried. The residue was purified by silica gel column chromatography (cyclohexane/ethyl acetate: 7/3) to result in **34a** (85 mg, 80%), m.p. 220–221 °C (acetone). ^1^H NMR (600 MHz, DMSO-*d_6_*) δ 14.80–14.55 (brs, 1H, NH), 8.94 (s, 1H, H-5), 8.43 (d, *J* = 7.7 Hz, 2H, phenyl H-2,6), 7.56 (t, *J* = 7.2 Hz, 2H, phenyl H-3,5), 7.46 (t, *J* = 7.5 Hz, 1H, phenyl H-4). ^13^C NMR (151 MHz, DMSO-*d_6_*) δ 150.43 (C-5), 143.94 (C-7), 143.02 (C-3), 142.28 (C-3a), 131.03 (C-7a), 130.65 (phenyl C-1), 128.79 (phenyl C-3,5), 128.76 (phenyl C-4), 126.28 (phenyl C-2,6). HR-MS (ESI) *m*/*z*: Calcd for C_11_H_6_ClN_4_: [M-H]^-^ = 229.0286, found 229.0301.

7-Chloro-3-(3-fluorophenyl)-1*H*-pyrazolo[4,3-*d*]pyrimidine (**34b**): This compound was synthesized using a procedure analogous to that of **34a**, starting from **33b**. Purification was carried out by silica gel column chromatography (cyclohexane/ethyl acetate: 7/3). Yield: 80%, m.p. > 250 °C (EtOAc). ^1^H NMR (600 MHz, DMSO-*d_6_*) δ 13.80–13.54 (brs, 1H, NH), 8.96 (s, 1H, H-5), 8.30-8.27 (m, 1H, fluorophenyl H-6), 8.22-8.18 (m, 1H, fluorophenyl H-2), 7.65-7.59 (m, 1H, fluorophenyl H-5), 7.33-7.27 (m, 1H, fluorophenyl H-4). ^13^C NMR (151 MHz, DMSO-*d_6_*) δ 163.19, 161.58 (fluorophenyl C-3), 150.76 (C-5), 144.41 (C-7), 142.10 (C-3a), 141.52 (C-3), 133.18 (fluorophenyl C-1), 131.29 (C-7a), 131.08, 131.02 (fluorophenyl C-5), 122.27 (fluorophenyl C-6), 115.62, 115.48 (fluorophenyl C-4), 112.74, 112.58 (fluorophenyl C-2). HR-MS (ESI) *m*/*z*: Calcd for C_11_H_5_ClFN_4_: [M-H]^-^ = 247.0192, found 247.1002.

*N*,3-Diphenyl-1*H*-pyrazolo[4,3-*d*]pyrimidin-7-amine (**35a**): Aniline (0.1 mL, 0.85 mmol) was added into a solution of the chloride **34a** (80 mg, 0.34 mmol) in absolute ethanol (5 mL), and the mixture was refluxed under argon for 2 hrs. Upon completion of the reaction, water (20 mL) was added, and the precipitate was filtered under vacuum, washed with water and air-dried. Purification was carried out by silica gel column chromatography (dichloromethane/methanol: 9/1). Yield: 61%, m.p. 247–248 °C (EtOAc). ^1^H NMR (600 MHz, DMSO-*d_6_*) δ 13.20-12.90 (brs, 1H, pyrazole NH), 9.80-9.55 (brs, 1H, pyrimidinamine NH), 8.53 (brs, 1H, H-5), 8.44 (brs, 2H, 3-phenyl H-2,6), 7.93 (brs, 2H, N-phenyl H-2,6), 7.53 (t, *J* = 7.5 Hz, 2H, 3-phenyl H-3,5), 7.45-7.38 (m, 3H, N-phenyl H-3,5, 3-phenyl H-4), 7.13 (t, *J* = 7.4 Hz, 1H, N-phenyl H-4). ^13^C NMR (151 MHz, DMSO-*d_6_*) δ 151.42 (C-5), 146.78 (C-7), 142.13 (C-3), 139.74 (C-3a), 138.91 (N-phenyl C-1), 132.16 (3-phenyl C-1), 128.89 (N-phenyl C-3,5), 128.62 (3-phenyl C-3,5), 128.08 (3-phenyl C-4), 126.15 (3-phenyl C-2,6), 123.23 (N-phenyl C-4), 122.78 (C-7a), 120.10 (N-phenyl C-2,6). HR-MS (ESI) *m*/*z*: Calcd for C_17_H_12_N_5_: [M-H]^-^ = 286.1098, found 286.1095. *Anal.* Calcd for C_17_H_13_N_5_: C, 71.06; H, 4.56; N, 24.38. Found: C, 70.88; H, 4.65; N, 24.44.

3-(3-Fluorophenyl)-*Ν*-phenyl-1*H*-pyrazolo[4,3-*d*]pyrimidin-7-amine (**35b**): This compound was synthesized using a procedure analogous to that of **35a** starting from **34b**. Purification was carried out by silica gel column chromatography (dichloromethane/methanol: 98/2). Yield: 65%, m.p. 237–238 °C (EtOAc). ^1^H NMR (600 MHz, DMSO-*d_6_*) δ 13.35-12.95 (brs, 1H, pyrazole NH), 9.85-9.60 (brs, 1H, pyrimidinamine NH), 8.55 (s, 1H, H-5), 8.30-8.26 (m, 1H, fluorophenyl H-6), 8.25-8.21 (m, 1H, fluorophenyl H-2), 7.93 (brs, 2H, phenyl H-2,6), 7.61-7.55 (m, 1H, fluorophenyl H-5), 7.43 (t, *J* = 7.4 Hz, 2H, phenyl H-3,5), 7.26-7.21 (m, 1H, fluorophenyl H-4), 7.13 (t, *J* = 7.4 Hz, 1H, phenyl H-4). ^13^C NMR (151 MHz, DMSO-*d_6_*) δ 163.18, 161.57 (fluorophenyl C-3), 151.65 (C-5), 146.79 (C-7), 140.81 (C-3), 139.73 (C-3a), 138.83 (phenyl C-1), 134.43 (fluorophenyl C-1), 130.65 (fluorophenyl C-5), 128.86 (phenyl C-3,5), 123.27 (phenyl C-4), 122.87 (C-7a), 121.99 (fluorophenyl C-6), 120.13 (phenyl C-2,6), 114.75, 114.61 (fluorophenyl C-4), 112.57, 112.42 (fluorophenyl C-2). HR-MS (ESI) *m*/*z*: Calcd for C_17_H_11_FN_5_: [M-H]^-^ = 304.1003, found 304.0998. *Anal.* Calcd for C_17_H_12_FN_5_: C, 66.88; H, 3.96; N, 22.94. Found: C, 66.61; H, 4.02; N, 23.09.

3-Phenyl-*N*-(3,4,5-trimethoxyphenyl)-1*H*-pyrazolo[4,3-*d*]pyrimidin-7-amine (**35c**): This compound was synthesized using a procedure analogous to that of **35a** starting from **34a**. Purification was carried out by silica gel column chromatography (dichloromethane/methanol: 95/5). Yield: 68%, m.p. > 250 °C (EtOAc). ^1^H NMR (600 MHz, DMSO-*d_6_*) δ 13.10-12.80 (brs, 1H, pyrazole NH), 9.70-9.50 (brs, 1H, pyrimidinamine NH), 8.53 (s, 1H, H-5), 8.43 (brs, 2H, phenyl H-2,6), 7.53 (t, *J* = 7.1 Hz, 2H, phenyl H-3,5), 7.40 (t, *J* = 7.4 Hz, 1H, phenyl H-4), 7.35-7.20 (brs, 2H, trimethoxyphenyl H-2,6), 3.83 (s, 6H, trimethoxyphenyl CH_3_O-3,5), 3.68 (s, 3H, trimethoxyphenyl CH_3_O-4). ^13^C NMR (151 MHz, DMSO-*d_6_*) δ 152.88 (trimethoxyphenyl C-3,5), 151.44 (C-5), 146.79 (C-7), 142.13 (C-3), 139.67 (C-3a), 134.96 (C-7a), 133.70 (trimethoxyphenyl C-4), 132.18 (phenyl C-1), 128.62 (phenyl C-3,5), 128.08 (phenyl C-4), 126.18 (phenyl C-2,6), 122.75 (trimethoxyphenyl C-1), 98.25 (trimethoxyphenyl C-2,6), 60.13 (trimethoxyphenyl CH_3_O-4), 55.88 (trimethoxyphenyl CH_3_O-3,5). HR-MS (ESI) *m*/*z*: Calcd for C_20_H_18_N_5_O_3_: [M-H]^-^ =376.1415, found 376.1408. *Anal.* Calcd for C_20_H_19_N_5_O_3_: C, 63.65; H, 5.07; N, 18.56. Found: C, 63.90; H, 5.01; N, 18.69.

3-(3-Fluorophenyl)-*N*-(3,4,5-trimethoxyphenyl)-1*H*-pyrazolo[4,3-*d*]pyrimidin-7-amine (**35d**): This compound was synthesized using a procedure analogous to that of **35a** starting from **34b**. Purification was carried out by silica gel column chromatography (dichloromethane/methanol: 98/2). Yield: 57%, m.p. > 250 °C (EtOAc). ^1^H NMR (600 MHz, DMSO-*d_6_*) δ 13.12-12.96 (brs, 1H, pyrazole NH), 9.66-9.54 (brs, 1H, pyrimidinamine NH), 8.54 (s, 1H, H-5), 8.30-8.19 (m, 2H, fluorophenyl H-6, fluorophenyl H-2), 7.60-7.53 (m, 1H, fluorophenyl H-5), 7.30-7.19 (m, 3H, trimethoxyphenyl H-2,6, fluorophenyl H-4), 3.83 (s, 6H, trimethoxyphenyl CH_3_O-3,5), 3.68 (s, 3H, trimethoxyphenyl CH_3_O-4). ^13^C NMR (151 MHz, DMSO-*d_6_*) δ 163.19, 161.58 (fluorophenyl C-3), 152.85 (C-5), 151.73 (trimethoxyphenyl C-3,5), 146.79 (C-7), 140.82 (C-3), 139.65 (C-3a), 134.85 (C-7a), 133.78 (trimethoxyphenyl C-4), 130.76 (trimethoxyphenyl C-1, fluorophenyl C-5), 122.80 (fluorophenyl C-1), 121.97 (fluorophenyl C-6), 114.81, 114.67 (fluorophenyl C-4), 112.52, 112.36 (fluorophenyl C-2), 98.36 (trimethoxyphenyl C-2,6), 60.12 (trimethoxyphenyl CH_3_O-4), 55.88 (trimethoxyphenyl CH_3_O-3,5). HR-MS (ESI) *m*/*z*: Calcd for C_20_H_19_FN_5_O_3_: [M + H]^+^ = 396.1467, found 396.1455. *Anal.* Calcd for C_20_H_18_FN_5_O_3_: C, 60.75; H, 4.59; N, 17.71. Found: C, 60.94; H, 4.46; N, 17.59.

7-(4-Methylpiperazin-1-yl)-3-phenyl-1*H*-pyrazolo[4,3-*d*]pyrimidine (**35e**): This compound was synthesized using a procedure analogous to that of **35a** starting from **34a**. Upon completion of the reaction, the solvent was evaporated and water (20 mL) was added into the flask, followed by extraction with ethyl acetate (3 × 20 mL). The combined organic layers were dried (Na_2_SO_4_) and evaporated to dryness. Purification was carried out by silica gel column chromatography (dichloromethane/methanol: 9/1). Yield: 80%, m.p. 184–185 °C (EtOAc). ^1^H NMR (600 MHz, DMSO-*d_6_*) δ 8.35 (d, *J* = 7.9 Hz, 2H, phenyl H-2,6), 8.30 (s, 1H, H-5), 7.52 (t, *J* = 7.2 Hz, 2H, phenyl H-3,5), 7.38 (t, *J* = 7.5Hz, 1H, phenyl H-4), 4.25-4.10 (brs, 4H, methylpiperazine H-2,6), 2.49-2.46 (m, 4H, methylpiperazine H-3,5), 2.24 (s, 3H, methylpiperazine CH_3_). ^13^C NMR (151 MHz, DMSO-*d_6_*) δ 152.80 (C-7), 151.80 (C-5), 138.33 (C-3a), 134.43 (C-3), 129.56 (C-7a, phenyl C-1), 128.76 (phenyl C-3,5), 128.01 (phenyl C-4), 125.73 (phenyl C-2,6), 54.53 (methylpiperazine C-3,5), 45.59 (methylpiperazine CH_3_), 44.89 (methylpiperazine C-2,6). HR-MS (ESI) *m*/*z*: Calcd for C_16_H_17_N_6_: [M-H]^-^ =293.1520, found 293.1499. *Anal.* Calcd for C_16_H_18_N_6_: C, 65.29; H, 6.16; N, 28.55. Found: C, 65.44; H, 6.08; N, 28.31.

3-(3-Fluorophenyl)-7-(4-methylpiperazin-1-yl)-1*H*-pyrazolo[4,3-*d*]pyrimidine (**35f**): This compound was synthesized using a procedure analogous to that of **35a** starting from **34b**. The work-up procedure was similar to that of the derivative **35e**. Purification was carried out by silica gel column chromatography (dichloromethane/methanol: 93/7). Yield: 80%, m.p. 212–213 °C (EtOAc). ^1^H NMR (600 MHz, DMSO-*d_6_*) δ 8.32 (s, 1H, H-5), 8.22-8.19 (m, 2H, fluorophenyl H-2, fluorophenyl H-6), 7.58-7.53 (m, 1H, fluorophenyl H-5), 7.22-7.18 (m, 1H, fluorophenyl H-4), 4.18-4.12 (brs, 4H, methylpiperazine H-2,6), 2.48-2.45 (m, 4H, methylpiperazine H-3,5), 2.23 (s, 3H, methylpiperazine CH_3_). ^13^C NMR (151 MHz, DMSO-*d_6_*) δ 163.17, 161.56 (fluorophenyl C-3), 152.52 (C-7), 152.03 (C-5), 138.82 (C-3a), 133.93 (C-3), 132.01, 131.96 (fluorophenyl C-5), 130.84, 130.78 (fluorophenyl C-1), 129.23 (C-7a), 121.58 (fluorophenyl C-6), 114.64, 114.50 (fluorophenyl C-4), 112.21, 112.06 (fluorophenyl C-2), 54.48 (methylpiperazine C-3,5), 45.55 (methylpiperazine CH_3_), 44.92 (methylpiperazine C-2,6). HR-MS (ESI) *m*/*z*: Calcd for C_16_H_16_FN_6_: [M-H]^-^ =311.1425, found 311.1455. *Anal.* Calcd for C_16_H_17_FN_6_: C, 61.53; H, 5.49; N, 26.91. Found: C, 61.61; H, 5.36; N, 27.00.

### 3.3. Cell Viability Assays

The human HCT116 colon cancer cell line and PC-3 prostate cancer cell line were obtained from the American Type Cell Culture (ATCC, Bethesda, Md). Both cell lines were grown in 75 cm^2^ culture flasks at 37 °C in 5% CO_2_ using Roswell Park Memorial Institute 1640 medium (RPMI 1640, Gibco) and Dulbecco’s modified Eagle’s medium F/12 (DMEM/F-12, Gibco), respectively, containing 10% fetal bovine serum (FBS, Gibco). To test the inhibitory activities of compounds using a cell-based assay, MTT assay was performed for cell viability, as described previously [42]. Briefly, HCT116 cells were plated at a density of 1500 per well, while PC-3 cells were plated at a density of 750 per well in a 96-well plate. After 24 h, cells were treated with the indicated compounds in a dose-dependent manner for 72 h and 96 h (all tested compounds provided clear solutions). MTT [3-(4,5-dimethylthiazol-2-yl)-2,5-diphenyltetrazolium bromide] (Sigma M-5655) was added at a final concentration of 0.5 mg/mL directly to each well for 4 h at 37 °C. The medium was aspirated, and the blue MTT formazan precipitate was dissolved in dimethyl sulfoxide (DMSO). Absorbance was determined in a Powerwave microplate spectrophotometer (Biotek Instruments, Inc.) at 540 nm. Viable cell numbers were determined by tetrazolium conversion to its formazan dye. The IC_50_ was calculated by Microsoft Excel equation and confirmed by GraphPad Prism (7.0). Each experiment was performed in triplicate and mean values ± SD are reported.

### 3.4. DNA Staining and Flow Cytometric Analysis

Exponentially growing PC-3 cells were treated with the IC_50_ values of the compounds **14b, 26b** and **35b** or the corresponding DMSO concentration (vehicle) for 72 h. For cell cycle analysis, cell culture supernatants and attached cells were collected, centrifuged, washed in PBS, fixed in 50% ethanol and stained with an RNase-containing propidium iodide solution (50 μg/mL) (all reagents from Sigma). For cell apoptosis assay, cells were harvested and stained with Annexin V binding buffer, Annexin V–FITC and 7-AAD (Annexin V-FITC Apoptosis Detection Kit, BD Systems). DNA content was analyzed on a BD Accuri C6 Flow Cytometer using the BD CSampler software (BD Biosciences, USA). Non-apoptotic events were used to calculate the percentage of cells distributed in each phase. A *p* value < 0.05 was considered to be statistically significant (Student’s *t*-test).

## 4. Conclusions

In conclusion, we have designed and synthesized a number of new substituted pyrrolo[2,3-*c*]pyridines, pyrrolo[3,2-*d*]pyrimidines and pyrazolo[4,3-*d*]pyrimidines and have evaluated their antiproliferative activity against two cancer cell lines. We preserved an analogous substitution pattern around the fused ring-system of all three scaffolds in order to assist the extraction of SARs. We have identified a number of derivatives with potent cytotoxic properties—more precisely, the pyrrolopyridines **14a–f**, the pyrrolopyrimidines **26a,b** and the pyrazolopyrimidines **35a,b**, all of which possess IC_50_ values in the nM to low µM level against both cell lines tested. We assume that in all cases the simultaneous presence of a 3-fluorophenyl moiety and a phenylamino substituent at analogous positions resulted in highly active compounds which induce apoptosis in PC-3 cells and, importantly, are devoid of toxicity against normal cells. These interesting observations have triggered the continuation of research efforts in our laboratory for the investigation of the exact mechanism of action of this promising class of compounds.

## Figures and Tables

**Table 1:** Accumulative results of the antiproliferative activities of all compounds tested.

Compd	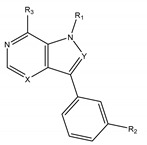					IC_50_ Values (μΜ) ^a^	
	X	Y	R_1_^b^	R_2_	R_3_^c^	PC-3	HCT116
**10a**	CH	CH	PMB	H	PhNH	>10	2.4 ± 0.1
**10b**	CH	CH	PMB	F	PhNH	>10	>10
**10c**	CH	CH	PMB	H	TMPNH	9.80 ± 0.25	9.40 ± 0.53
**10d**	CH	CH	PMB	F	TMPNH	>10	>10
**13a**	CH	CH	PMB	H	PIP	7.00 ± 0.40	5.00 ± 0.18
**13b**	CH	CH	PMB	F	PIP	9.30 ± 0.76	4.40 ± 0.35
**14a**	CH	CH	H	H	PhNH	0.215 ± 0.025	5.00 ± 0.10
**14b**	CH	CH	H	F	PhNH	0.055 ± 0.015	0.09 ± 0.01
**14c**	CH	CH	H	H	TMPNH	0.47 ± 0.12	0.72 ± 0.09
**14d**	CH	CH	H	F	TMPNH	1.45 ± 0.25	3.90 ± 0.30
**14e**	CH	CH	H	H	PIP	1.75 ± 0.25	2.85 ± 0.05
**14f**	CH	CH	H	F	PIP	2.35 ± 0.05	2.35 ± 0.15
**24a**	N	CH	PMB	H	PhNH	>10	>10
**24b**	N	CH	PMB	F	PhNH	>10	>10
**24c**	N	CH	PMB	H	TMPNH	2.25 ± 0.35	4.50 ± 0.25
**24d**	N	CH	PMB	F	TMPNH	>10	>10
**25a**	N	CH	PMB	H	PIP	>10	>10
**25b**	N	CH	PMB	F	PIP	>10	>10
**26a**	N	CH	H	H	PhNH	6.20 ± 0.23	2.75 ± 0.35
**26b**	N	CH	H	F	PhNH	1.95 ± 0.05	2.05 ± 0.05
**26c**	N	CH	H	H	TMPNH	9.70 ± 0.50	7.80 ± 0.45
**26d**	N	CH	H	F	TMPNH	5.90 ± 0.37	8.70 ± 0.58
**26e**	N	CH	H	H	PIP	>10	>10
**26f**	N	CH	H	F	PIP	>10	>10
**35a**	N	N	H	H	PhNH	4.70 ± 0.18	7.30 ± 0.62
**35b**	N	N	H	F	PhNH	0.55 ± 0.05	2.30 ± 0.20
**35c**	N	N	H	H	TMPNH	5.70 ± 0.40	4.70 ± 0.25
**35d**	N	N	H	F	TMPNH	>10	>10
**35e**	N	N	H	H	PIP	>10	>10
**35f**	N	N	H	F	PIP	>10	>10
Dox						0.025 ± 0.004	0.095 ± 0.01

^a^ Results presented are means ± standard deviation (SD) of three independent experiments and are expressed as IC_50_, i.e., the effective concentration reducing viability by 50% compared to unexposed control cells; ^b^ PMB: 4-methoxybenzyl, ^c^ PhNH: phenylamine, TMPNH: 3,4,5-trimethoxyphenylamine, PIP: 4-methylpiperazin-1-yl.

**Table 2:** IC_50_ values of the most potent compounds in human fibroblasts.

Compd.	WI-38 Fibroblasts IC_50_ (μΜ)	Selectivity Index ^a^ PC-3	Selectivity Index ^a^ HCT116
**14a**	<10		
**14b**	20 ± 0.4	363	222
**14c**	<10		
**14d**	<10		
**14e**	<10		
**26b**	17 ± 1.5	8.7	8.3
**35b**	12.2 ± 1.0	22.2	5.3

^a^ SI = IC_50_^non cancer cell^/IC_50_^cancer cell.^

**Table 3:** Cell cycle phase distribution at 72 h (%).

Compound	G_0_/G_1_	S	G_2_/M
**14b**	48.7 (±0.6)	20.5 (±1.4)	30.8 (±0.8)
**26b**	54.4 (±1.1)	20.7 (±02.7)	24.9 (±1.6)
**35b**	58.9 (±0.6)	16.3 (±1.8)	24.9 (±2.4)
**vehicle**	57.7 (±1.3)	17.6 (±0.1)	24.8 (±1.5)

## Supplementary Materials

The following supporting information can be downloaded online: Figures S1–S55: ^1^H-NMR and ^13^C NMR spectra of new compounds, HRMS spectra of active compounds; Table S1: Calculated physicochemical characteristics of the tested compounds using SwissADME.

## Sample Availability

Samples of the compounds 14a-f, 26a-f, 35a-f are available from the authors.
