# Supplementary material for: Novel Substituted Purine Isosteres: Synthesis, Structure-Activity Relationships and Cytotoxic Activity Evaluation
Source: Molecules. 2021 Dec 31;27(1):247. doi: 10.3390/molecules27010247 (PMC8746528; doi:10.3390/molecules27010247)
Supplement: Supplementary file 1 [file molecules-27-00247-s001.zip › molecules-1503764-supplementary.pdf]

Novel Substituted Purine Isosteres: Synthesis, Structure-Activity Relationships and Cytotoxic Activity Evaluation.

Spyridon Dimitrakis [1], Efthymios-Spyridon Gavriil [1], Athanasios Pousias [1], Nikolaos Lougiakis [1], Panagiotis Marakos [1,*], Nicole Pouli [1], Katerina Gioti [2] and Roxane Tenta[2]

[1] Division of Pharmaceutical Chemistry, Department of Pharmacy, National and Kapodistrian University of Athens, Panepistimiopolis, Zografou 15771, Greece.

[2] Department of Nutrition & Dietetics, School of Health Sciences and Education, Harokopio University, Athens 17671, Greece

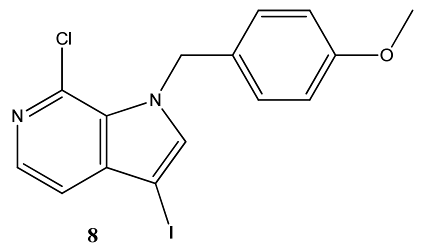

8

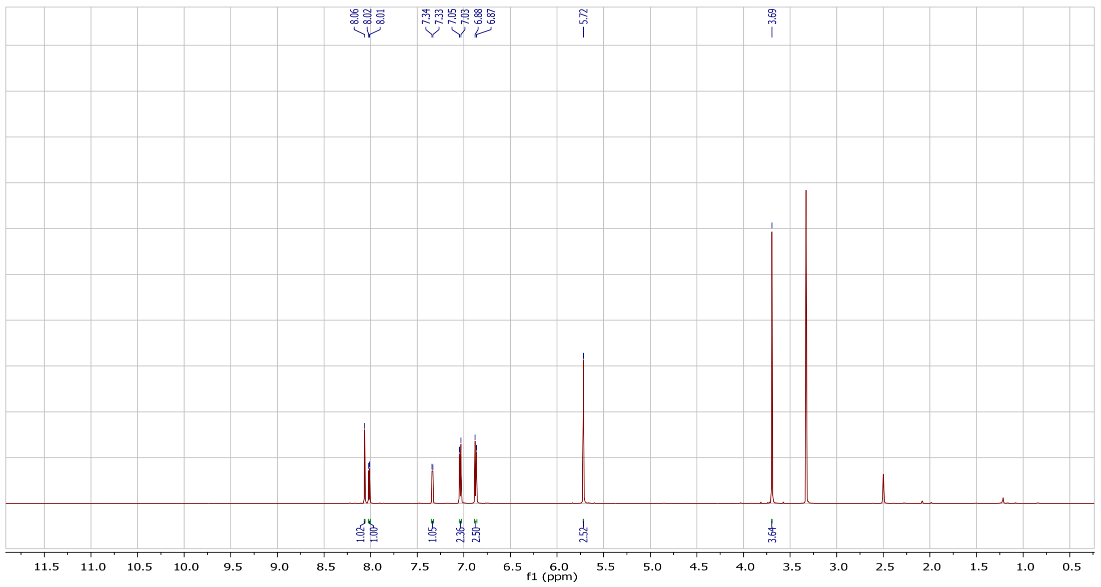

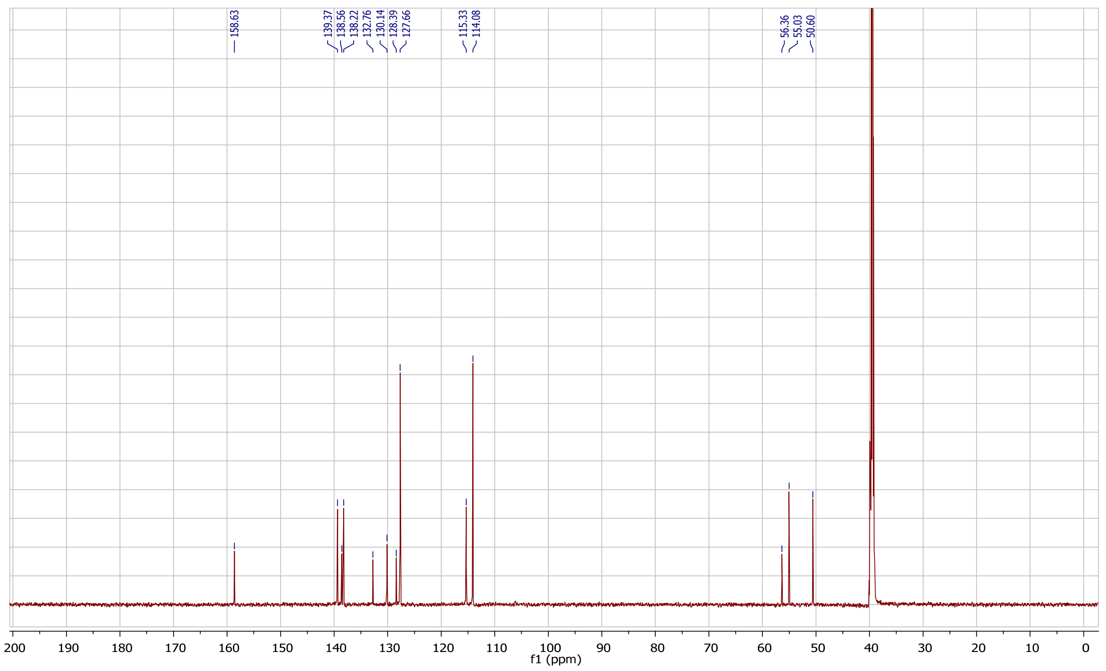

Figure S1. ^{1}H and ^{13}C NMR spectra of compound **8** in DMSO-d_6.

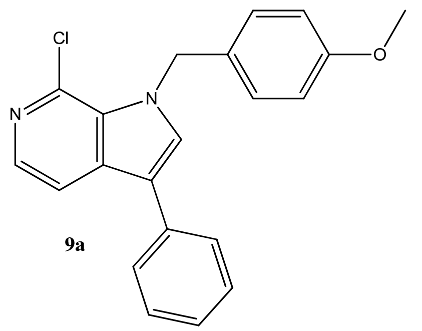

9a

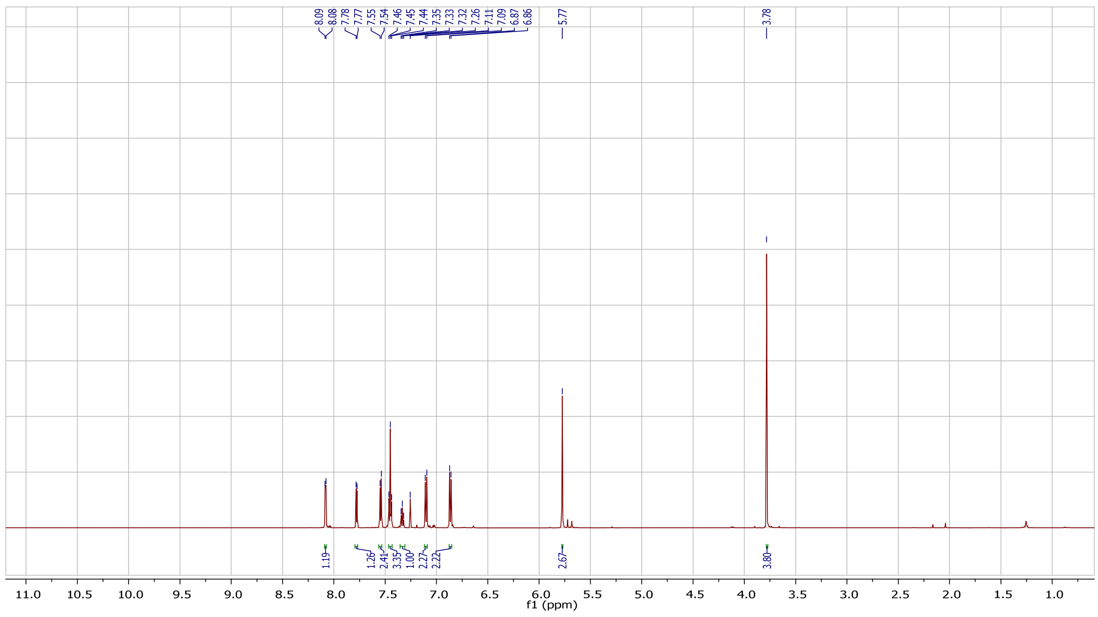

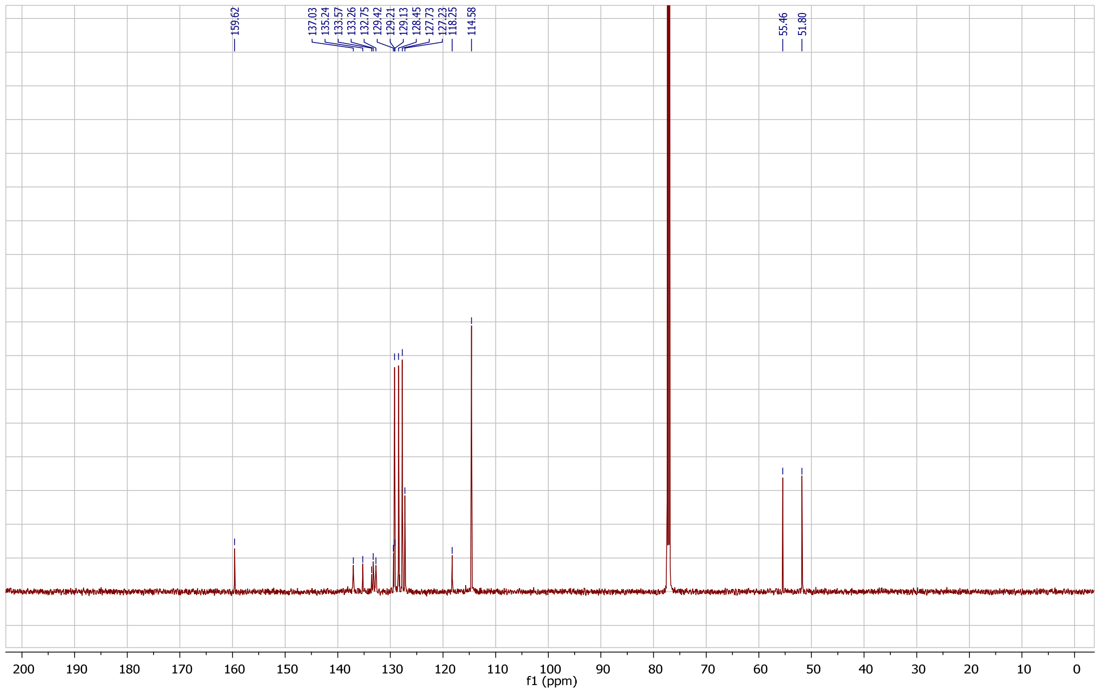

Figure S2. ^{1}H and ^{13}C NMR spectra of compound **9a** in $CDCl_3$.

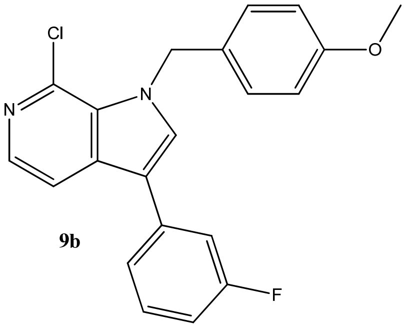

9b

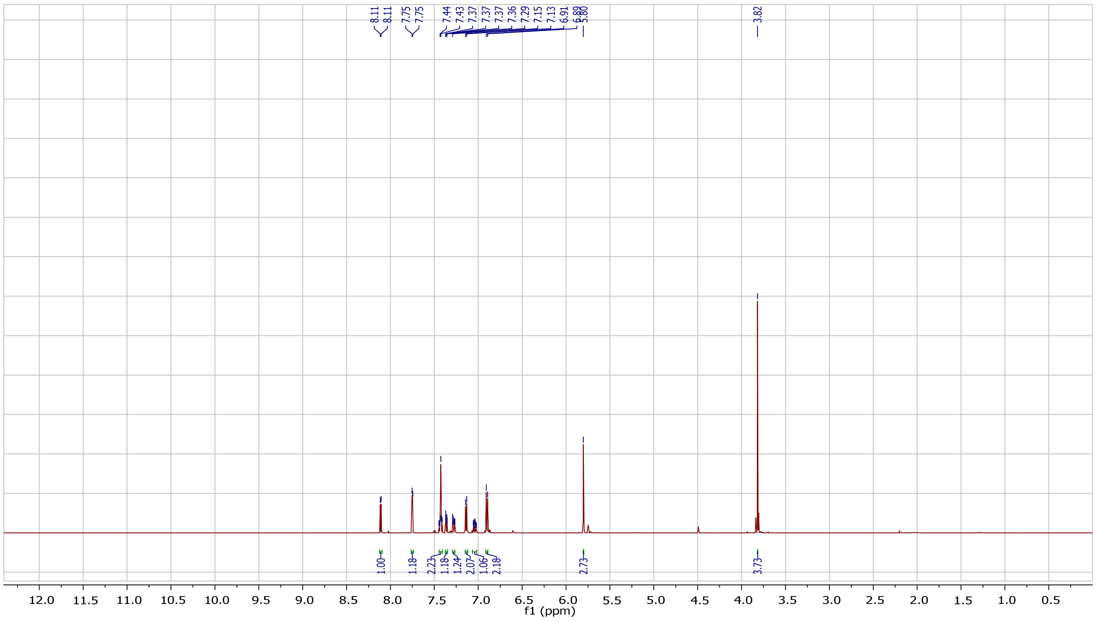

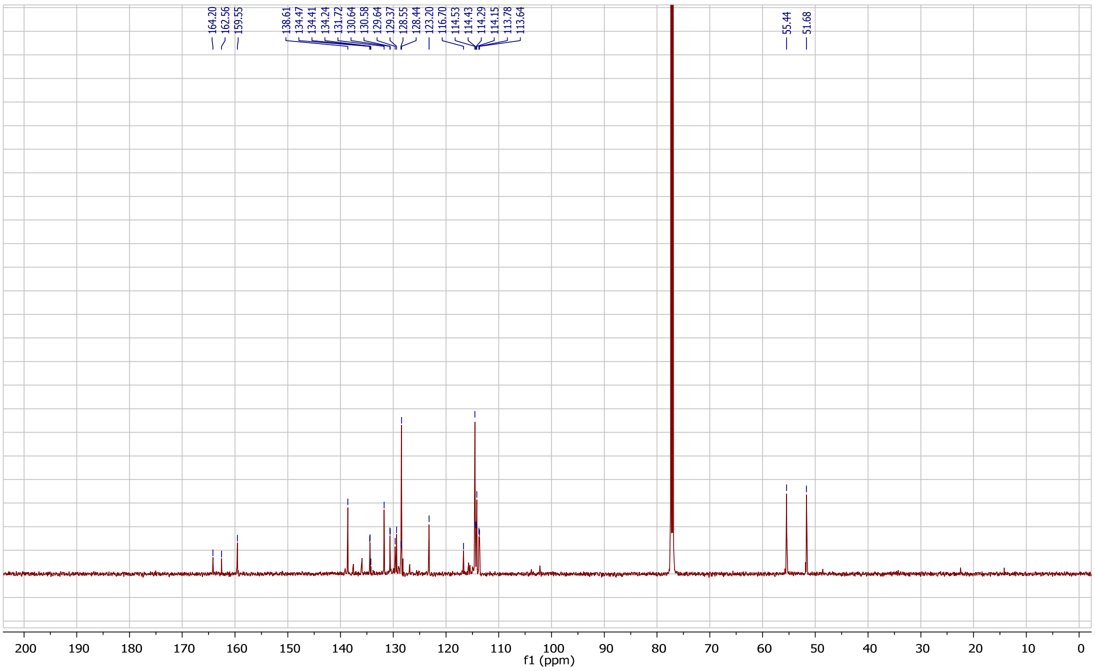

Figure S3. ^{1}H and ^{13}C NMR spectra of compound **9b** in $CDCl_3$.

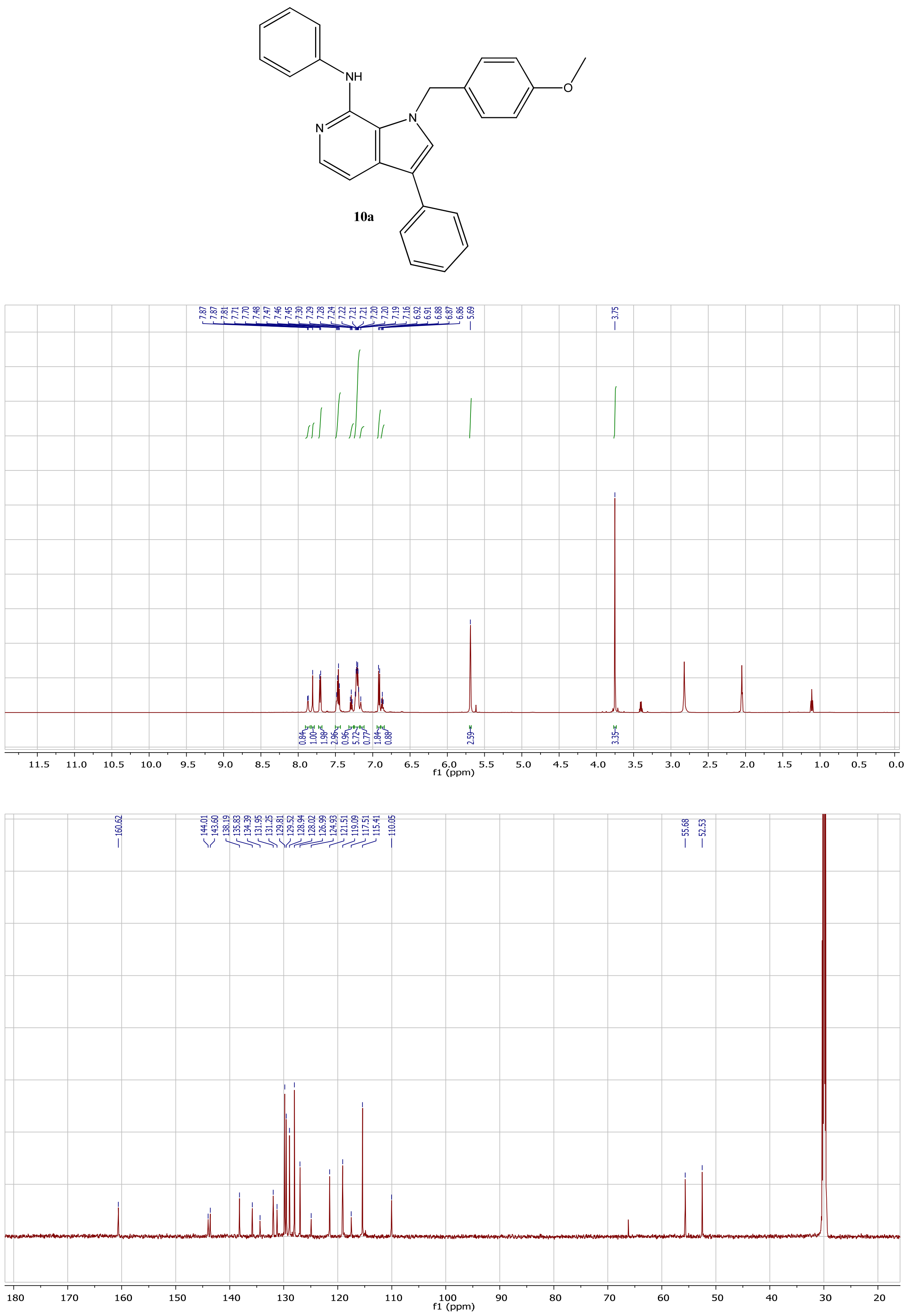

Figure S4. ^{1}H and ^{13}C NMR spectra of compound **10a** in acetone-d_6.

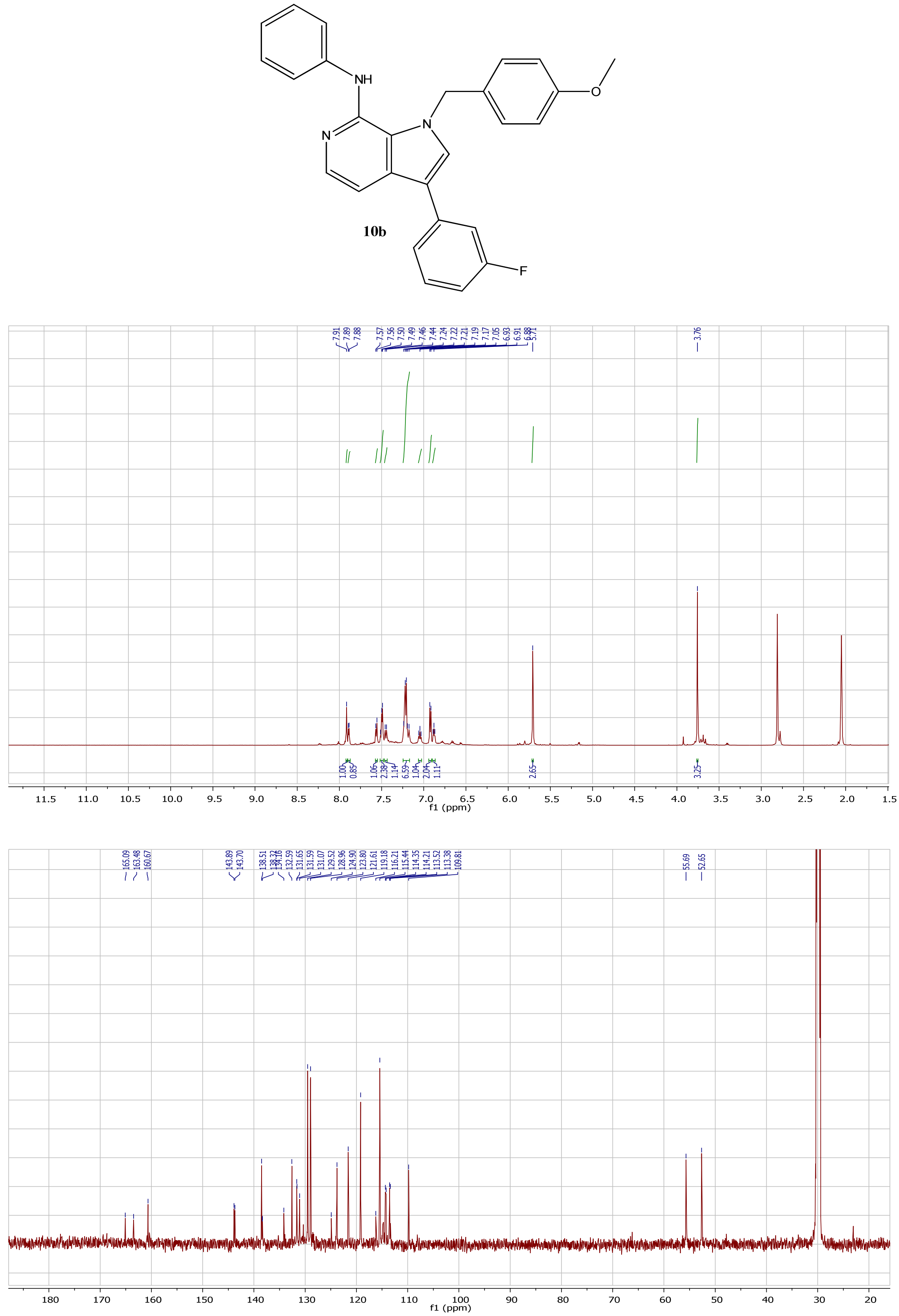

Figure S5. ^{1}H and ^{13}C NMR spectra of compound **10b** in acetone-d_6.

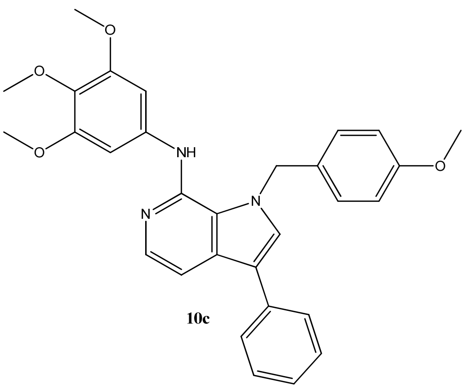

10c

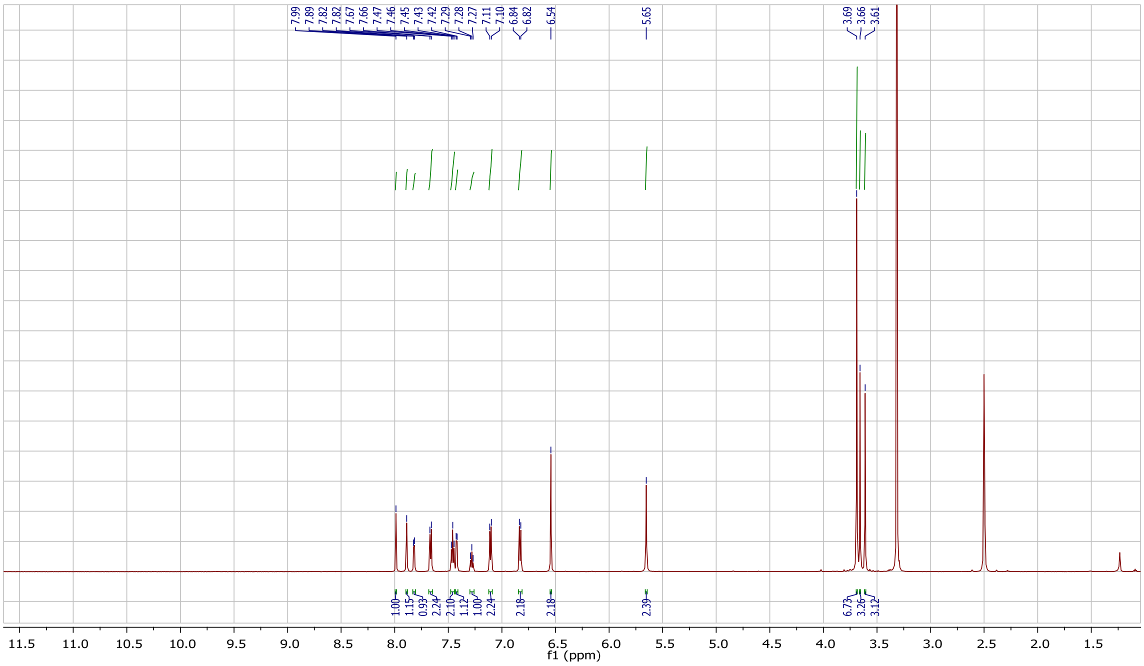

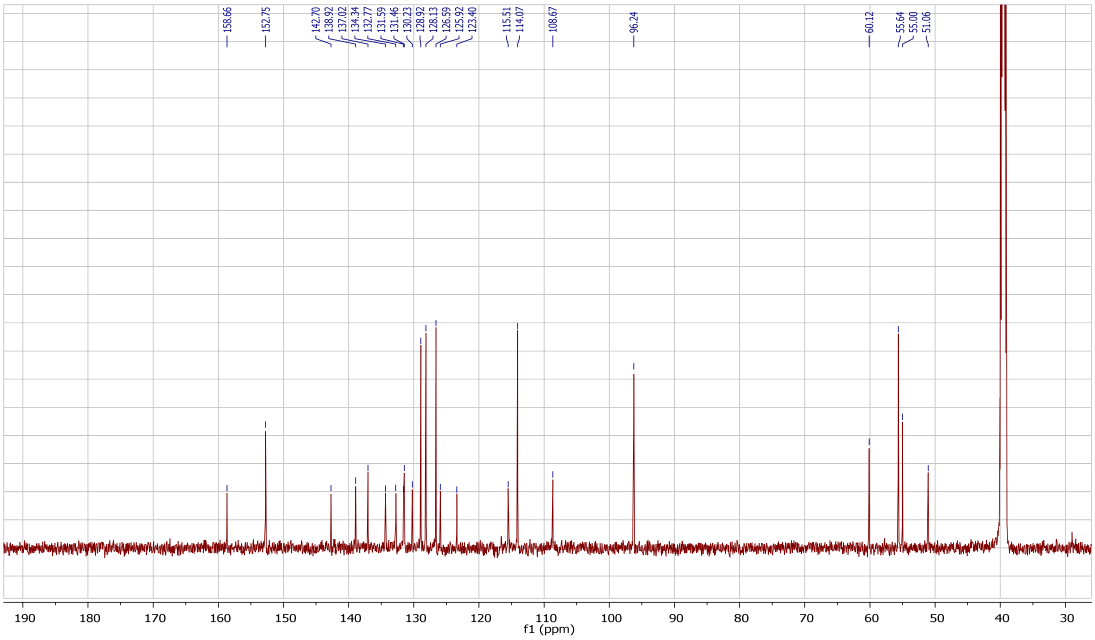

Figure S6. ^{1}H and ^{13}C NMR spectra of compound **10c** in DMSO-d_6.

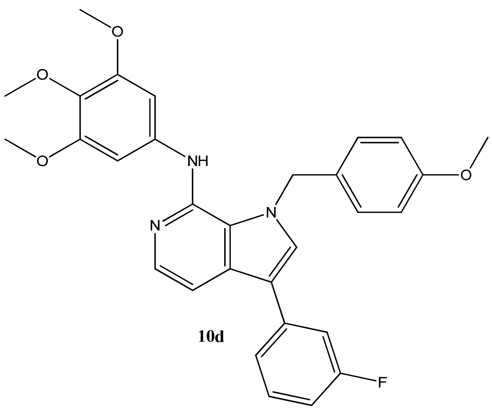

10d

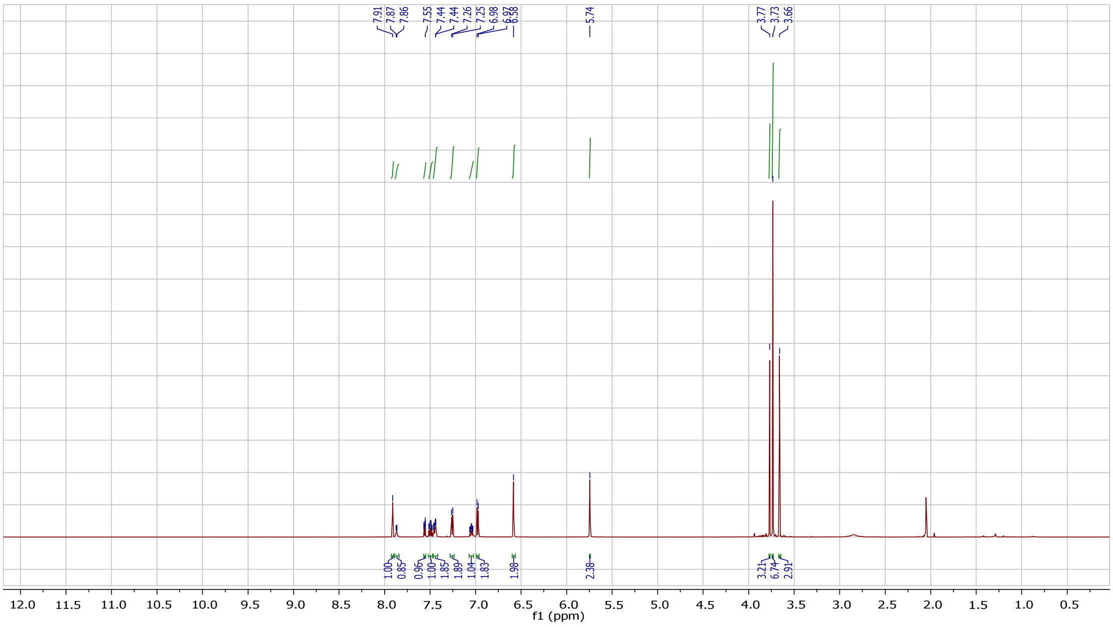

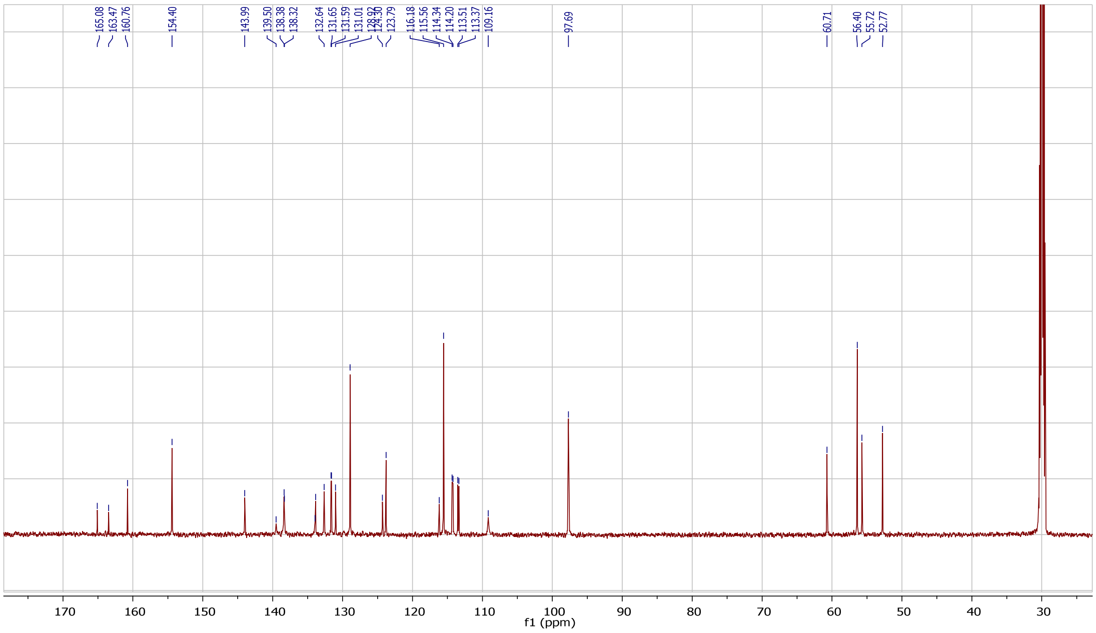

Figure S7. ^{1}H and ^{13}C NMR spectra of compound **10d** in acetone-d_6.

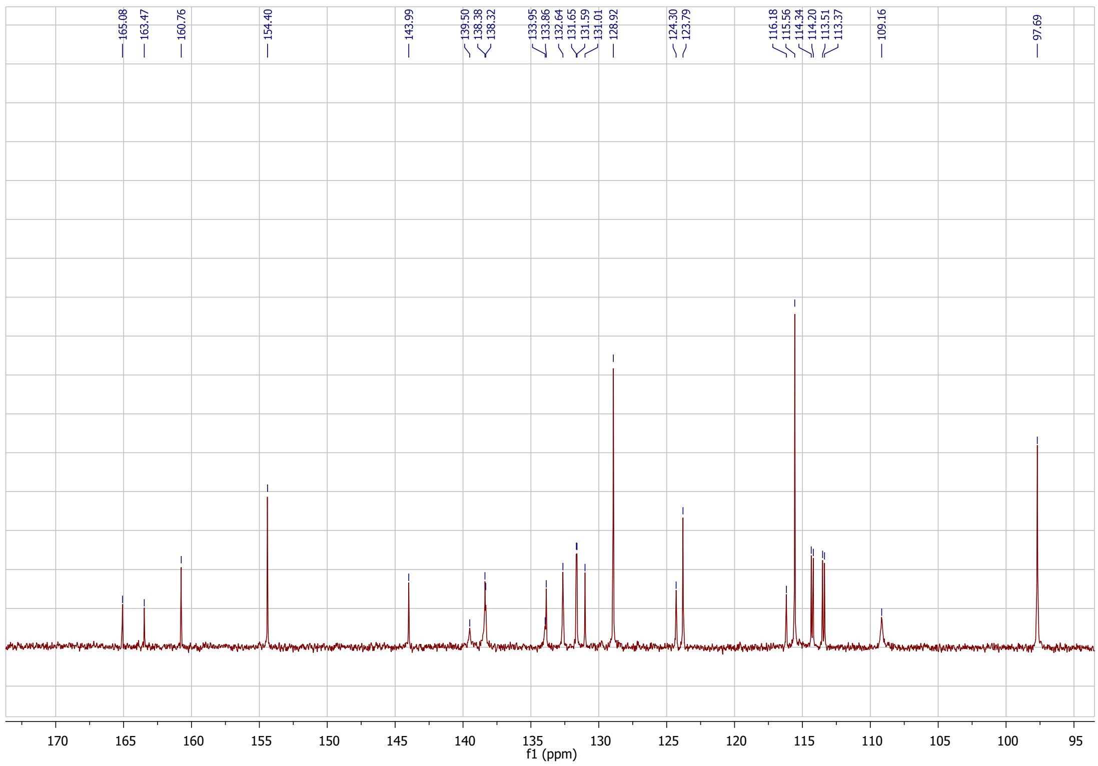

Figure S8. Zoom of the aromatic area of the ^{13}C NMR spectrum of compound **10d** in acetone-*d*$_6$.

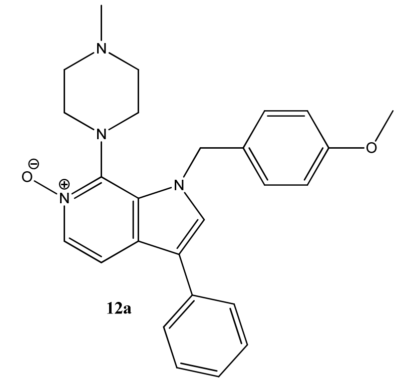

12a

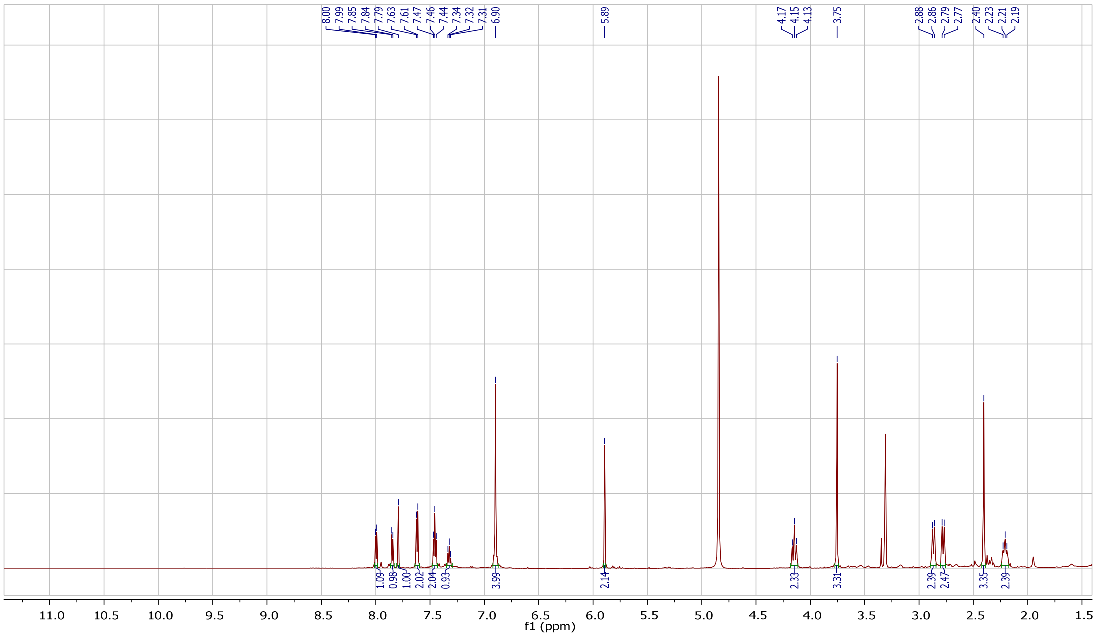

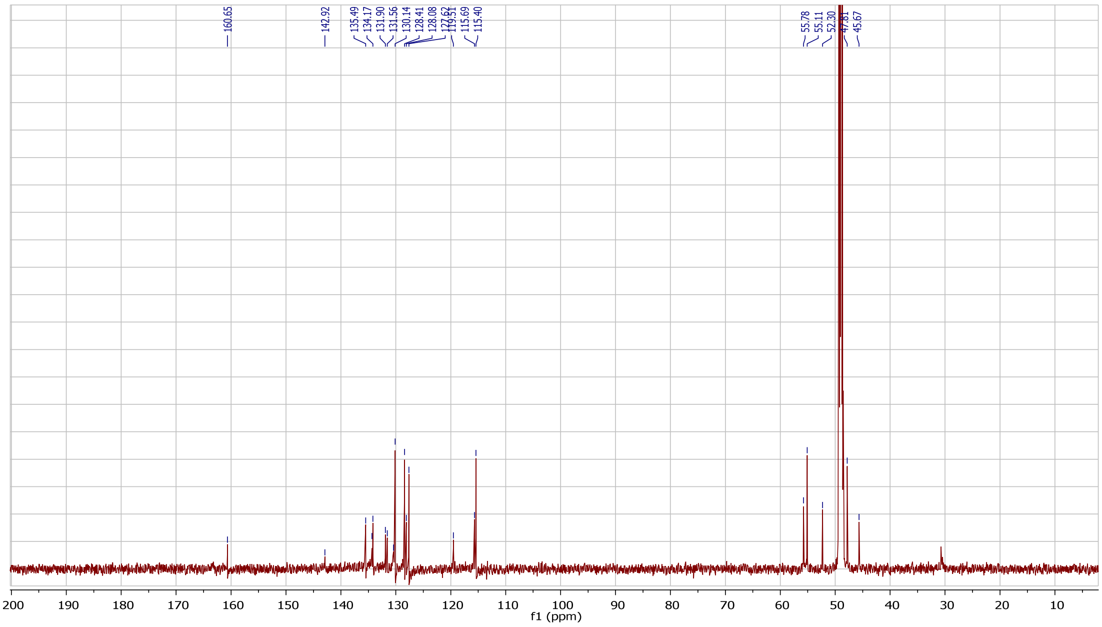

Figure S9. ^{1}H and ^{13}C NMR spectra of compound **12a** in methanol-d_4.

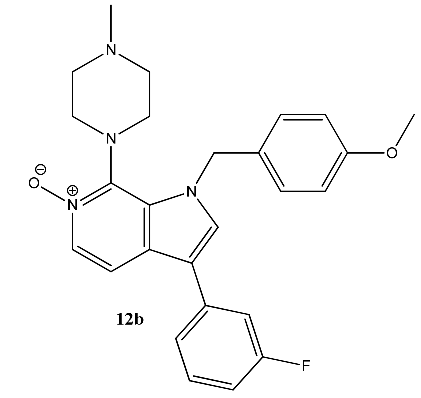

12b

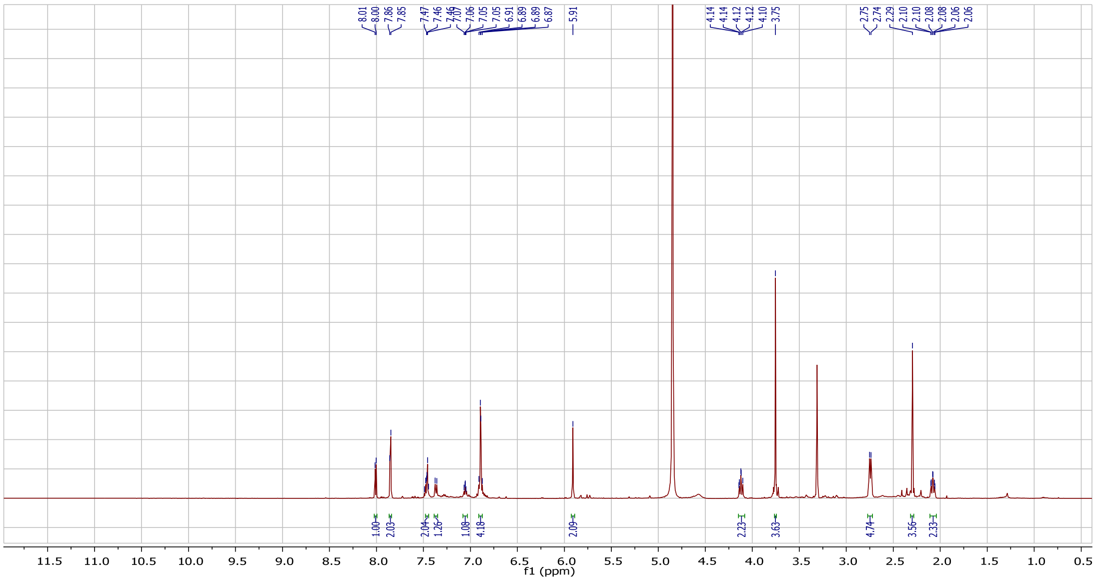

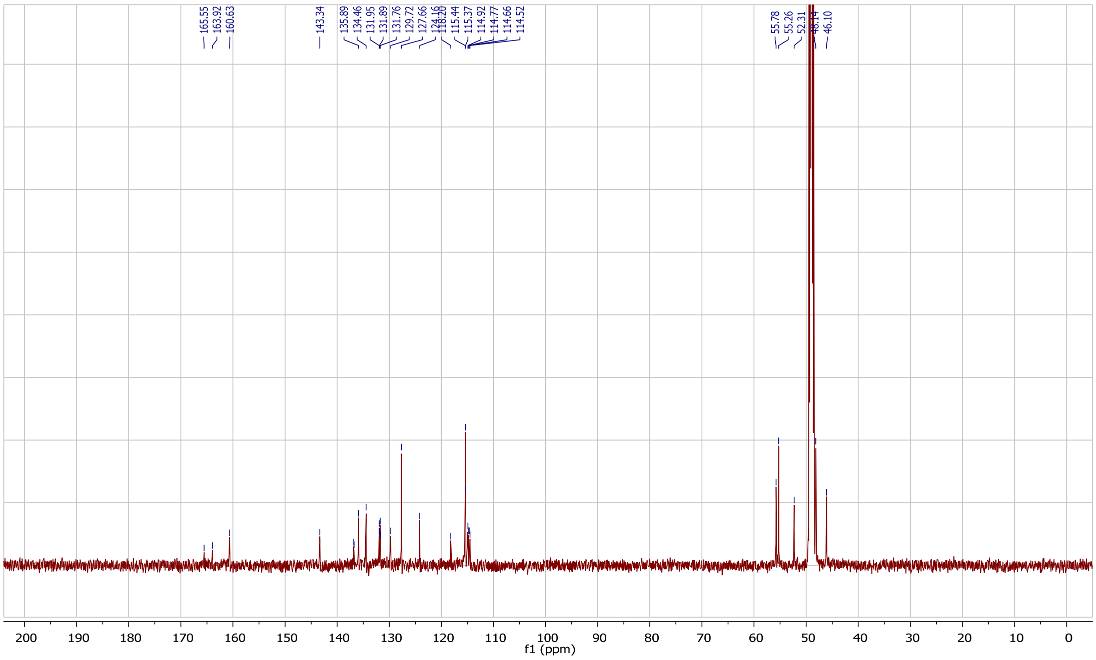

Figure S10. ^{1}H and ^{13}C NMR spectra of compound **12b** in methanol-d_4.

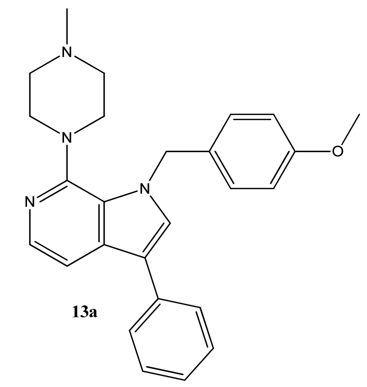

13a

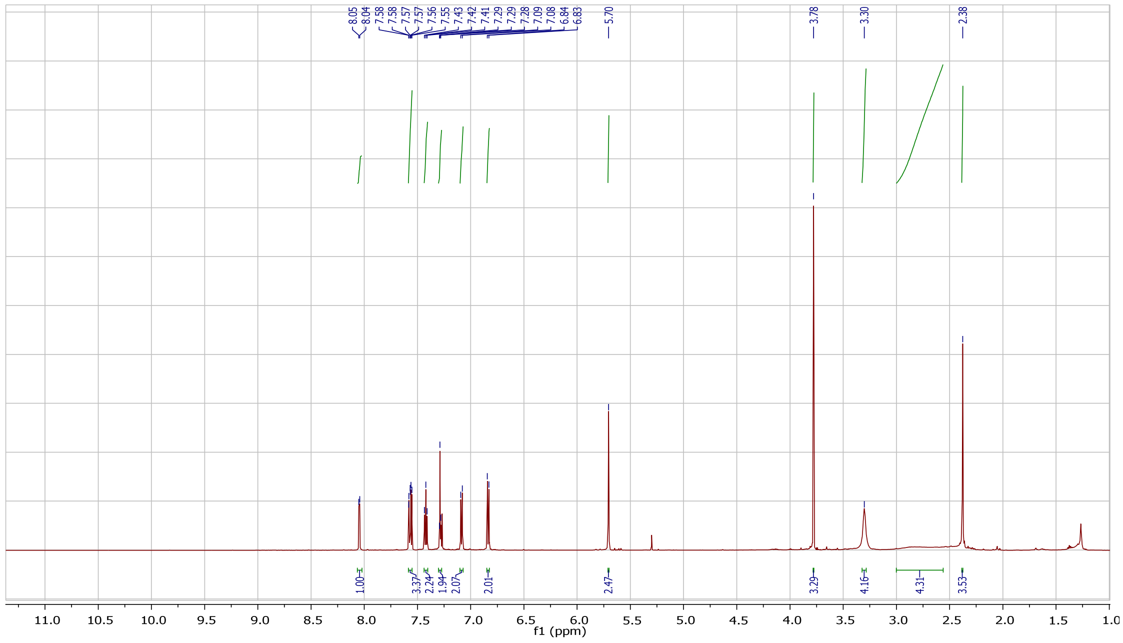

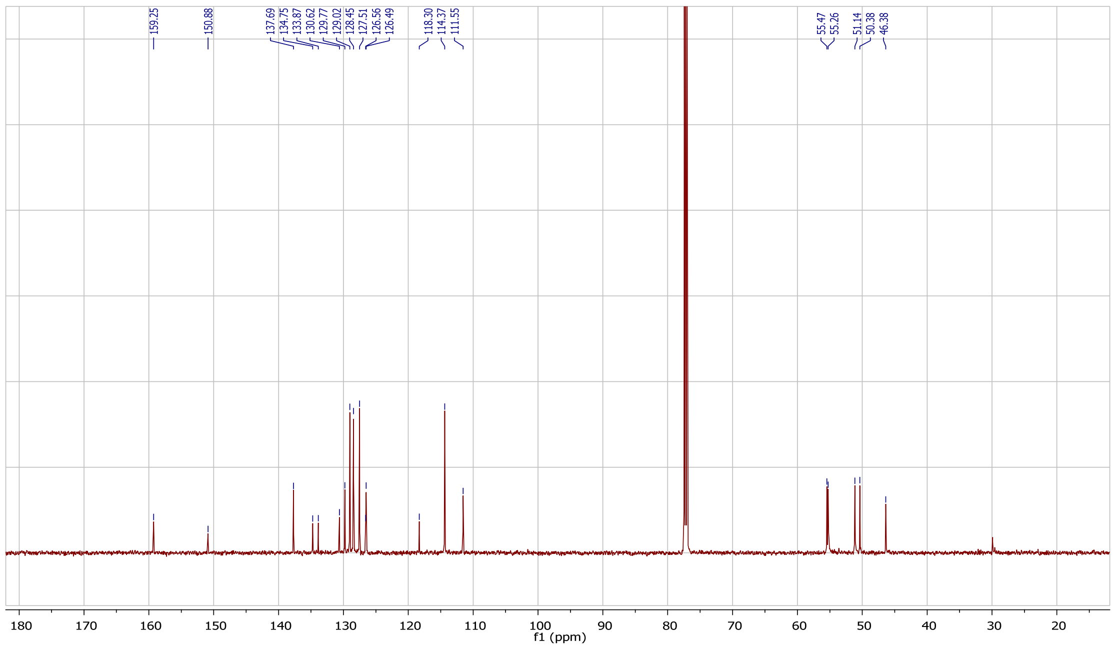

Figure S11. ^{1}H and ^{13}C NMR spectra of compound **13a** in $CDCl_3$.

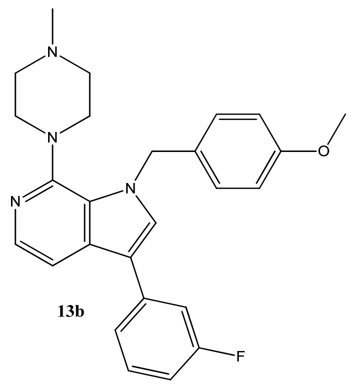

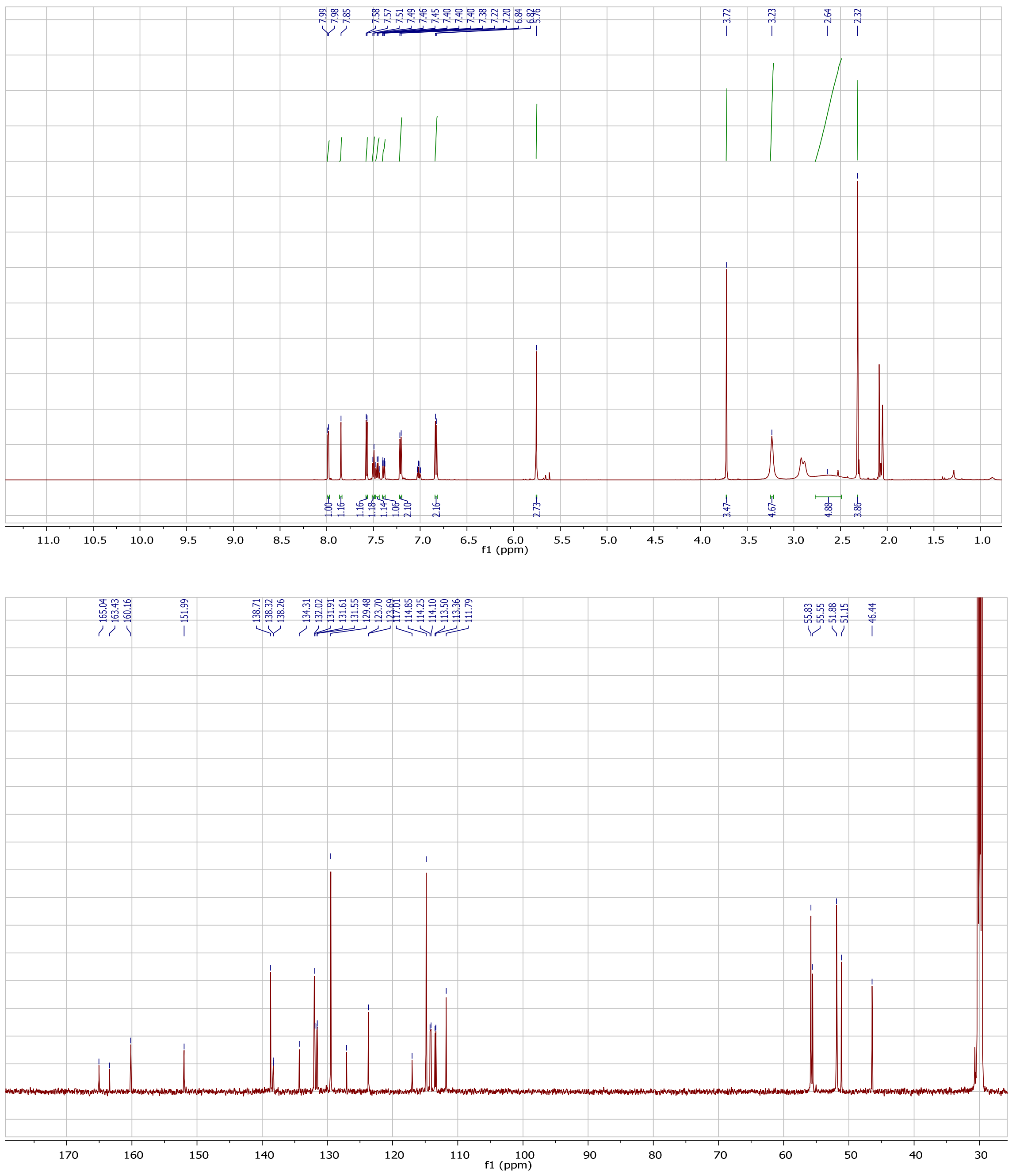

Figure S12. ^{1}H and ^{13}C NMR spectra of compound **13b** in acetone-d_6.

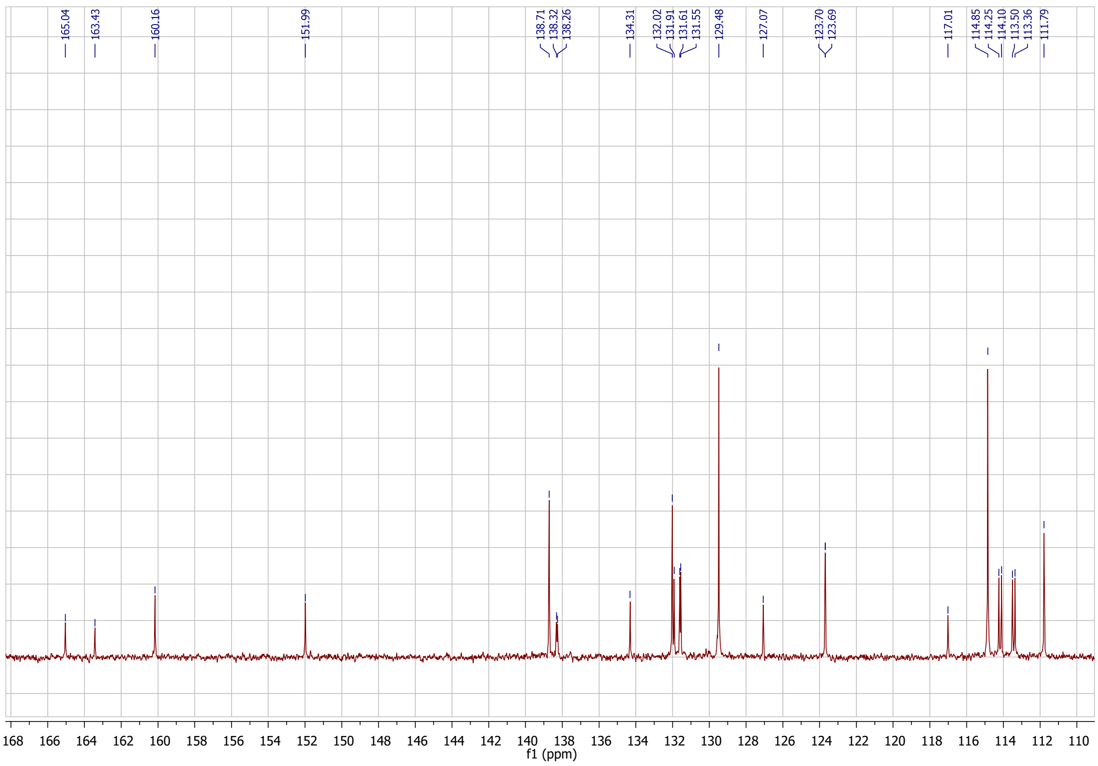

Figure S13. Zoom of the aromatic area of the ^{13}C NMR spectrum of compound **13b** in acetone-d_6.

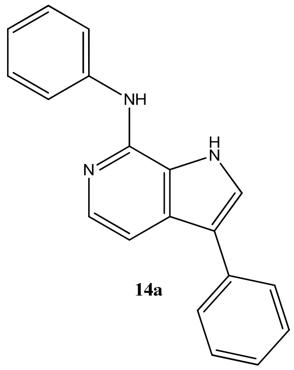

14a

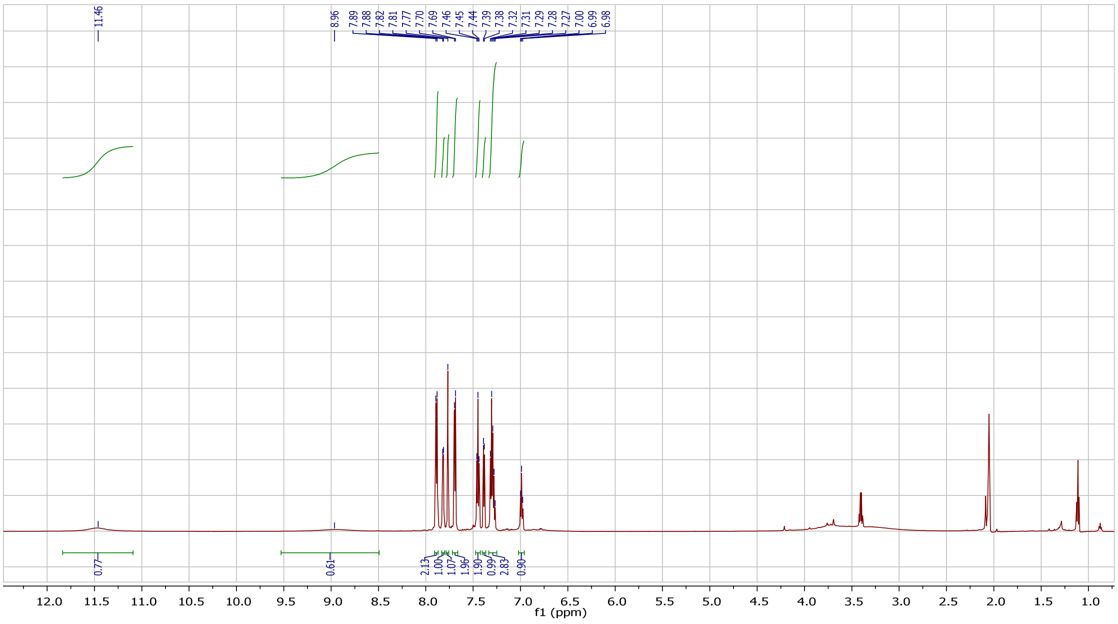

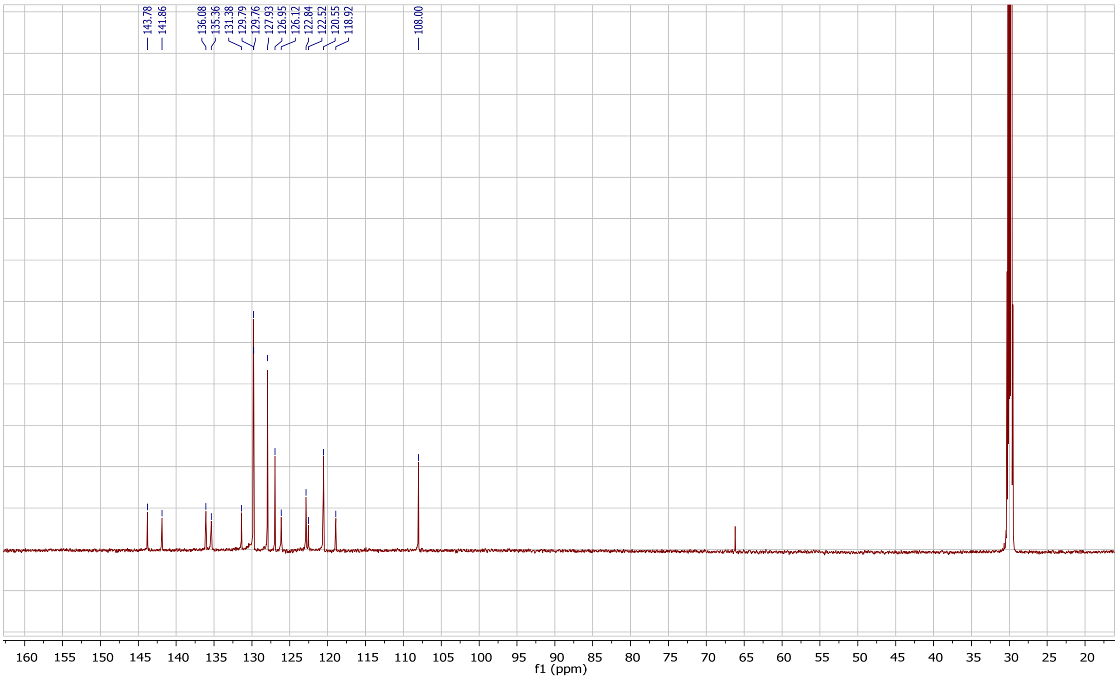

Figure S14. ^{1}H and ^{13}C NMR spectra of compound **14a** in acetone-*d*$_6$.

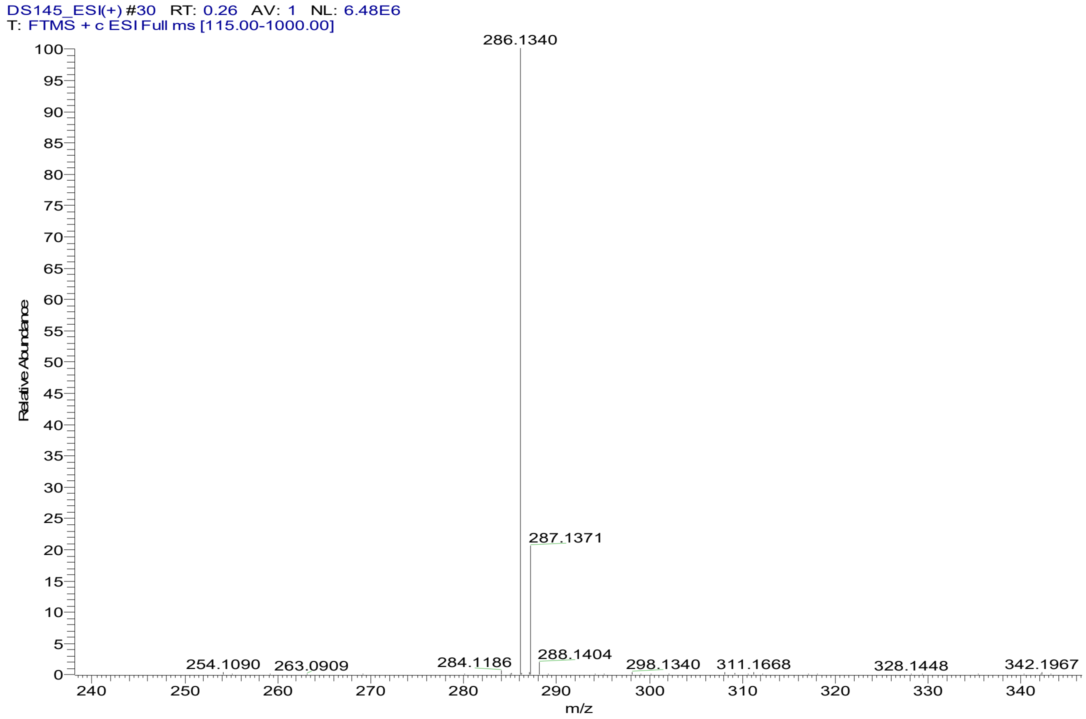

Figure S15. HRMS spectrum (ESI +) of compound **14a.**

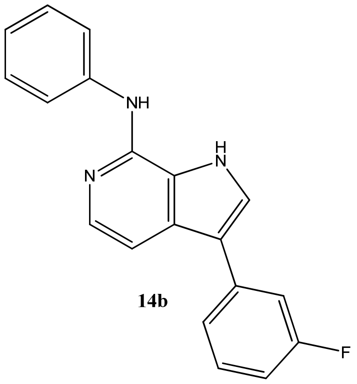

14b

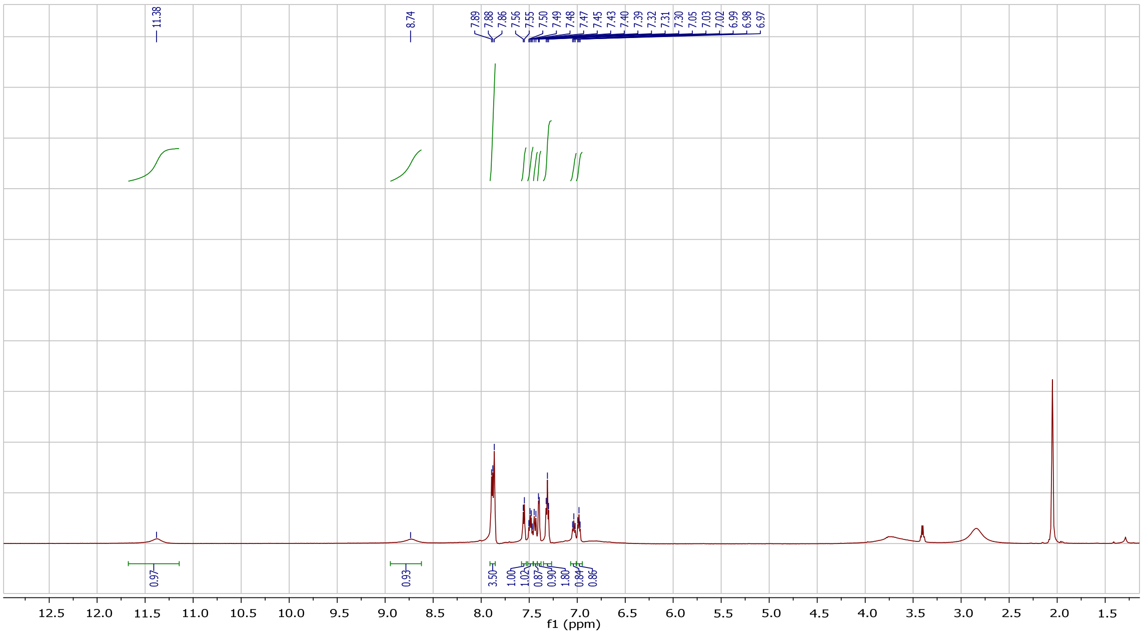

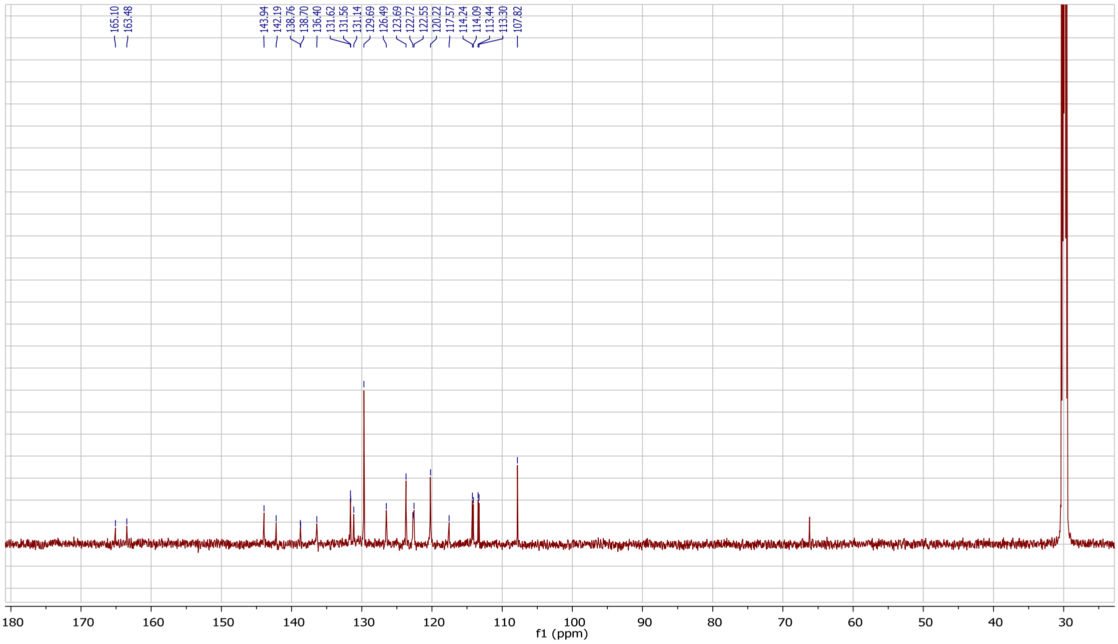

Figure S16. ^{1}H and ^{13}C NMR spectra of compound **14b** in acetone-d_6.

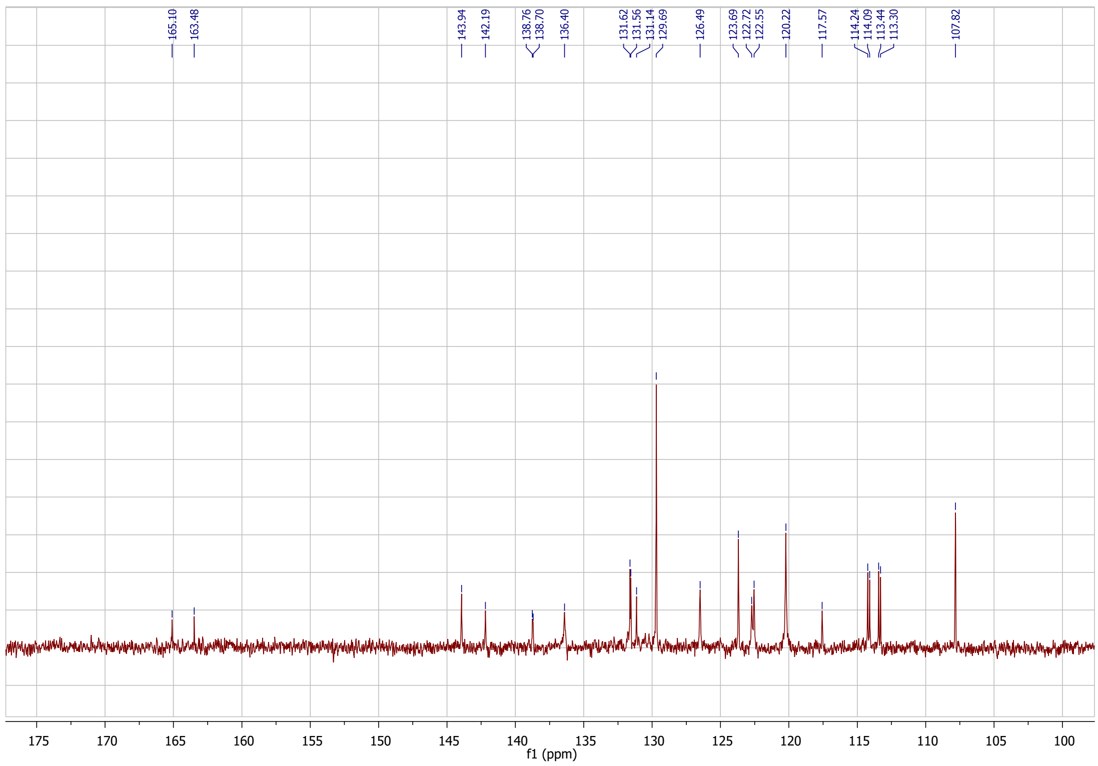

Figure S17. Zoom of the aromatic area of the ^{13}C NMR spectrum of compound **14b** in acetone-d_6.

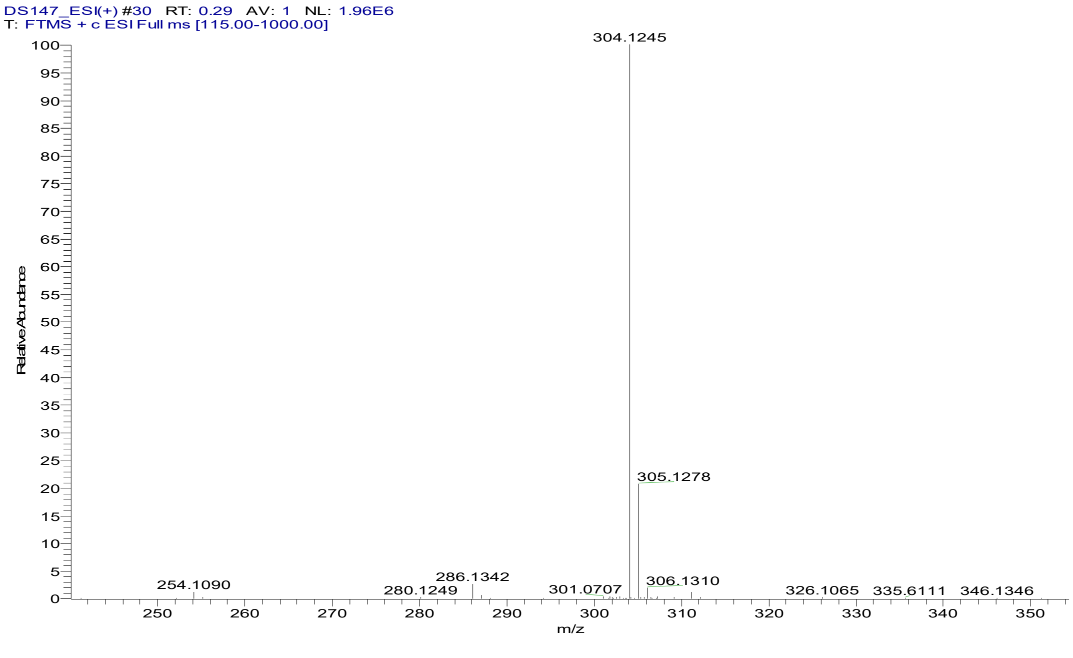

Figure S18. HRMS spectrum (ESI +) of compound **14b.**

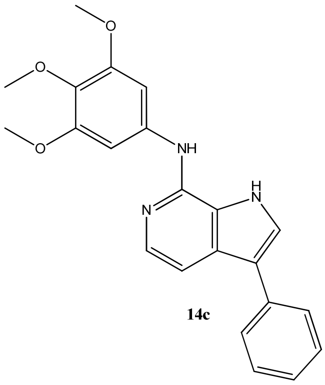

14c

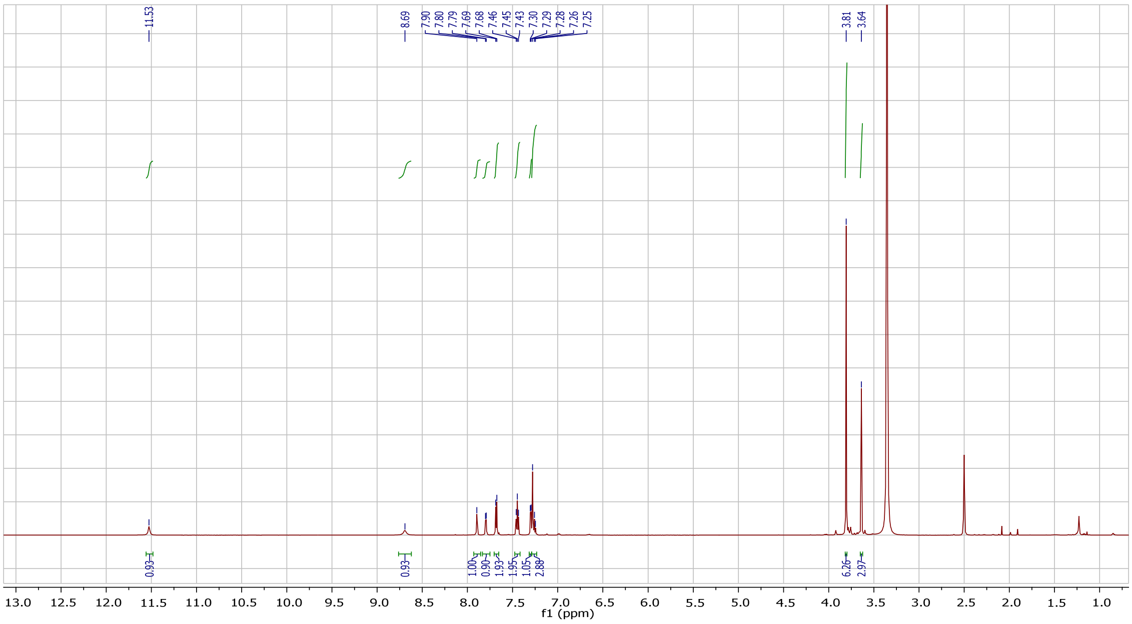

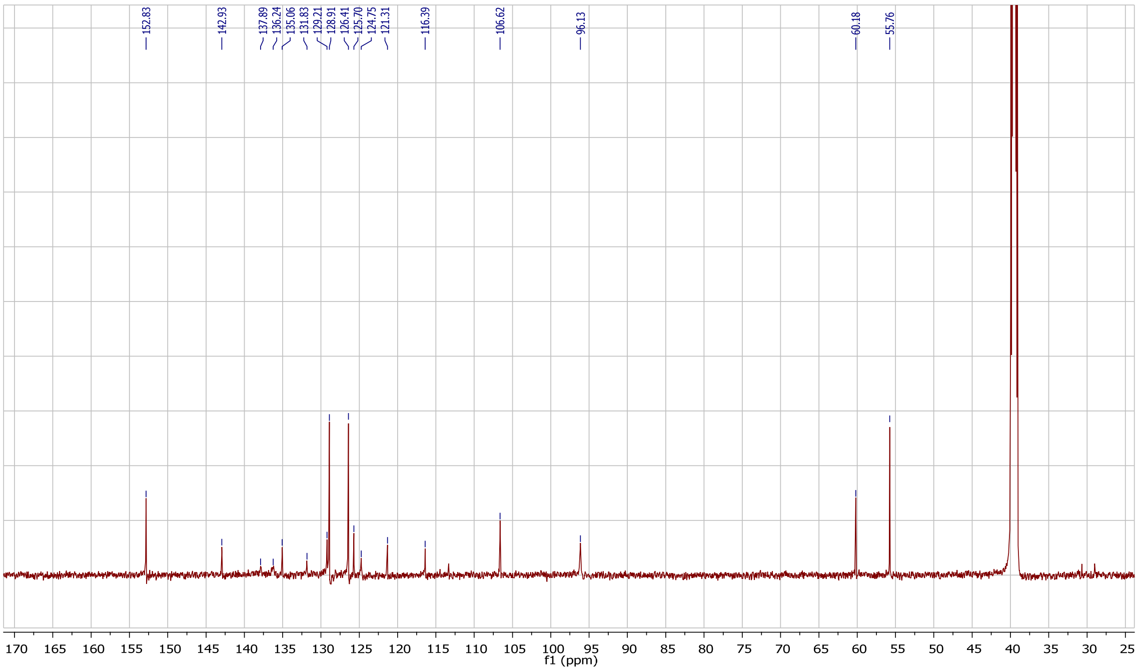

Figure S19. ^{1}H and ^{13}C NMR spectra of compound **14c** in DMSO-d_6.

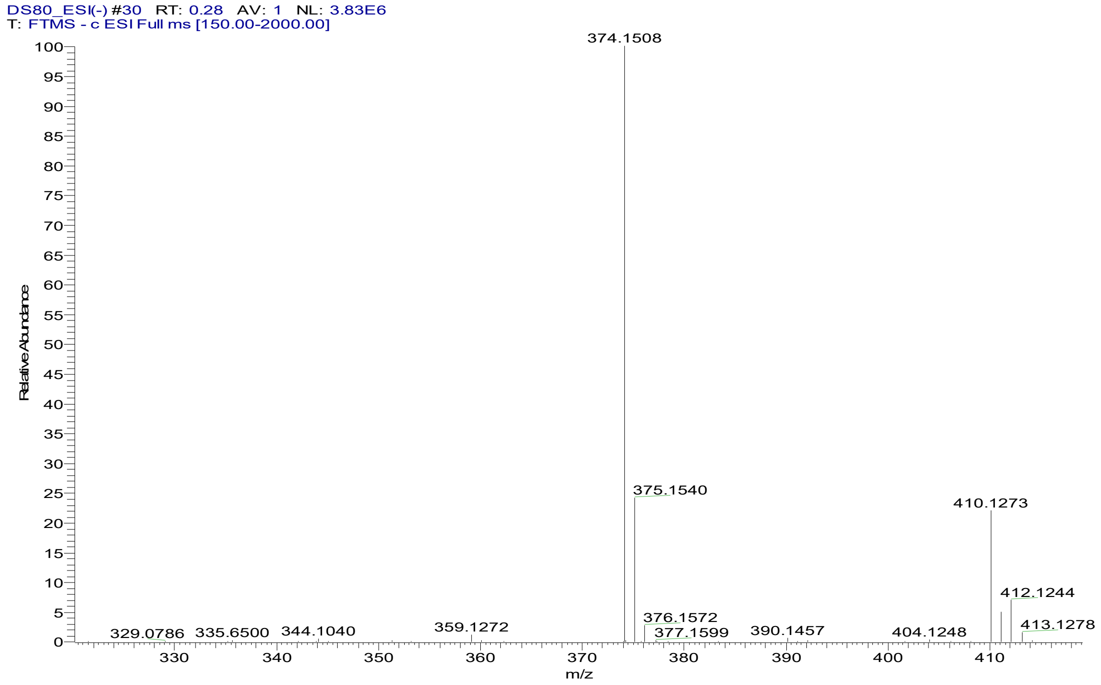

Figure S20. HRMS spectrum (ESI -) of compound **14c.**

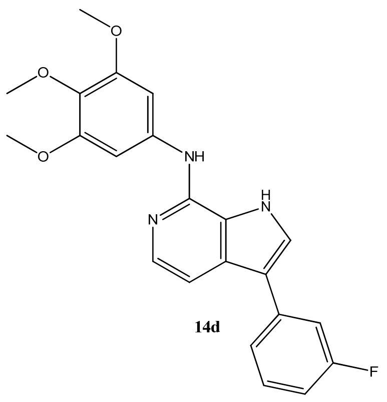

14d

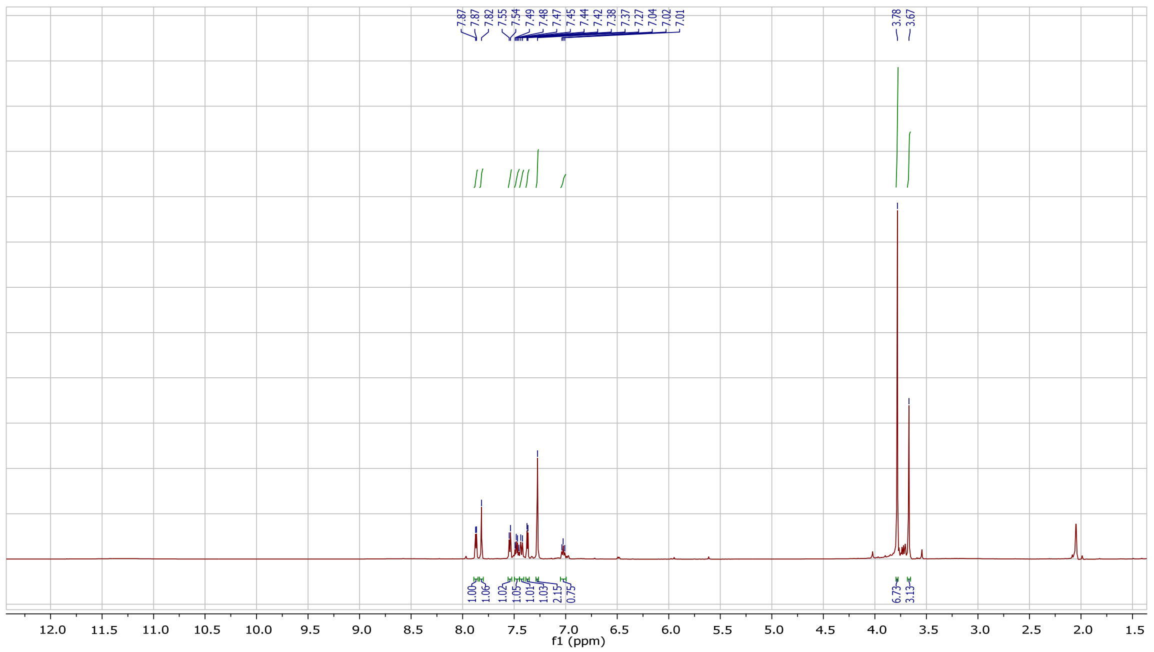

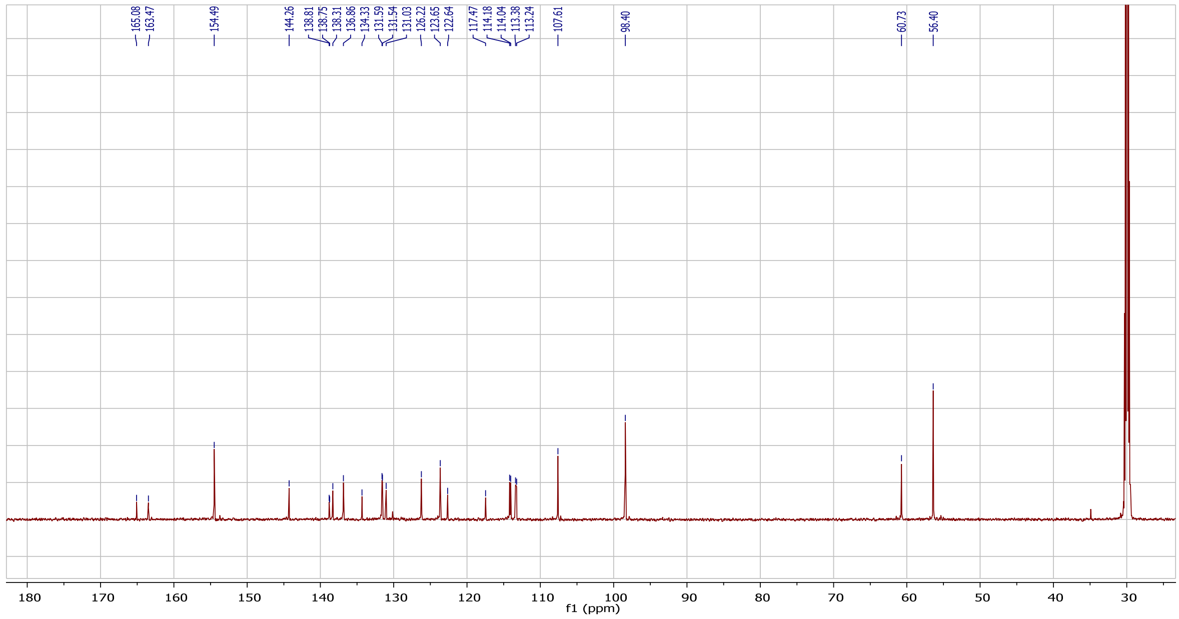

Figure S21. ^{1}H and ^{13}C NMR spectra of compound **14d** in acetone-d_6.

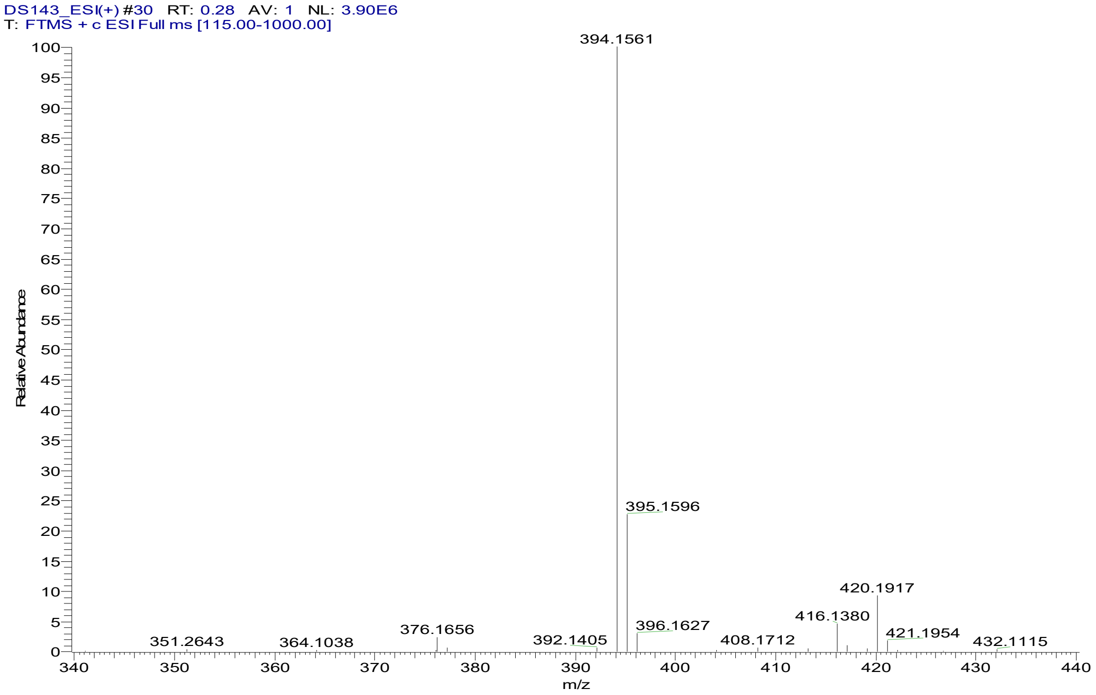

Figure S22. HRMS spectrum (ESI +) of compound **14d.**

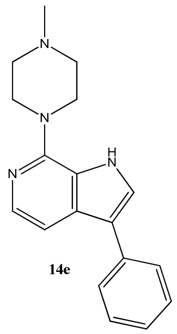

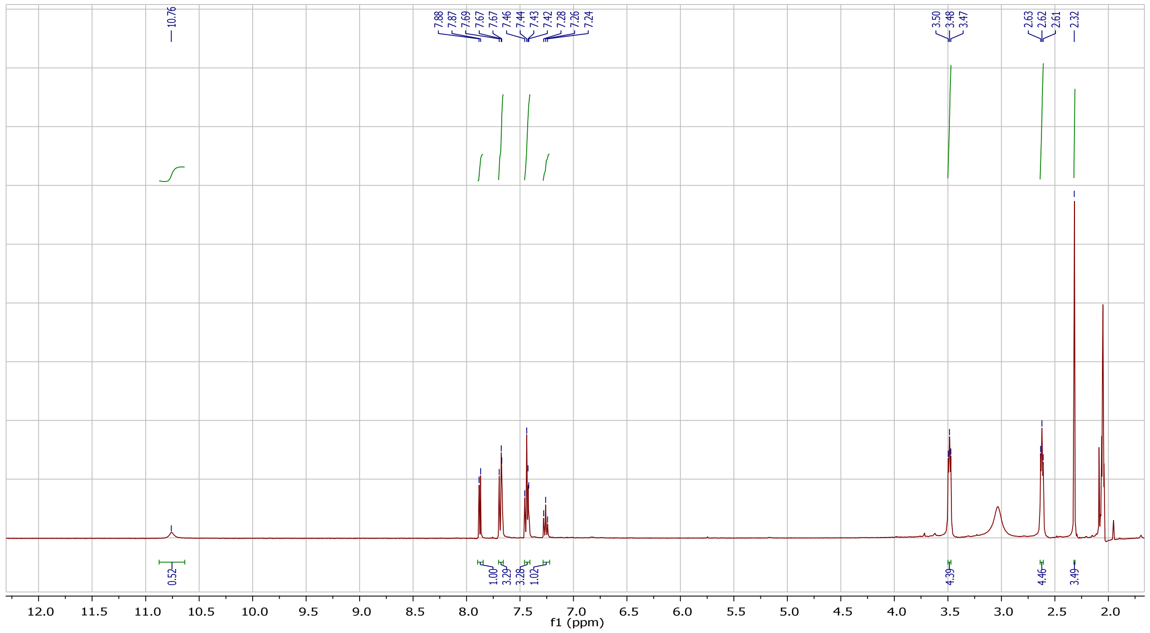

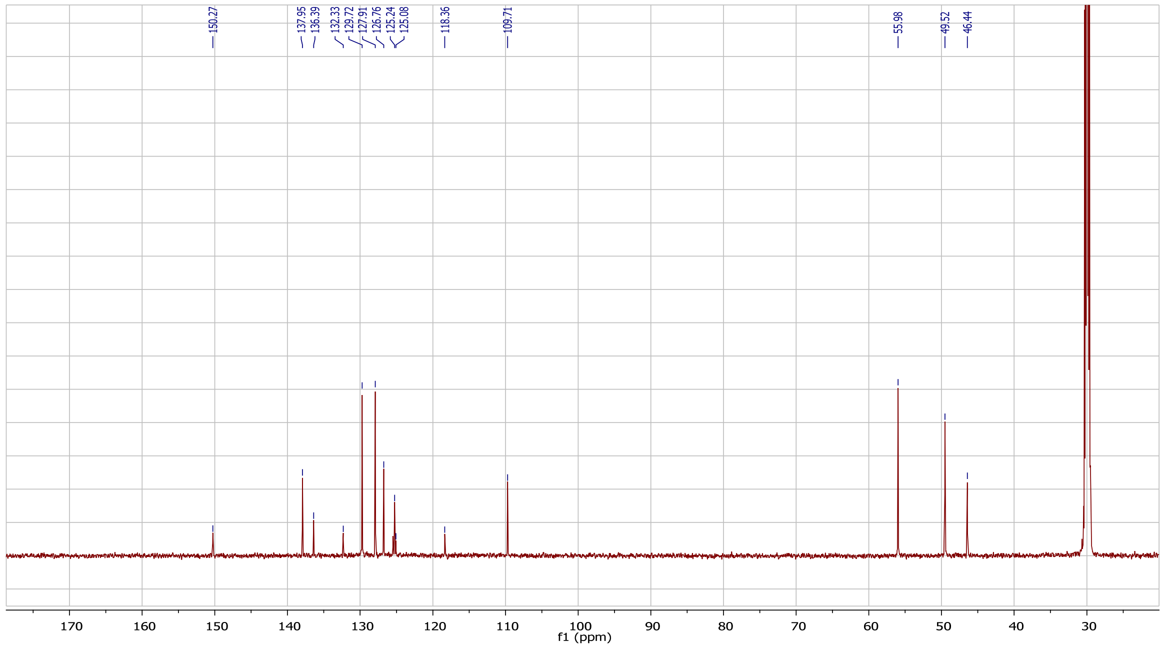

Figure S23. ^{1}H and ^{13}C NMR spectra of compound **14e** in acetone-d_6.

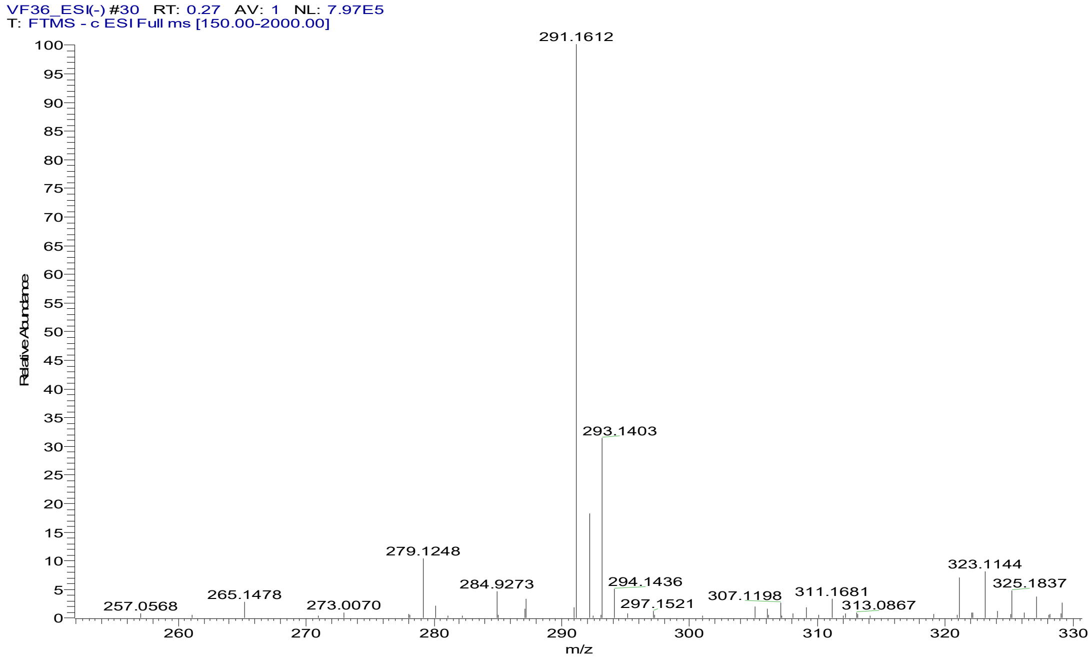

Figure S24. HRMS spectrum (ESI -) of compound **14e.**

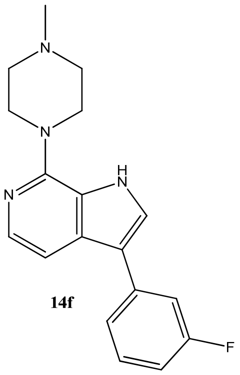

14f

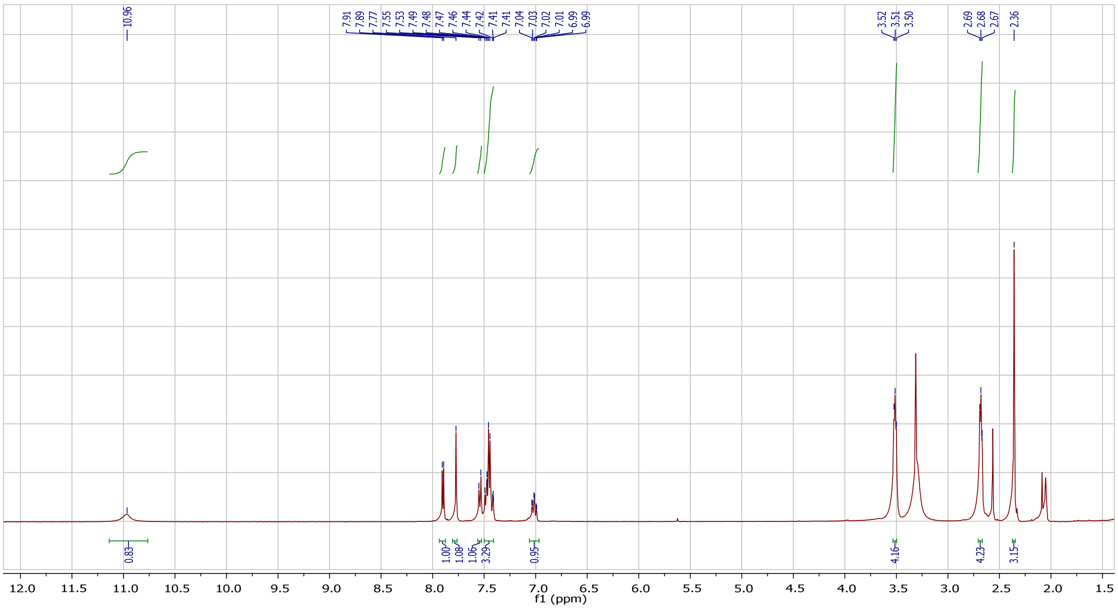

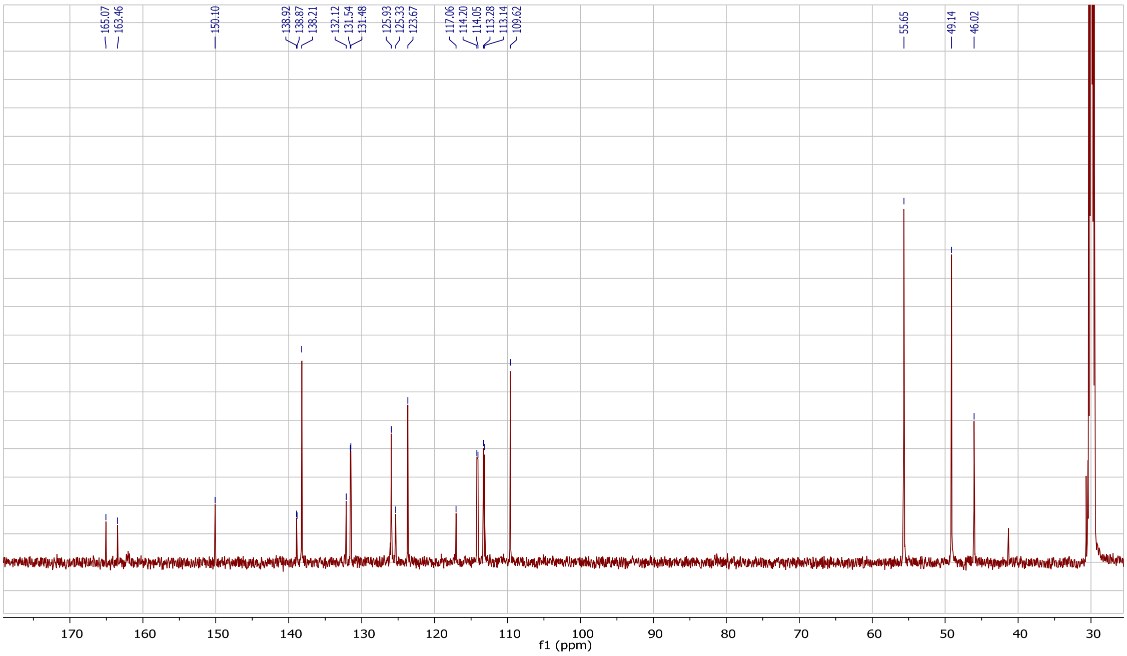

Figure S25. ^{1}H and ^{13}C NMR spectra of compound **14f** in acetone-d_6.

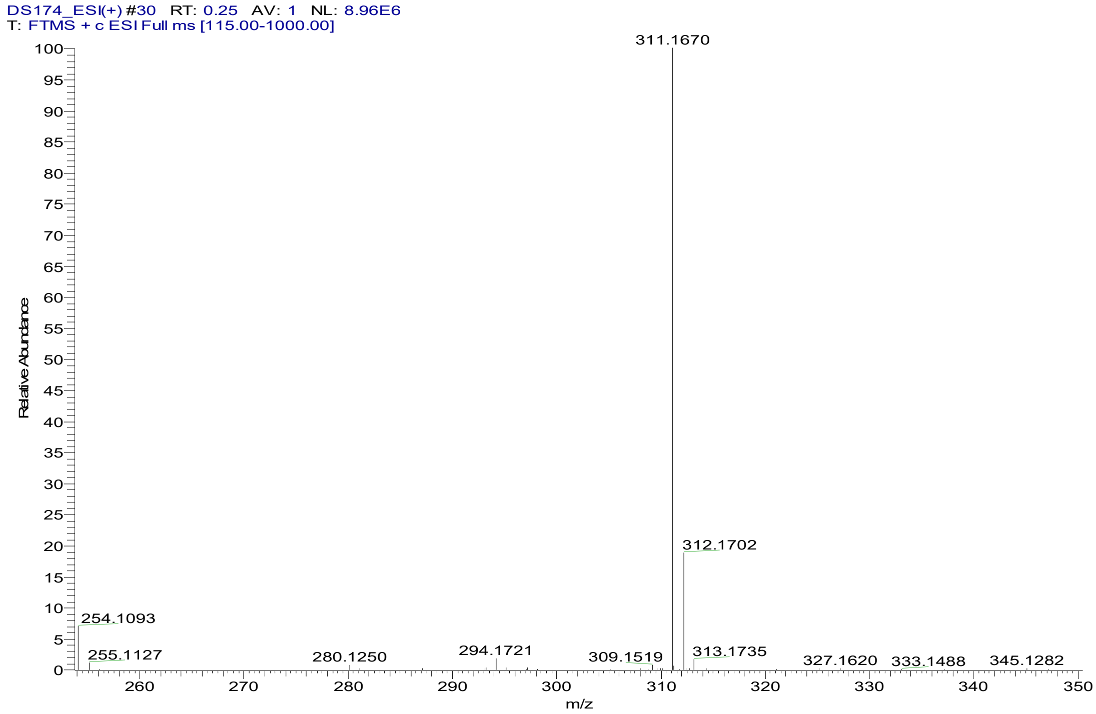

Figure S26. HRMS spectrum (ESI +) of compound **14f.**

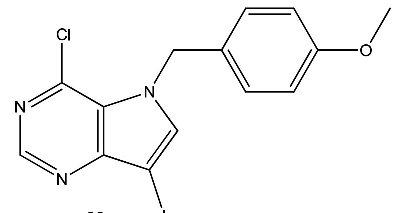

22

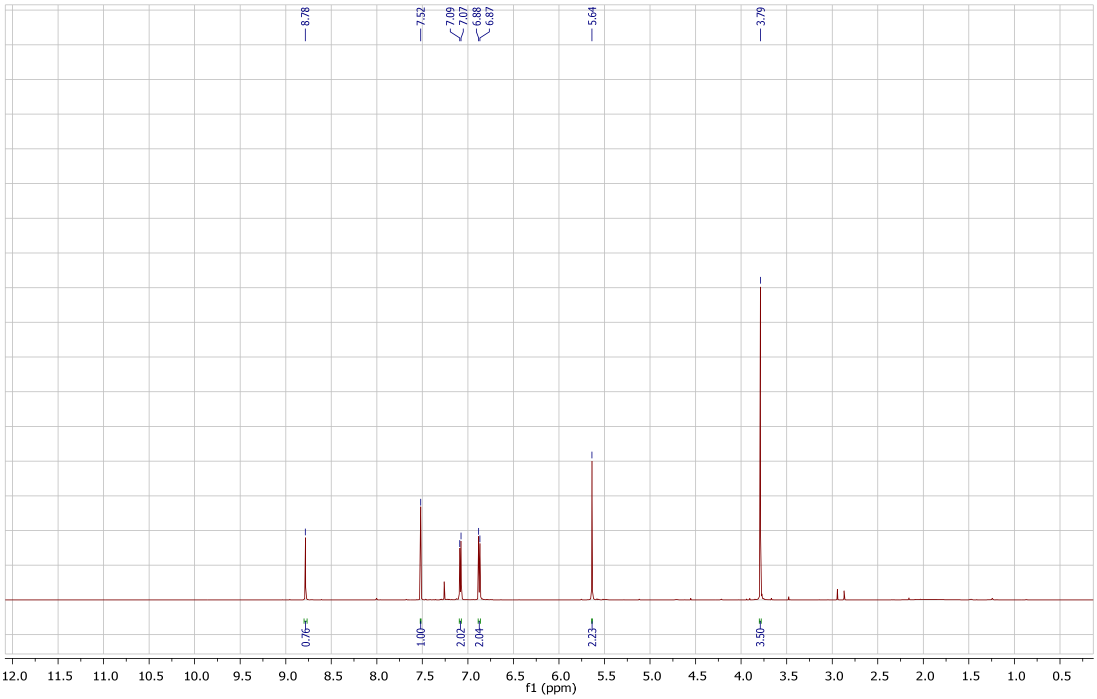

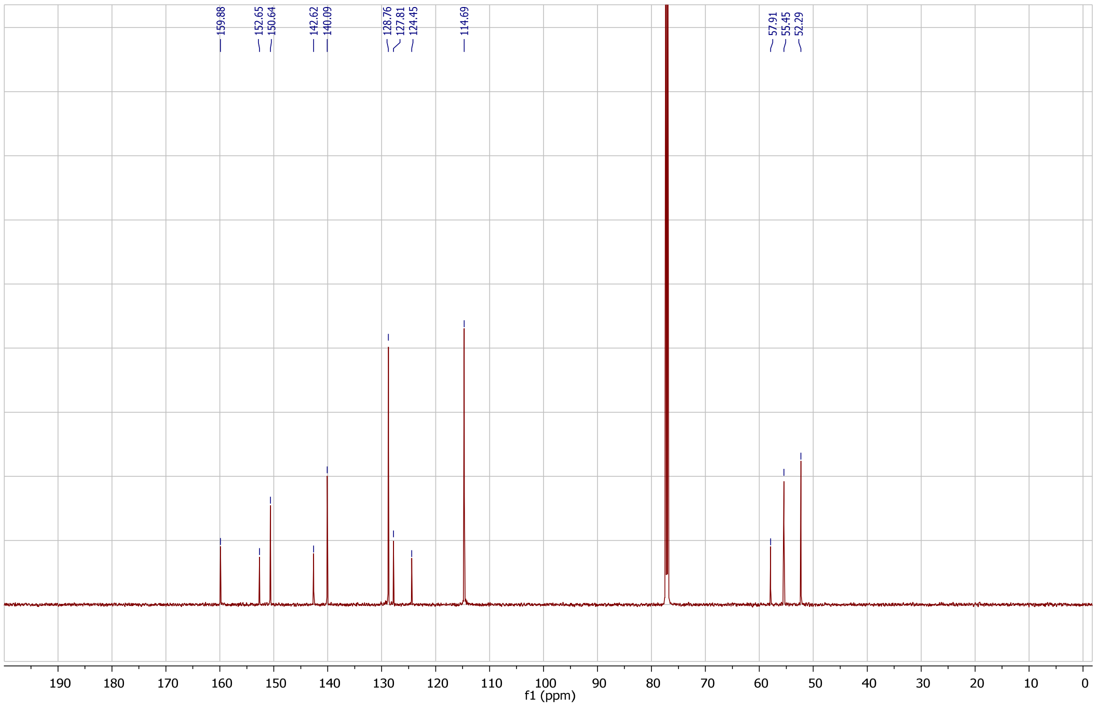

Figure S27. ^{1}H and ^{13}C NMR spectra of compound **22** in $CDCl_3$.

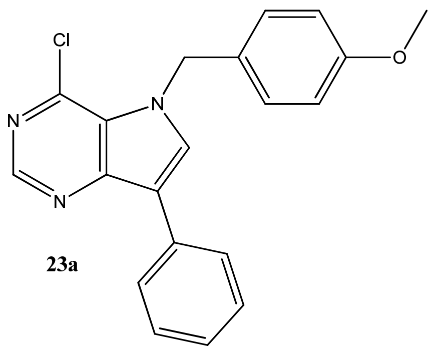

23a

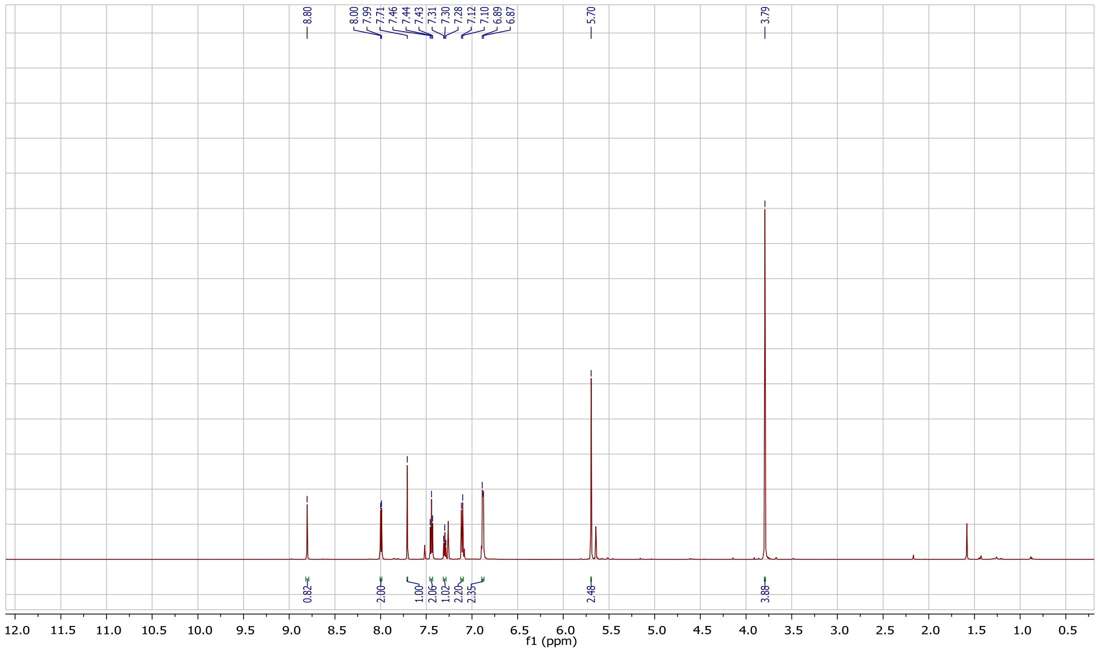

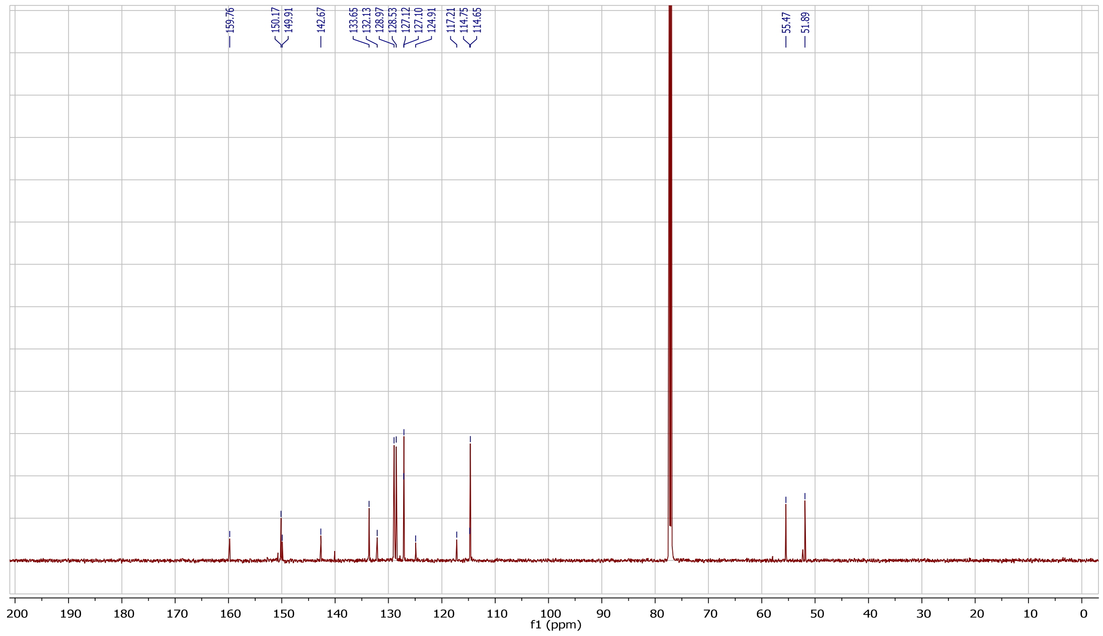

Figure S28. ^{1}H and ^{13}C NMR spectra of compound **23a** in $CDCl_3$.

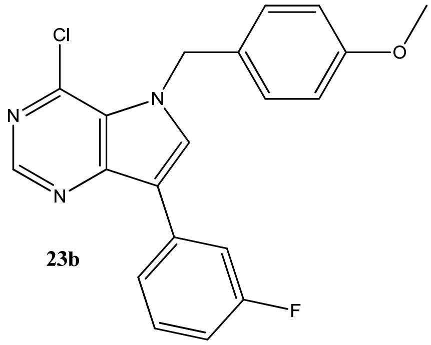

23b

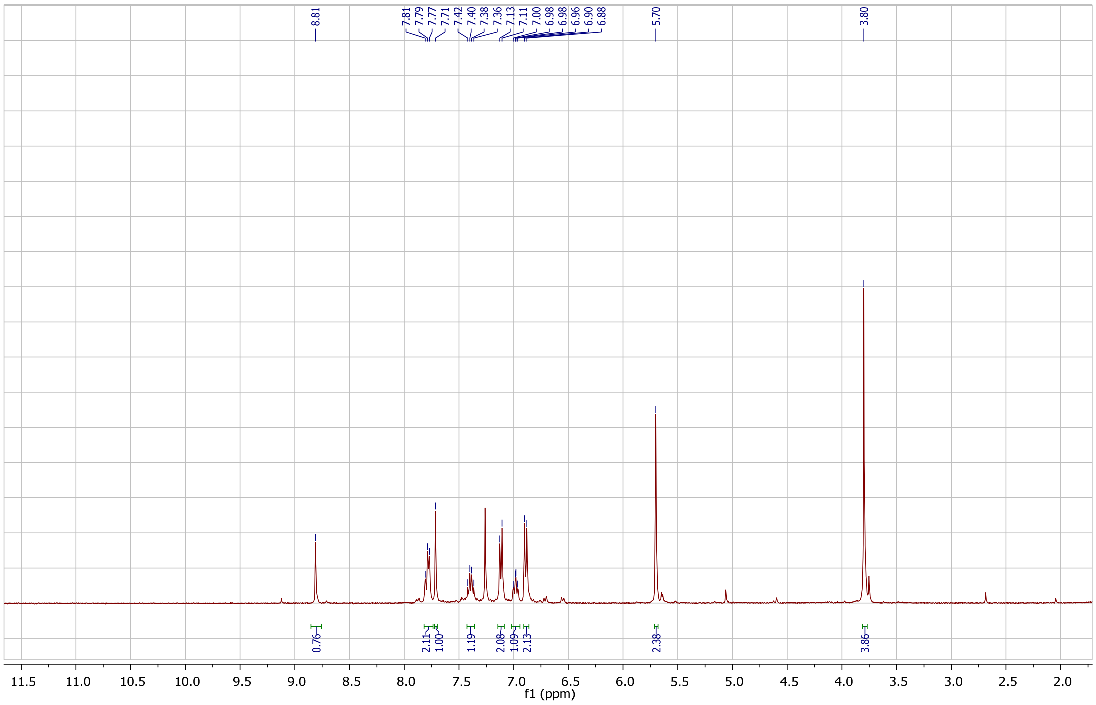

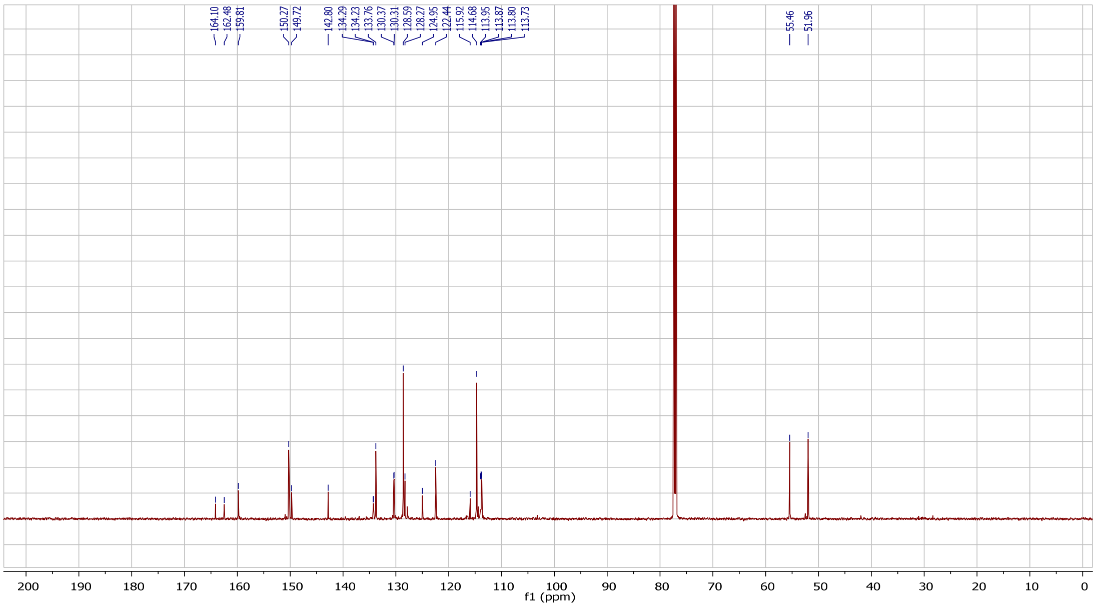

Figure S29. ^{1}H and ^{13}C NMR spectra of compound **23b** in $CDCl_3$.

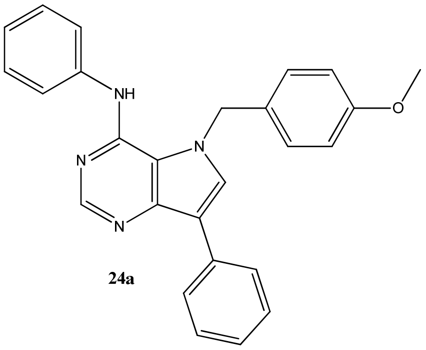

24a

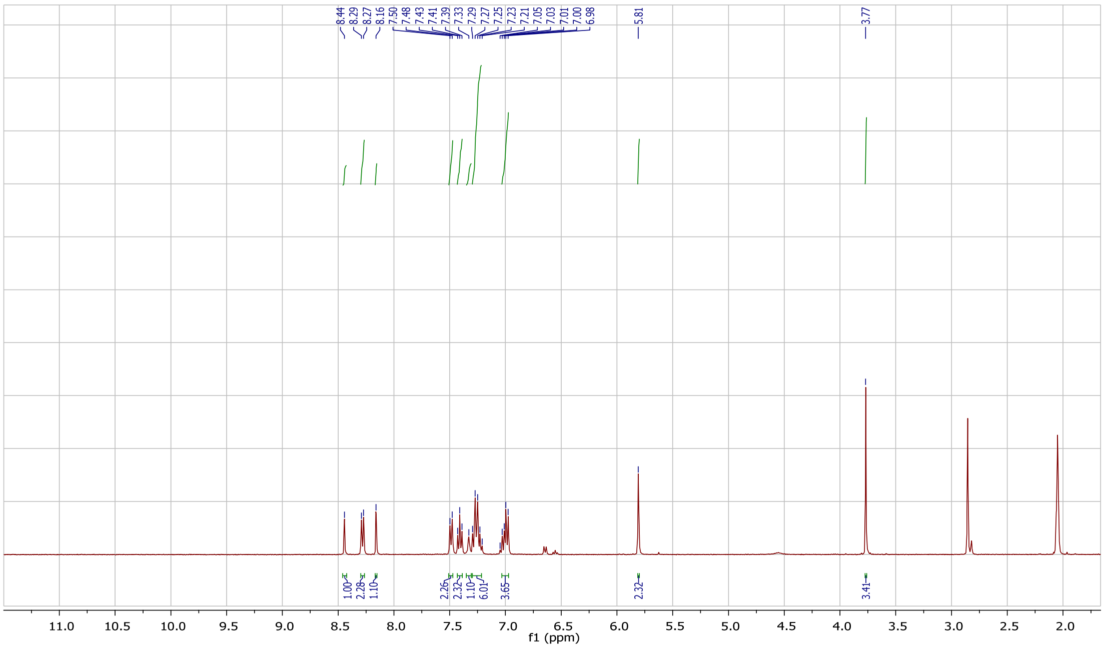

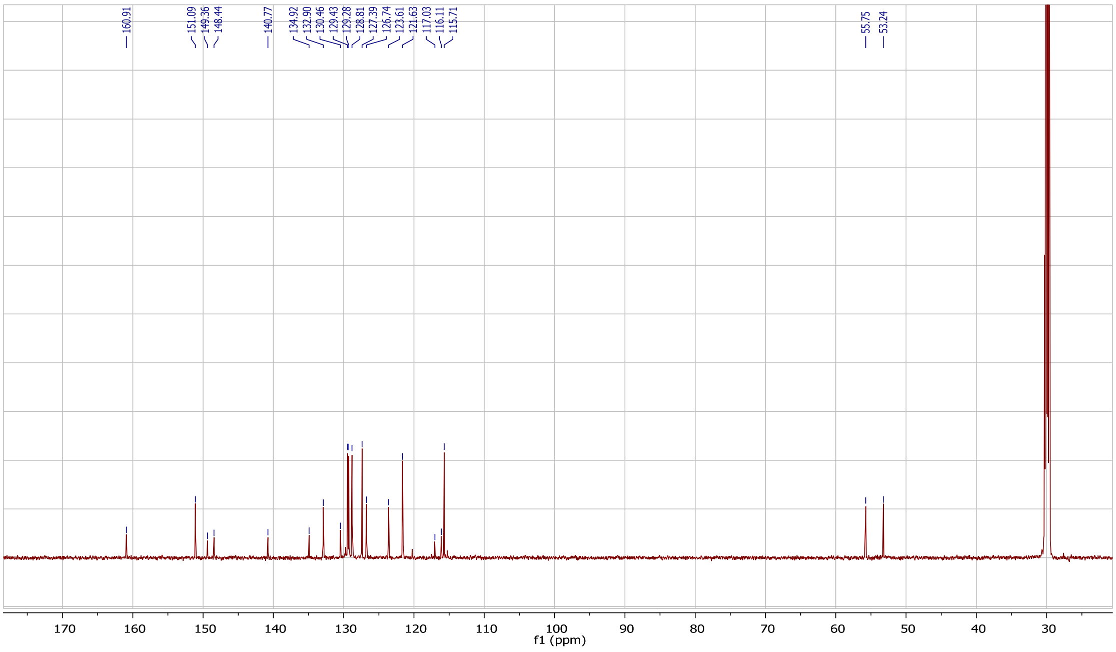

Figure S30. ^{1}H and ^{13}C NMR spectra of compound **24a** in acetone-d_6.

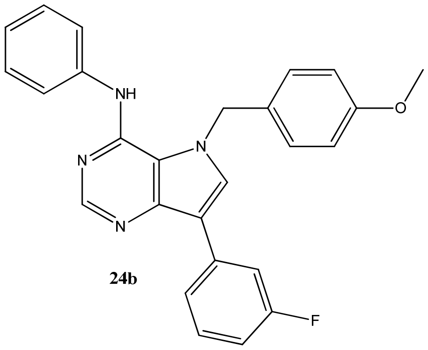

24b

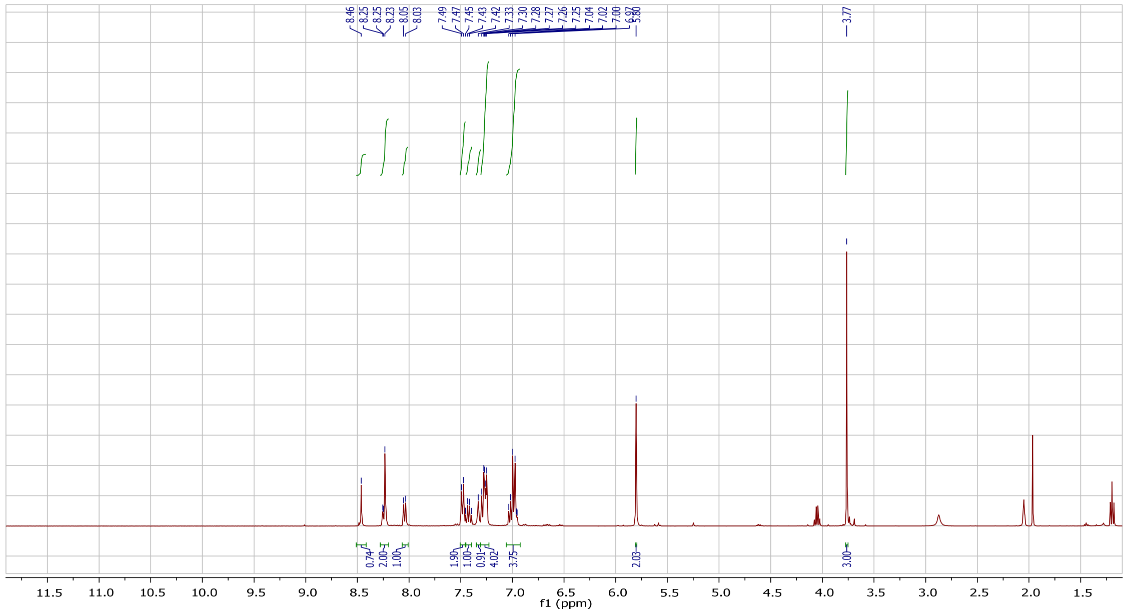

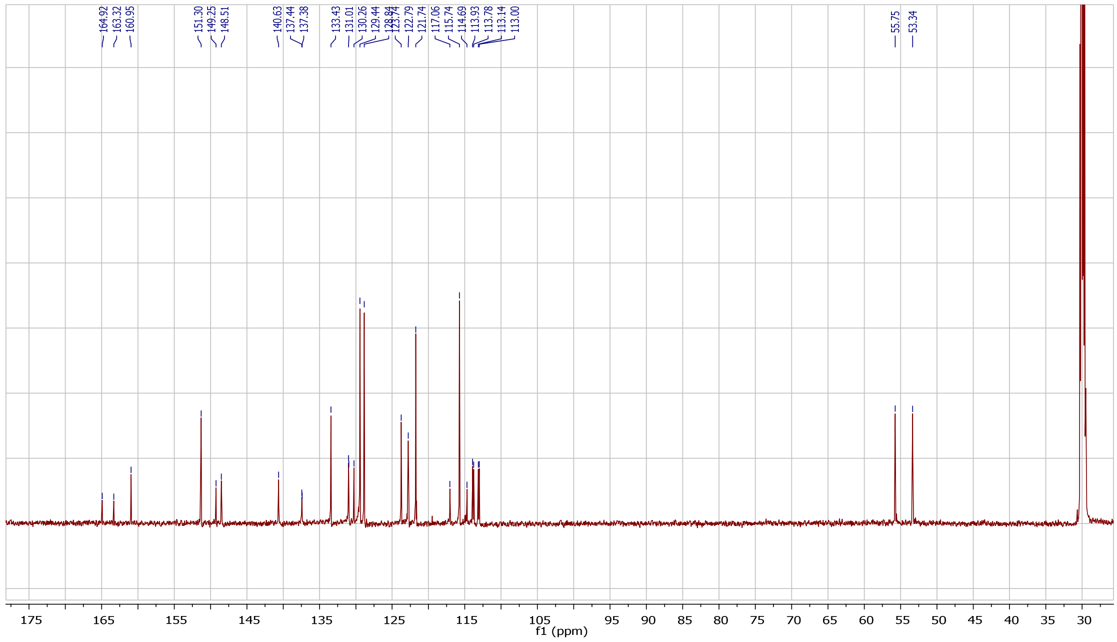

Figure S31. ^{1}H and ^{13}C NMR spectra of compound **24b** in acetone-d_6.

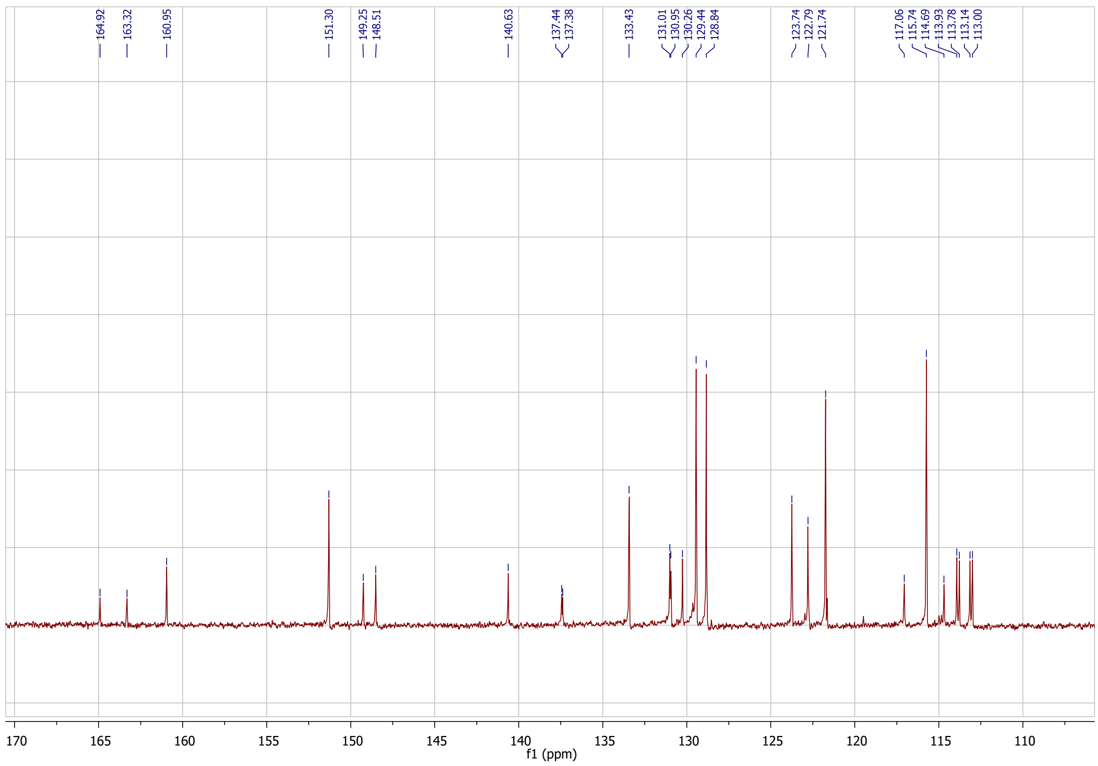

Figure S32. Zoom of the aromatic area of the ^{13}C NMR spectrum of compound **24b** in acetone-d_6.

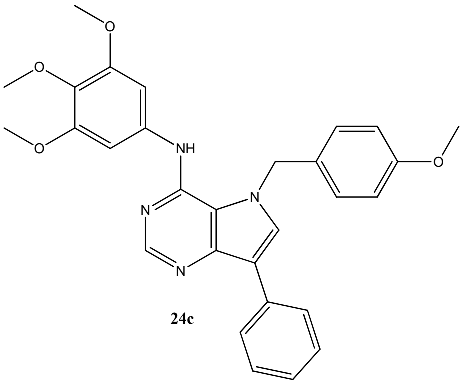

24c

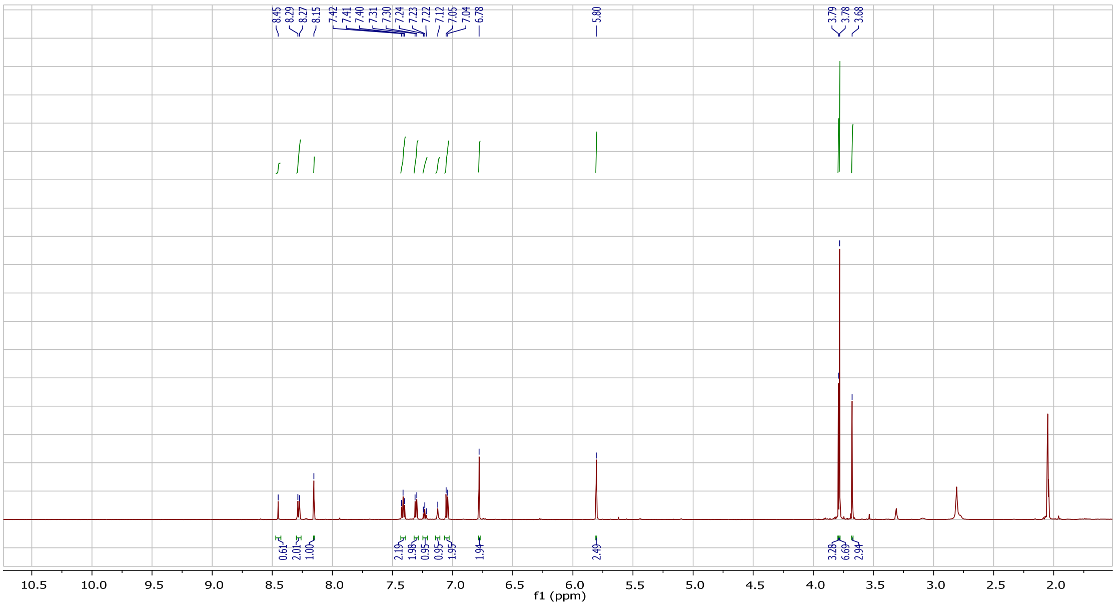

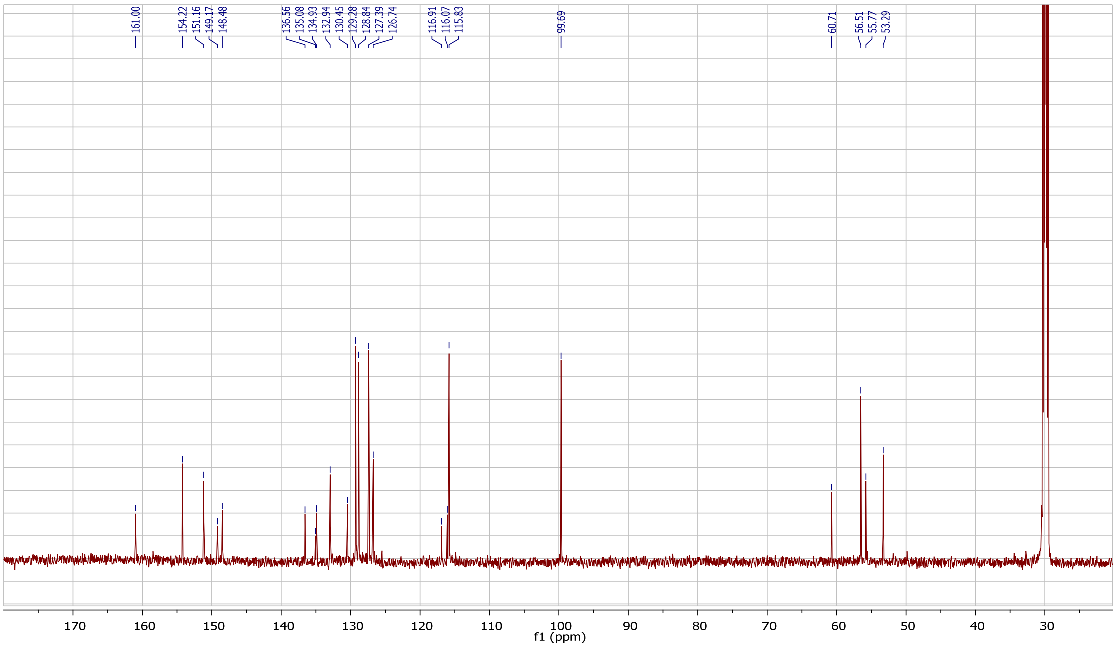

Figure S33. ^{1}H and ^{13}C NMR spectra of compound **24c** in acetone-d_6.

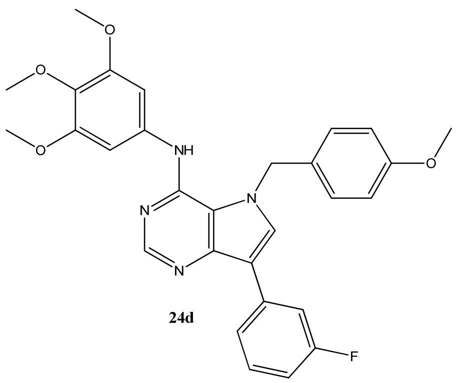

24d

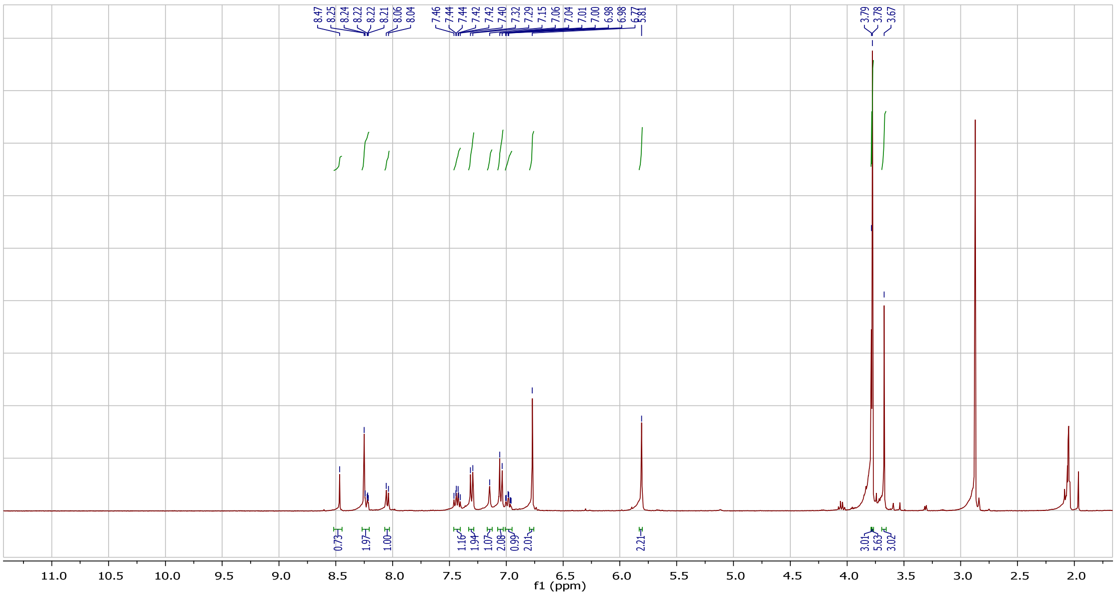

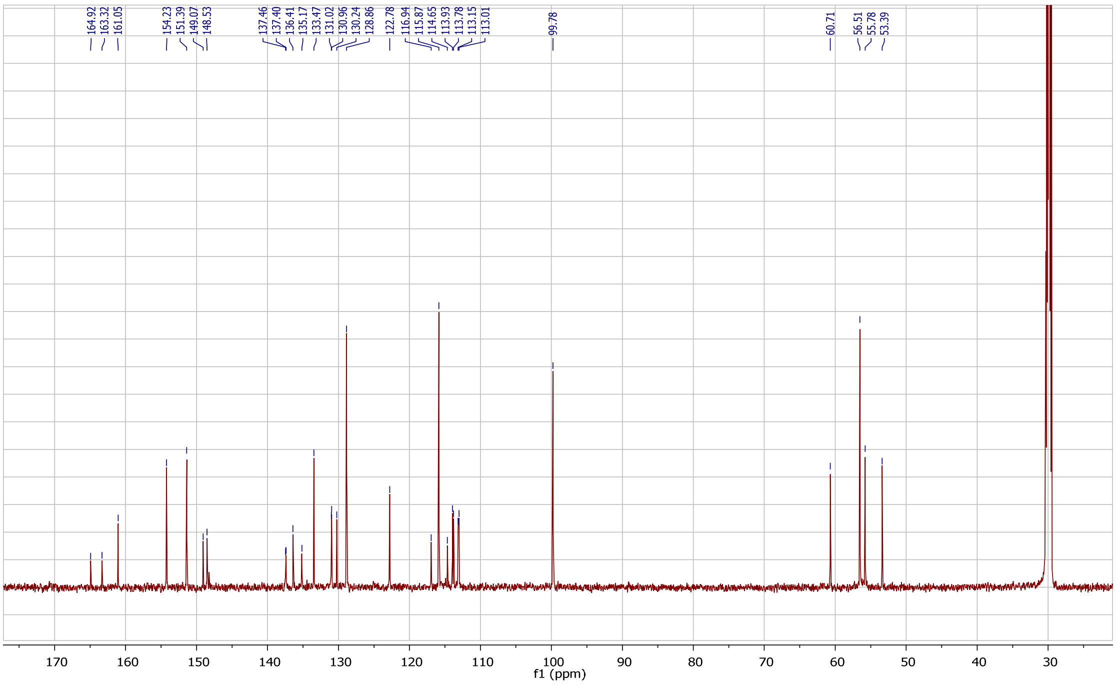

Figure S34. ^{1}H and ^{13}C NMR spectra of compound **24d** in acetone-d_6.

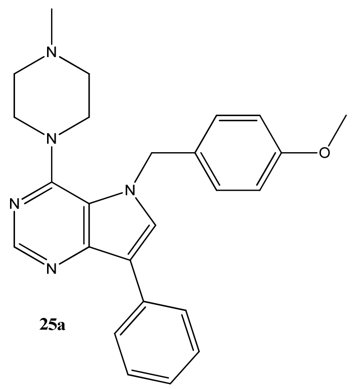

25a

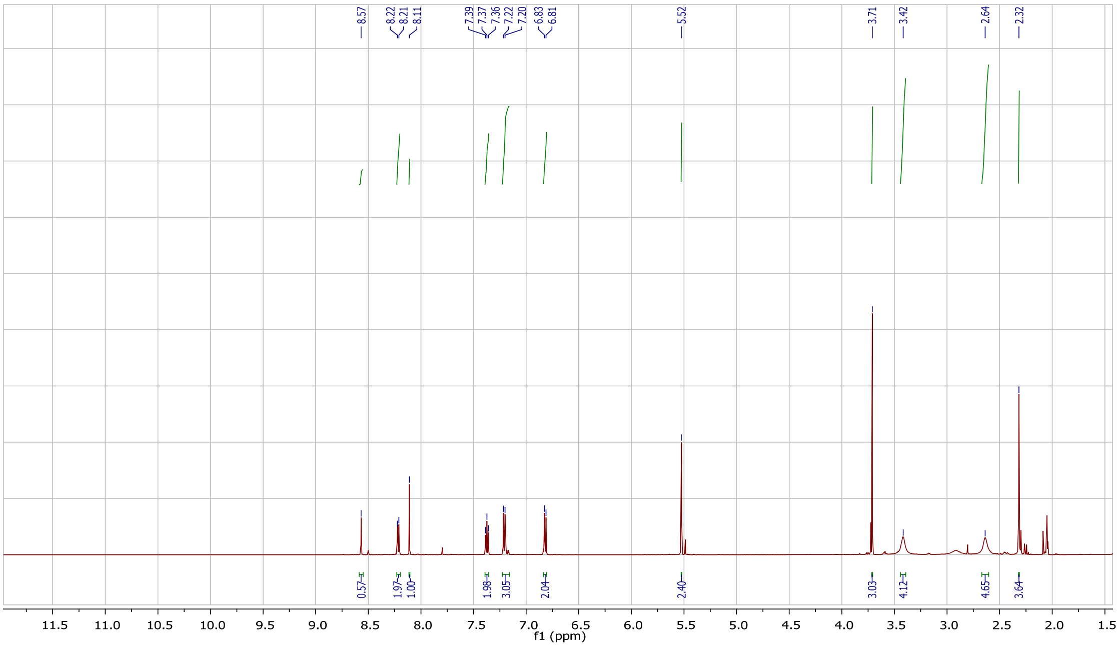

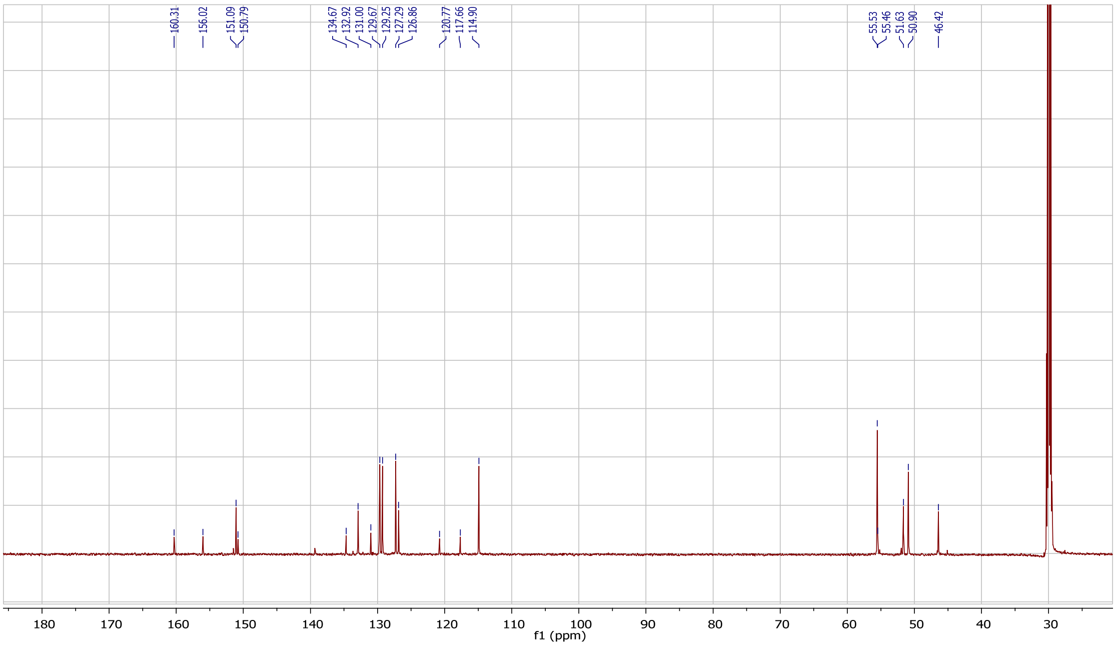

Figure S35. ^{1}H and ^{13}C NMR spectra of compound **25a** in acetone-d_6.

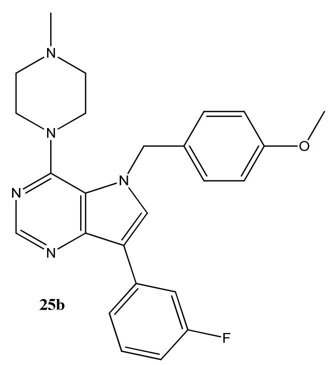

25b

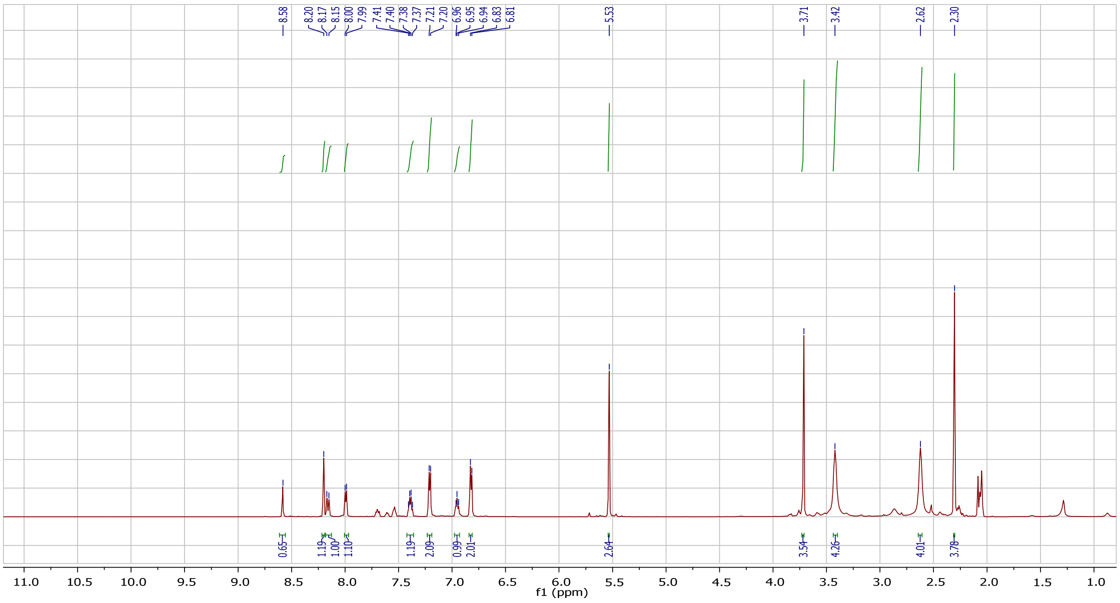

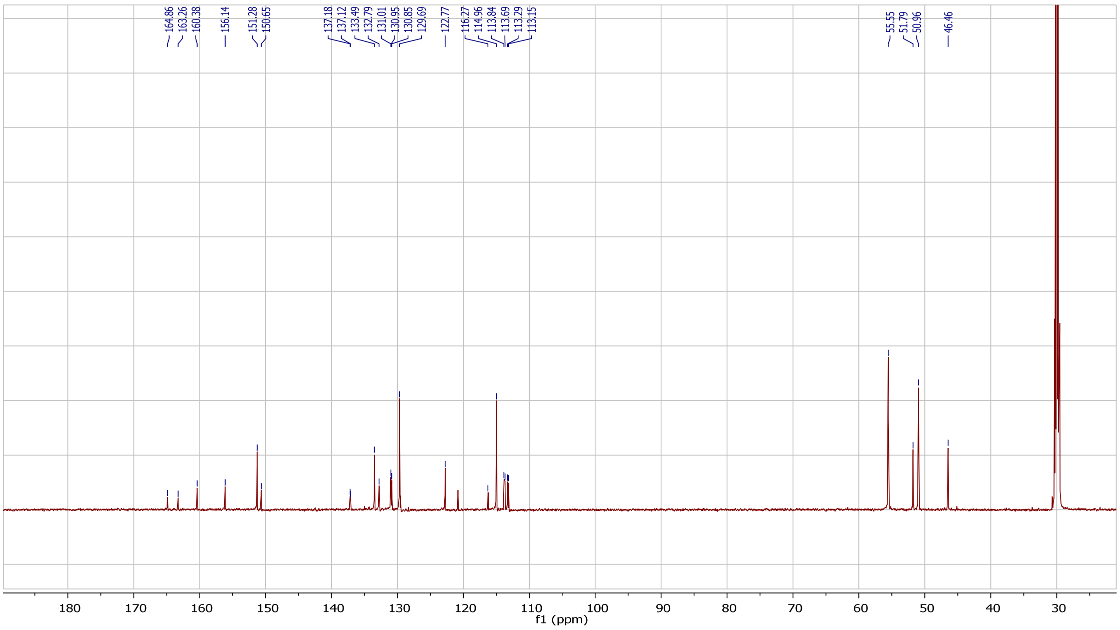

Figure S36. ^{1}H and ^{13}C NMR spectra of compound **25b** in acetone-d_6.

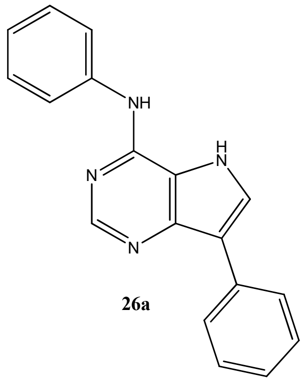

26a

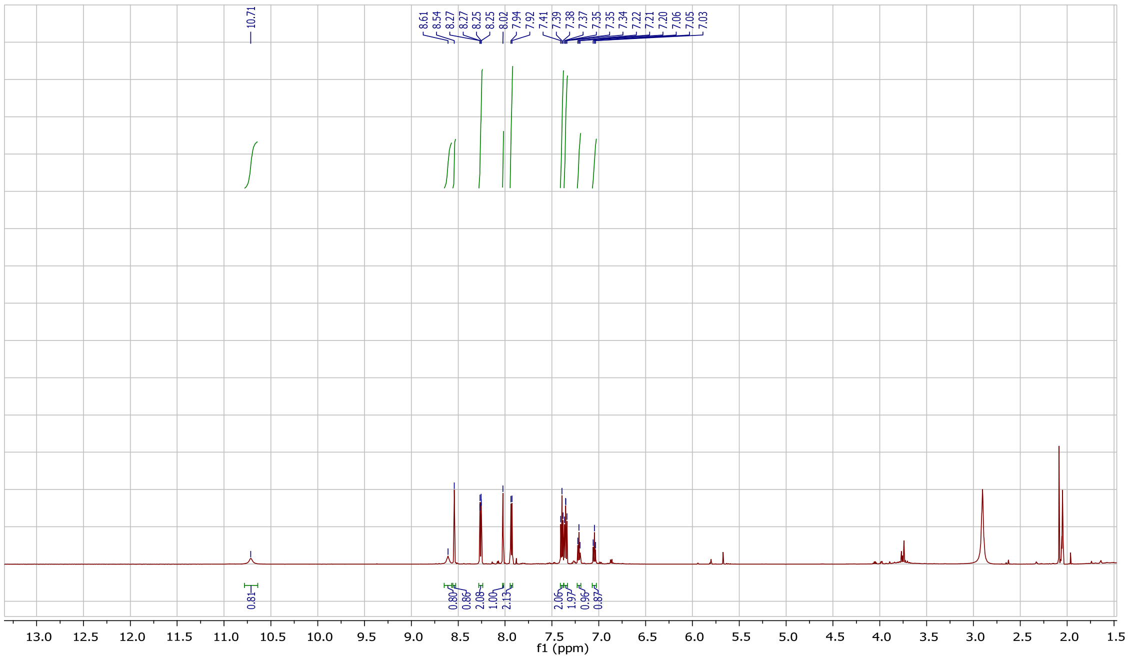

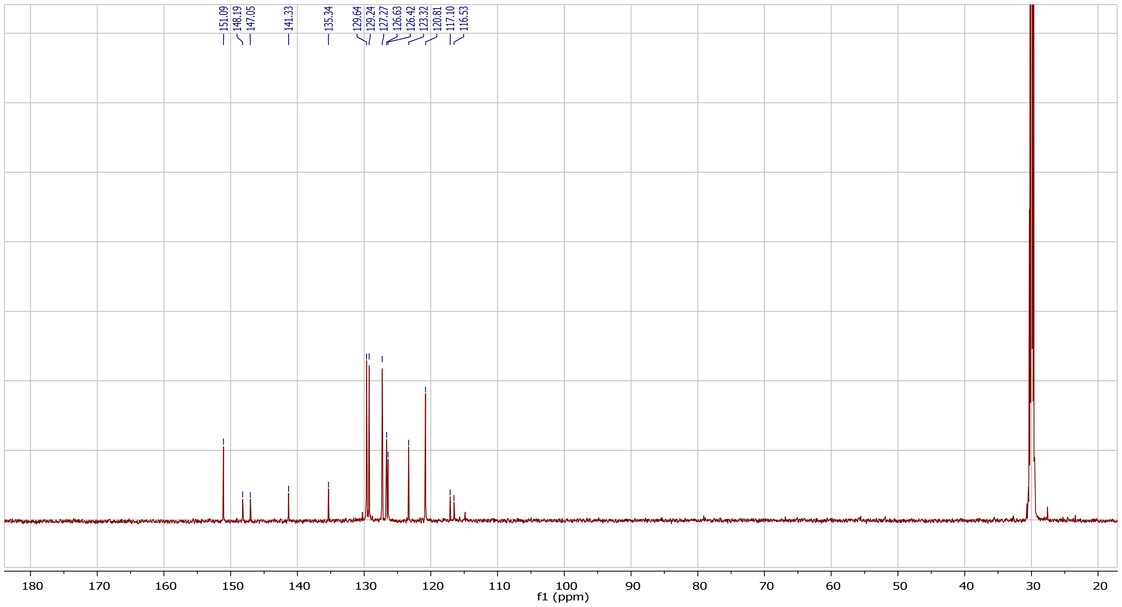

Figure S37. ^{1}H and ^{13}C NMR spectra of compound **26a** in acetone-d_6.

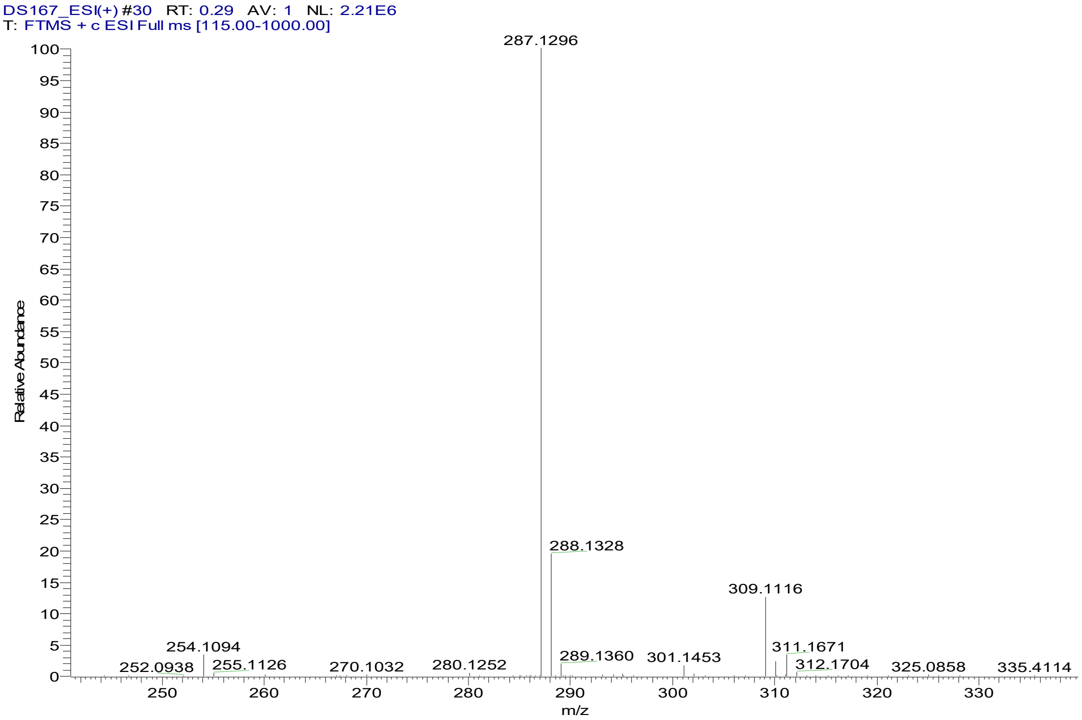

Figure S38. HRMS spectrum (ESI +) of compound **26a.**

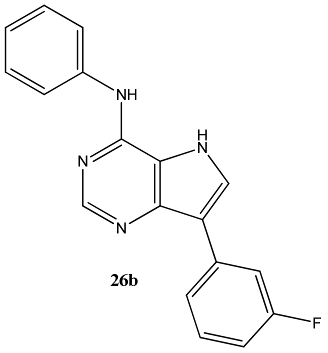

26b

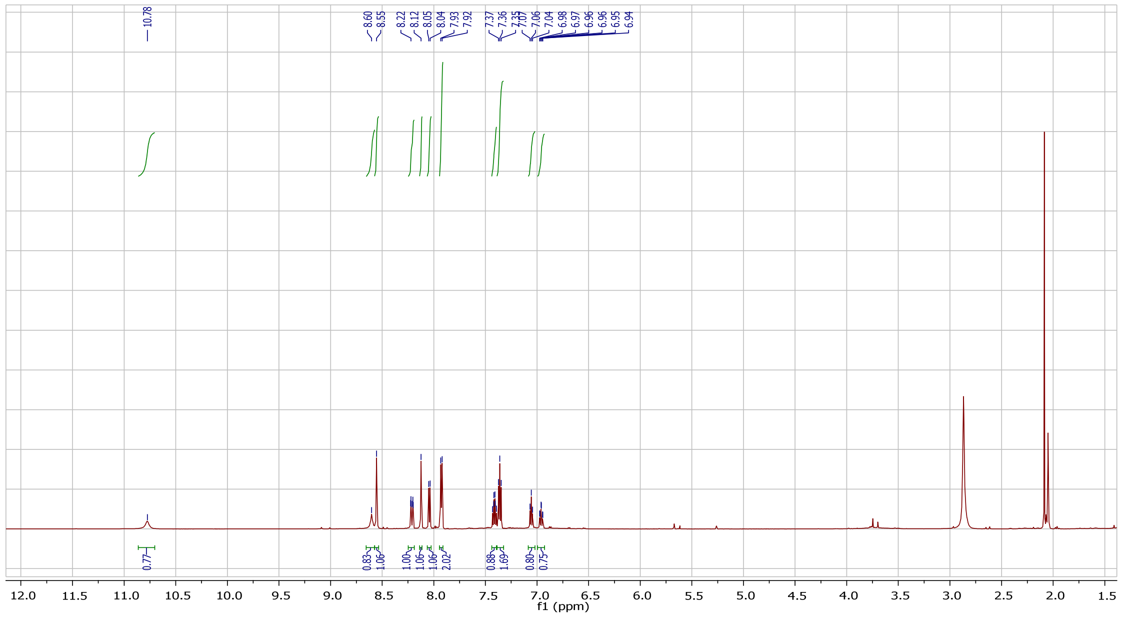

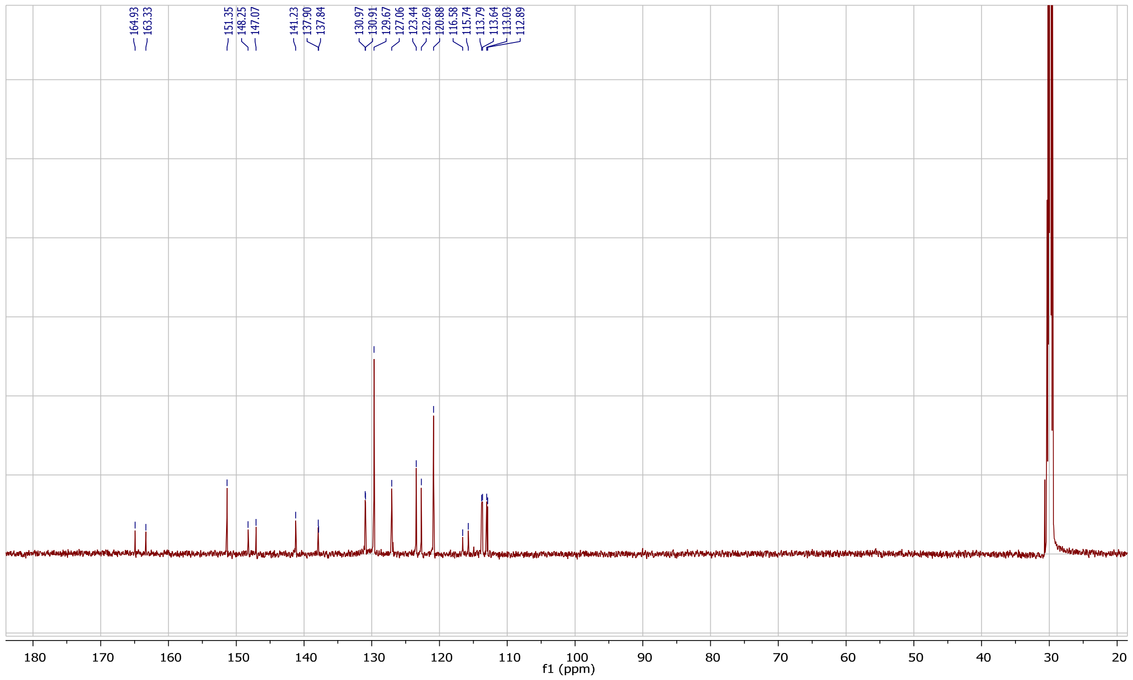

Figure S39. ^{1}H and ^{13}C NMR spectra of compound **26b** in acetone-d_6.

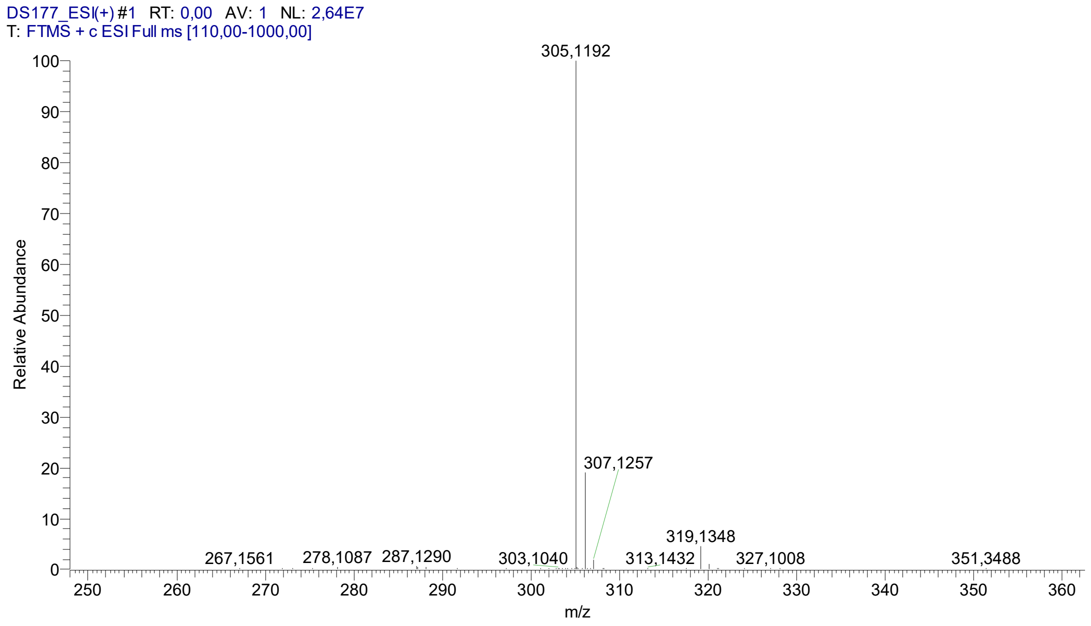

Figure S40. HRMS spectrum (ESI +) of compound **26b.**

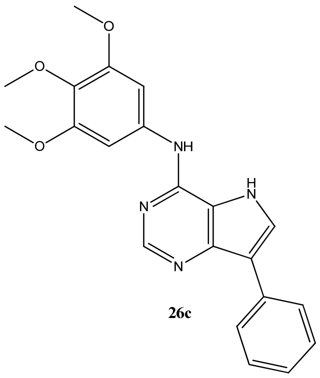

26c

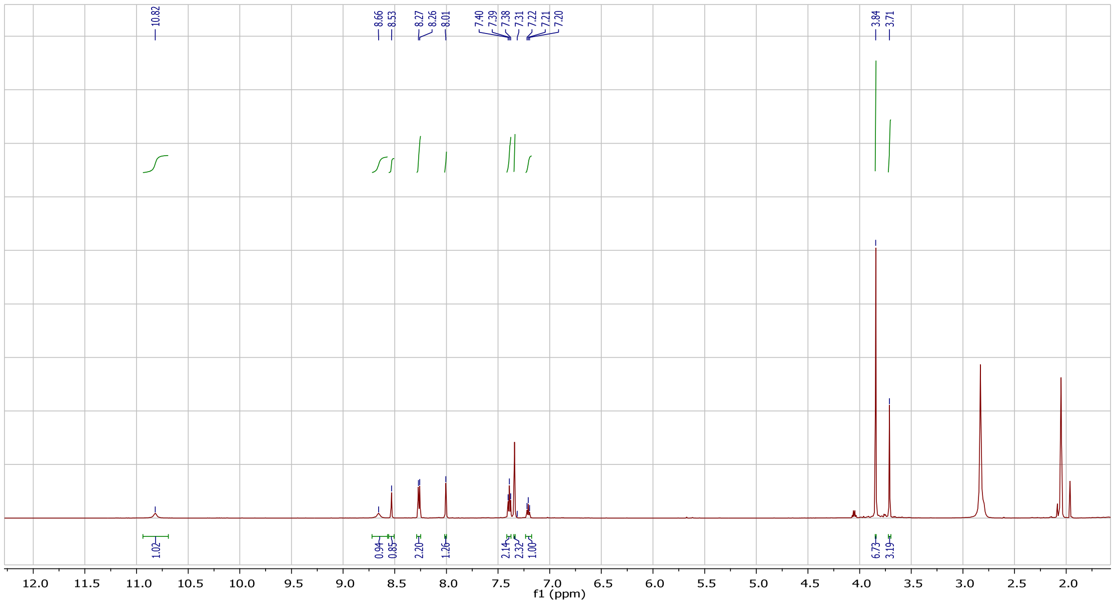

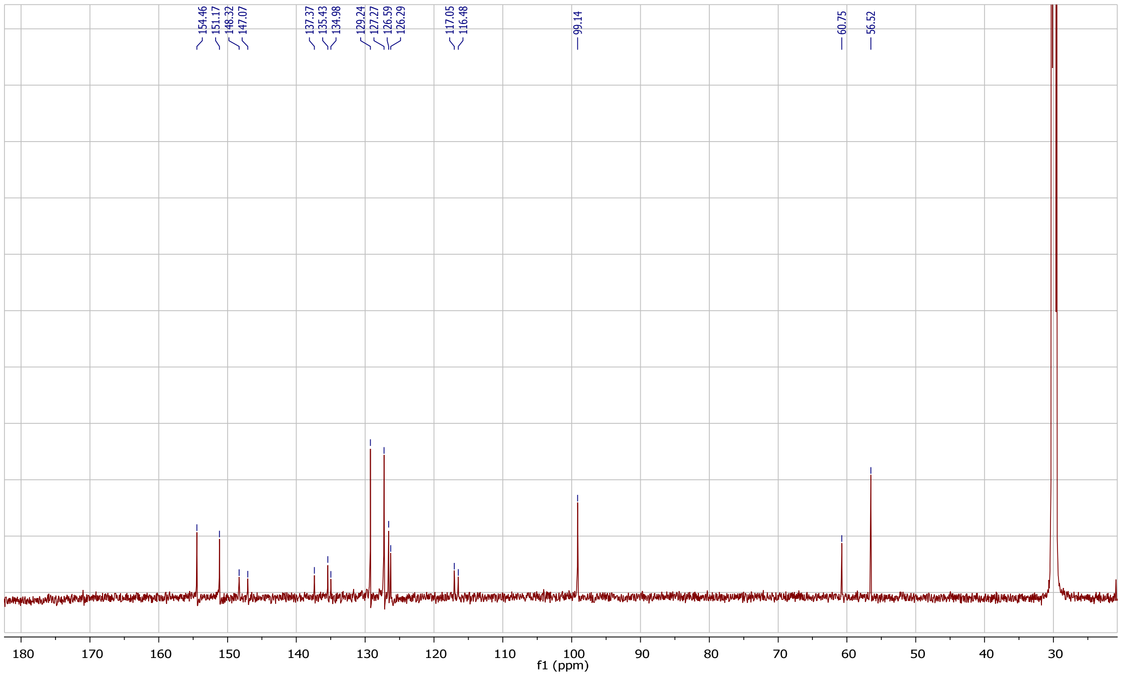

Figure S41. ^{1}H and ^{13}C NMR spectra of compound **26c** in acetone-d_6.

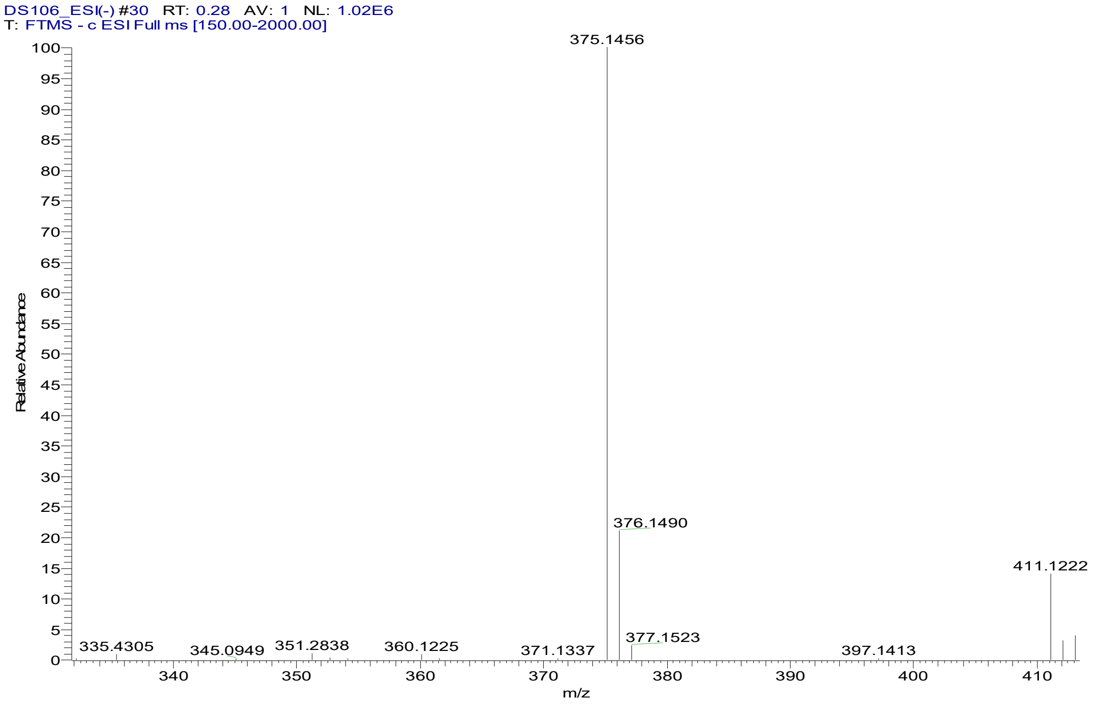

Figure S42. HRMS spectrum (ESI -) of compound **26c.**

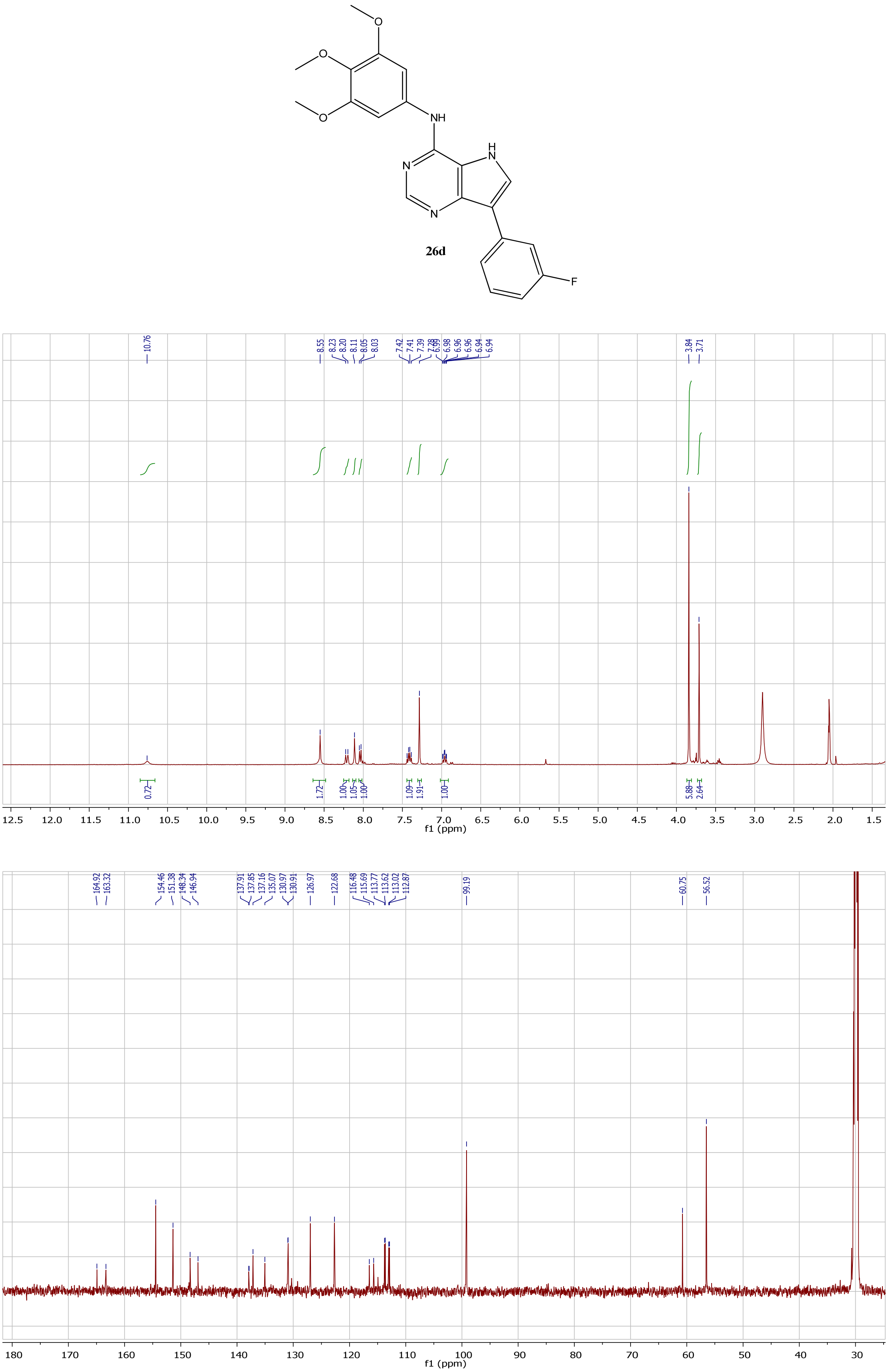

Figure S43. ^{1}H and ^{13}C NMR spectra of compound **26d** in acetone-d_6.

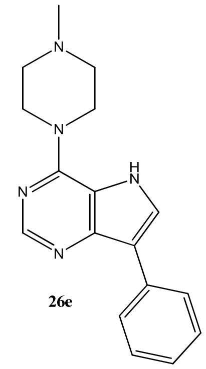

26e

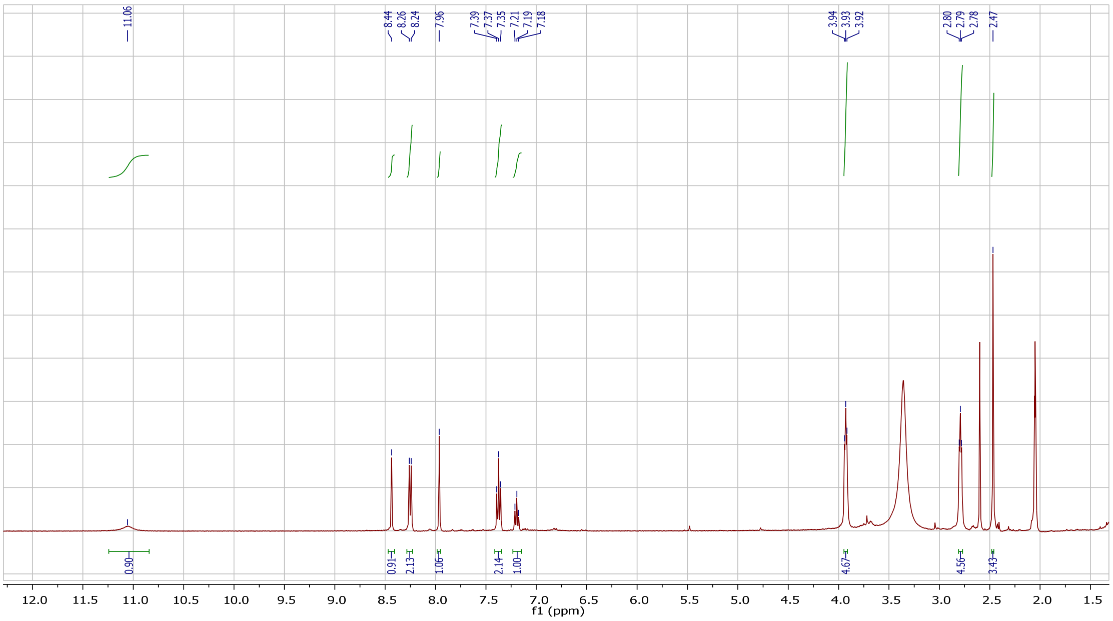

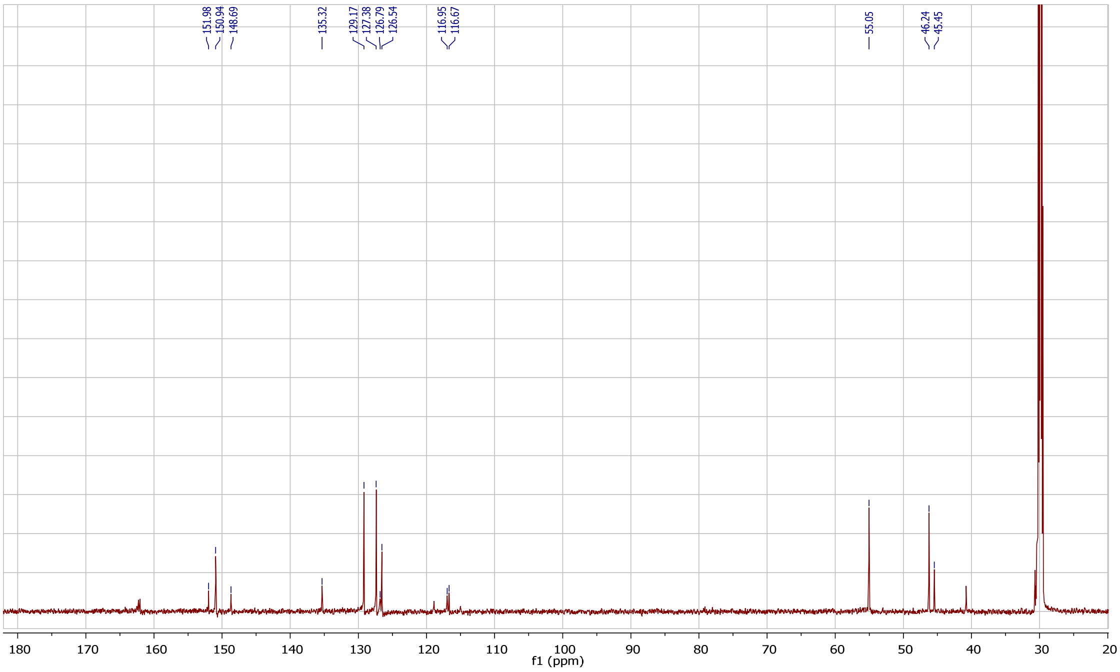

Figure S44. ^{1}H and ^{13}C NMR spectra of compound **26e** in acetone-d_6.

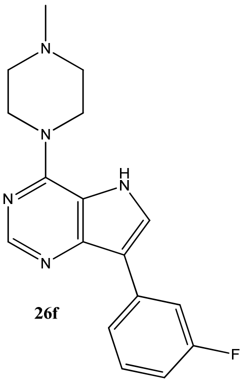

26f

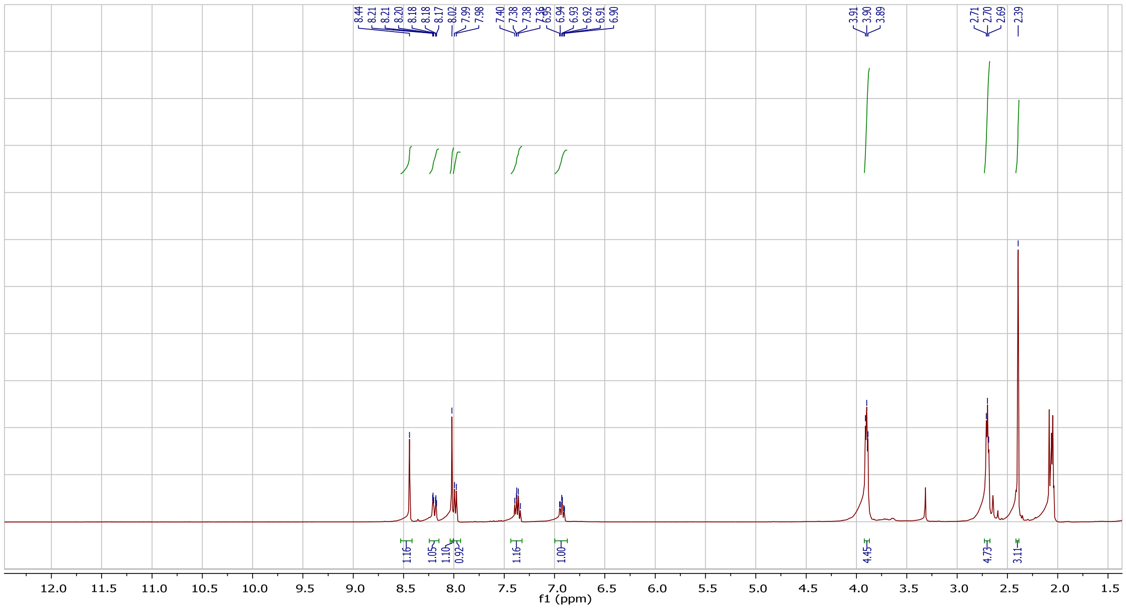

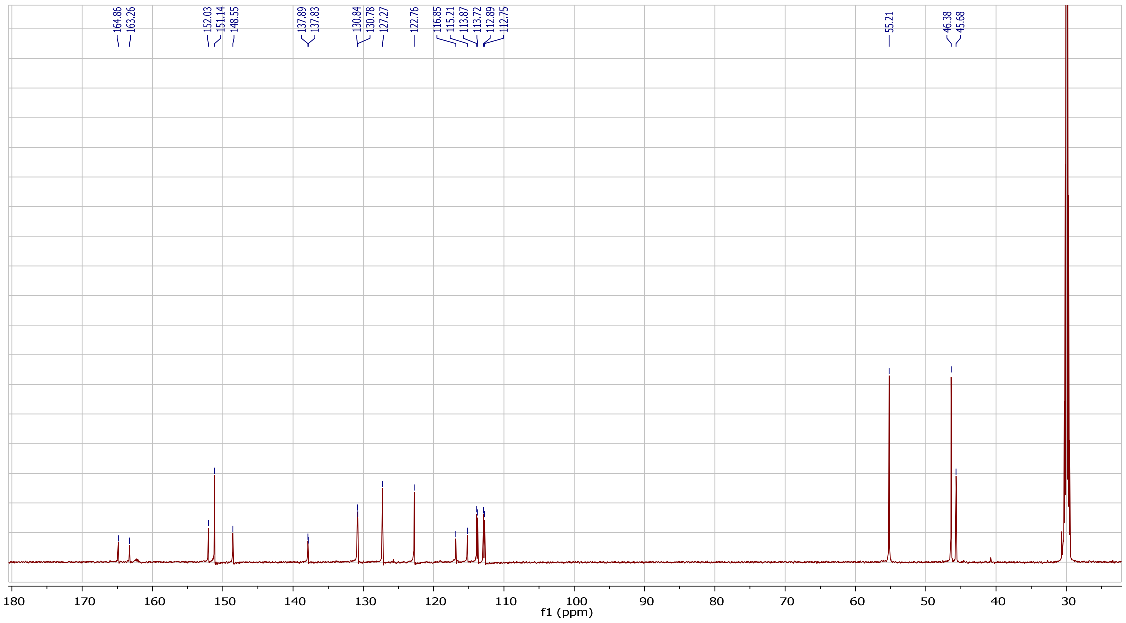

Figure S45. ^{1}H and ^{13}C NMR spectra of compound **26f** in acetone-d_6.

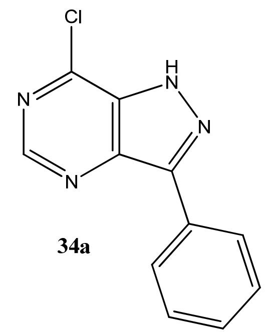

34a

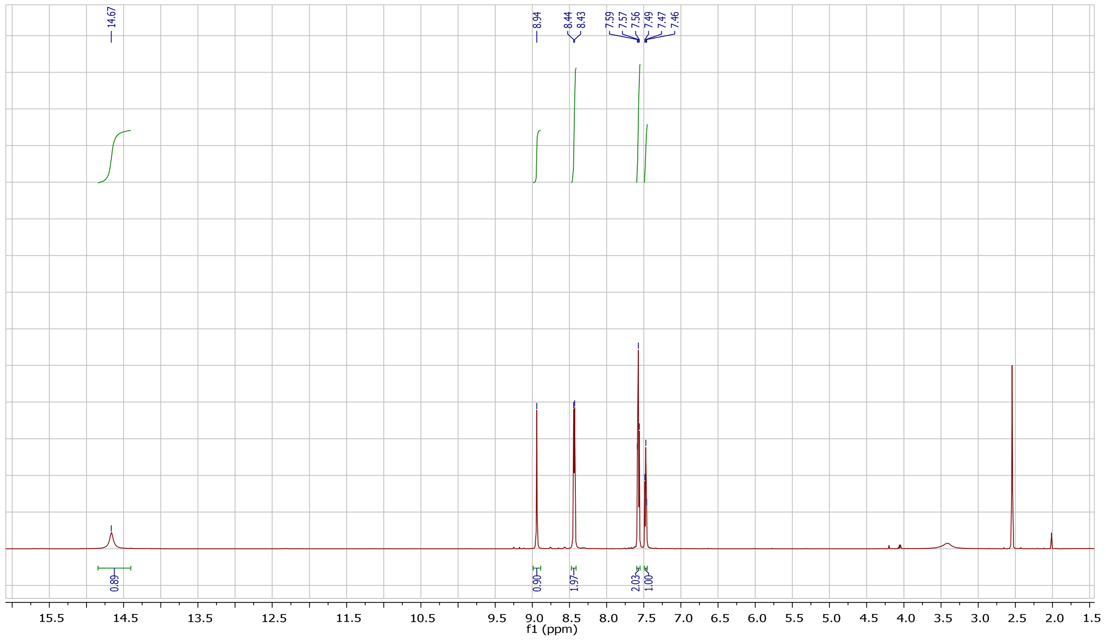

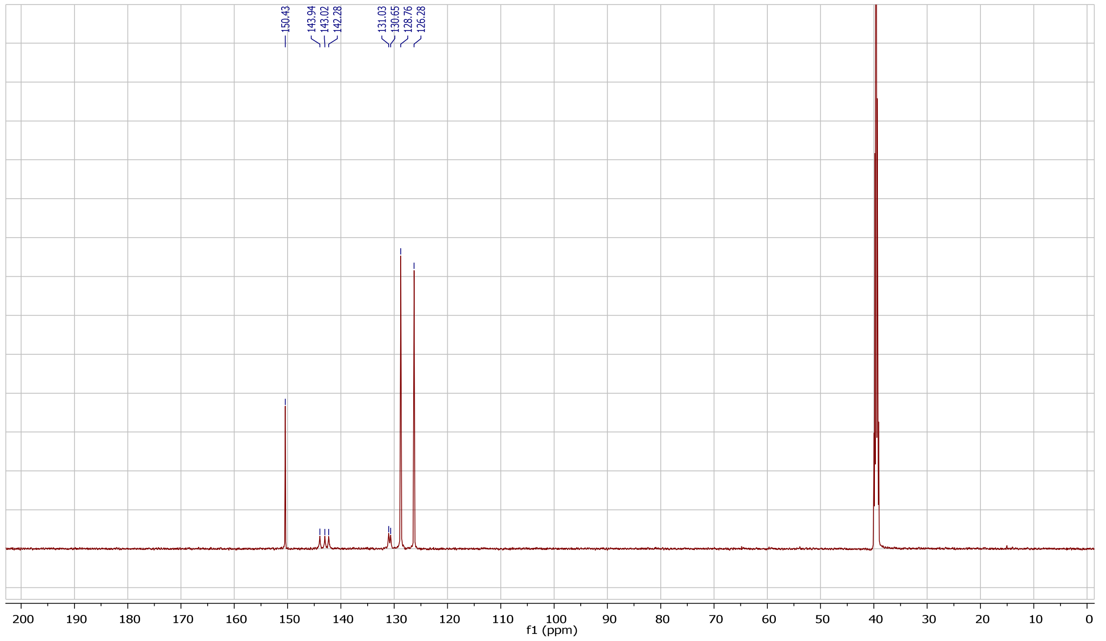

Figure S46. ^{1}H and ^{13}C NMR spectra of compound **34a** in DMSO-d_6.

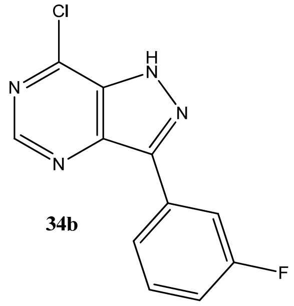

34b

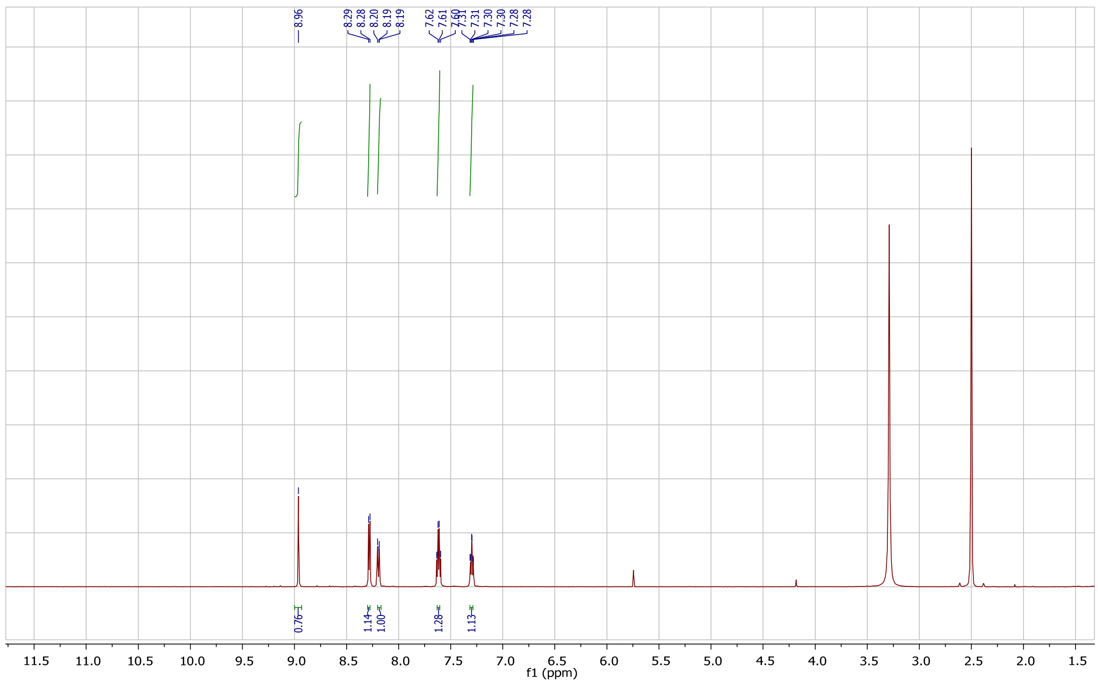

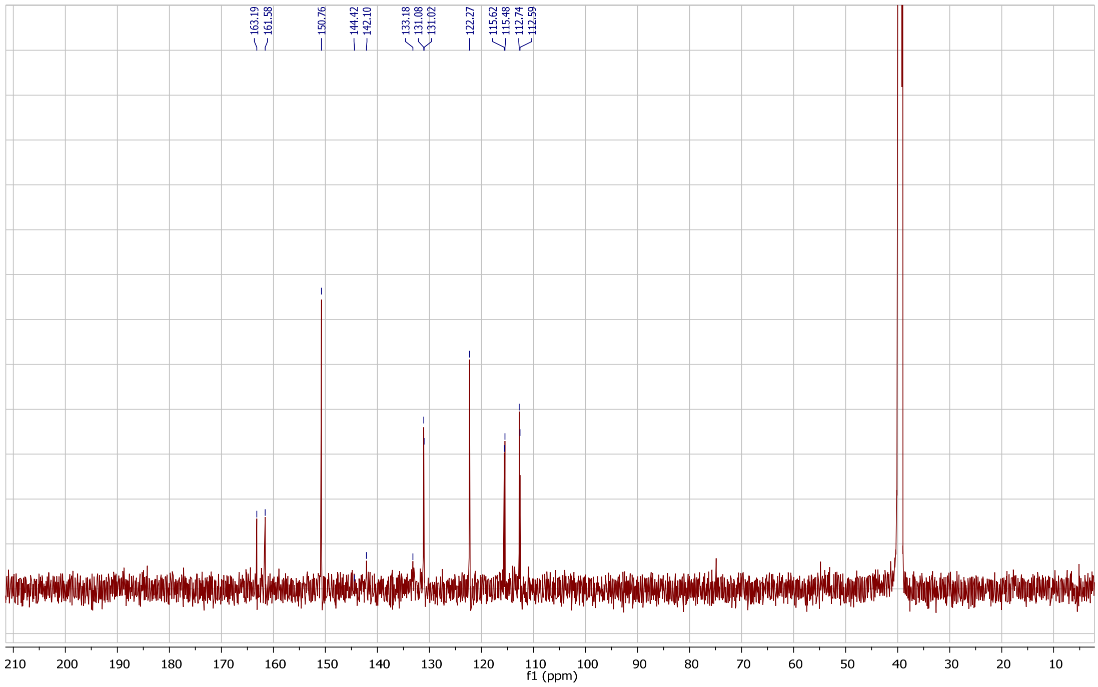

Figure S47. ^{1}H and ^{13}C NMR spectra of compound **34b** in DMSO-d_6.

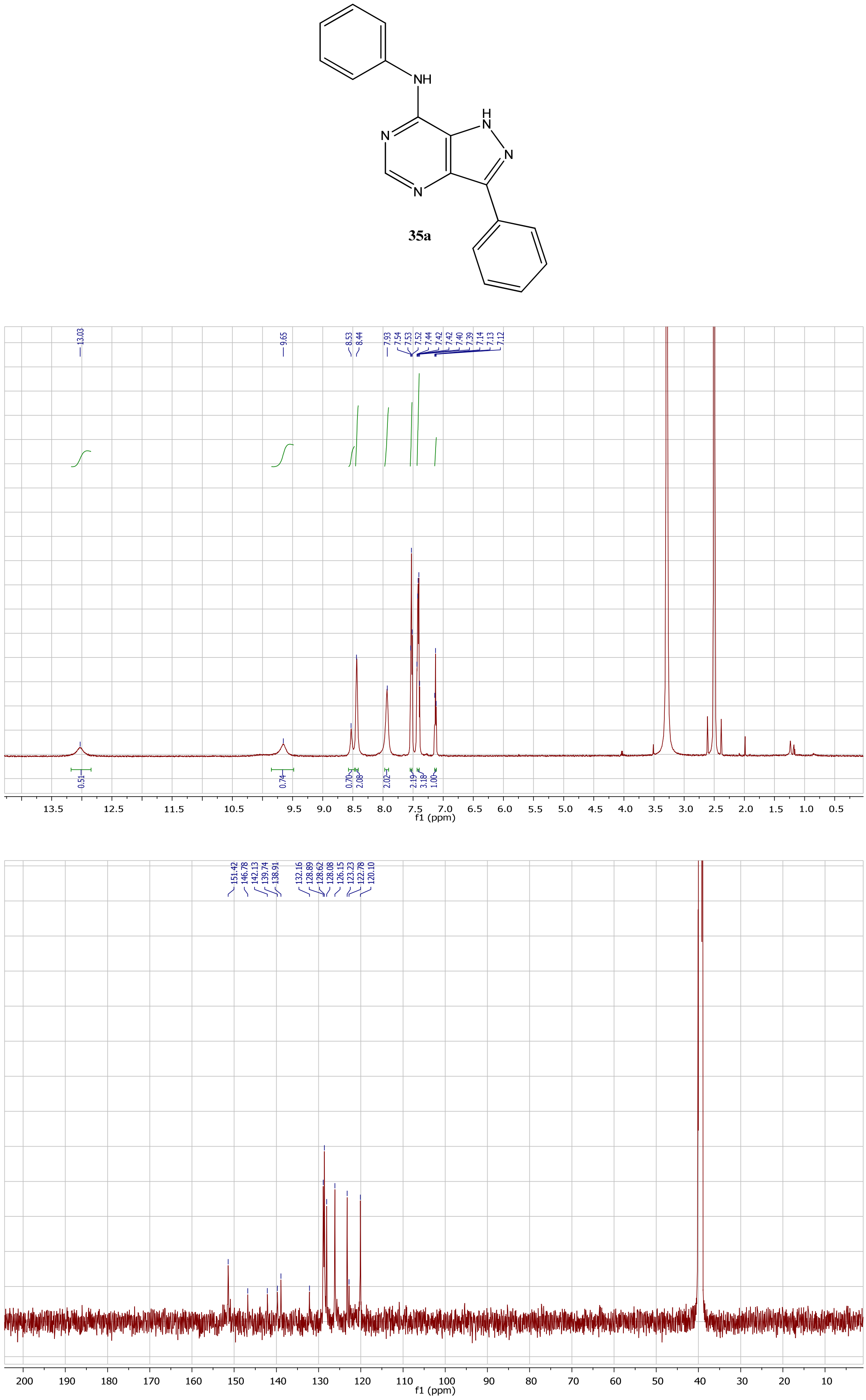

Figure S48. ^{1}H and ^{13}C NMR spectra of compound **35a** in DMSO-d_6.

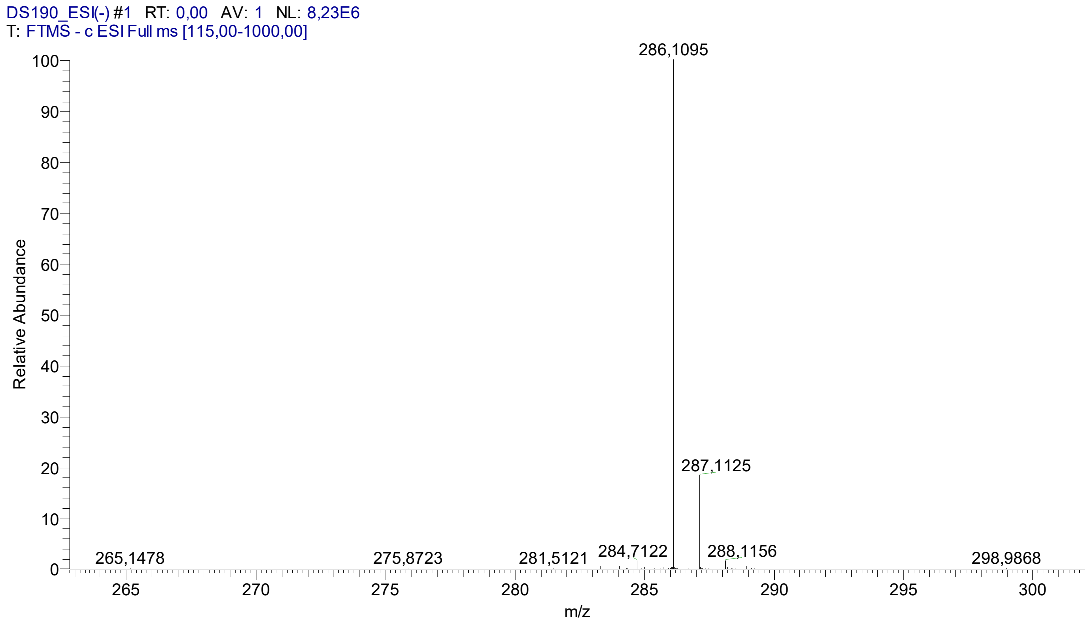

Figure S49. HRMS spectrum (ESI -) of compound **35a.**

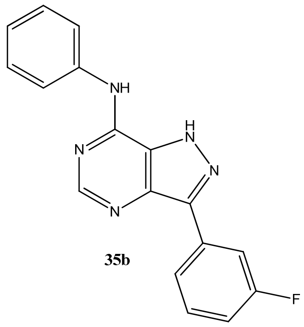

35b

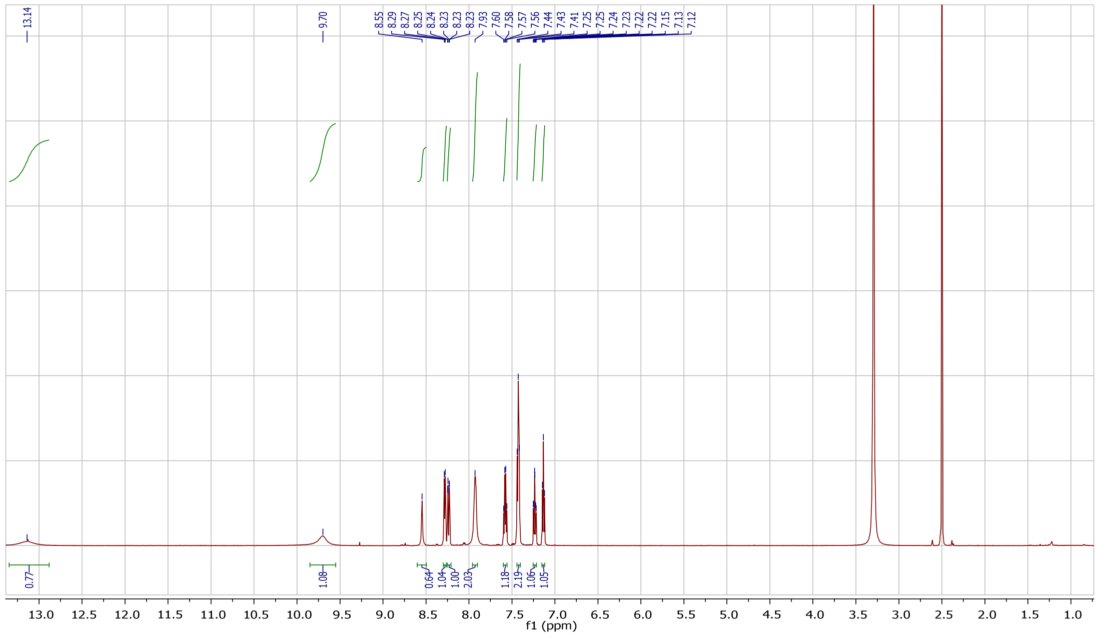

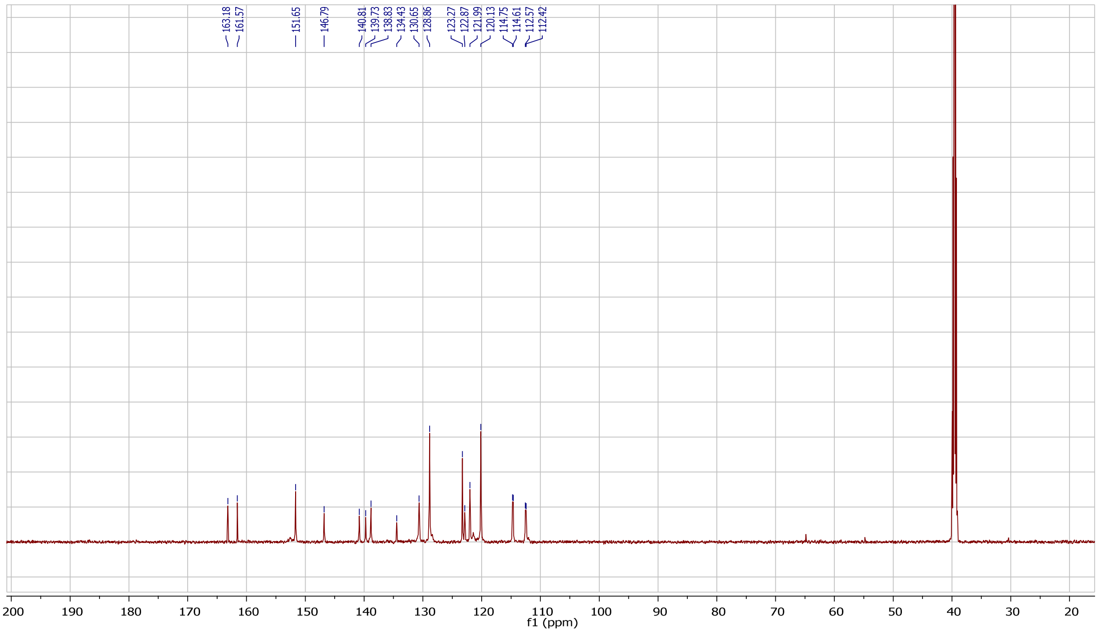

Figure S50. ^{1}H and ^{13}C NMR spectra of compound **35b** in DMSO-d_6.

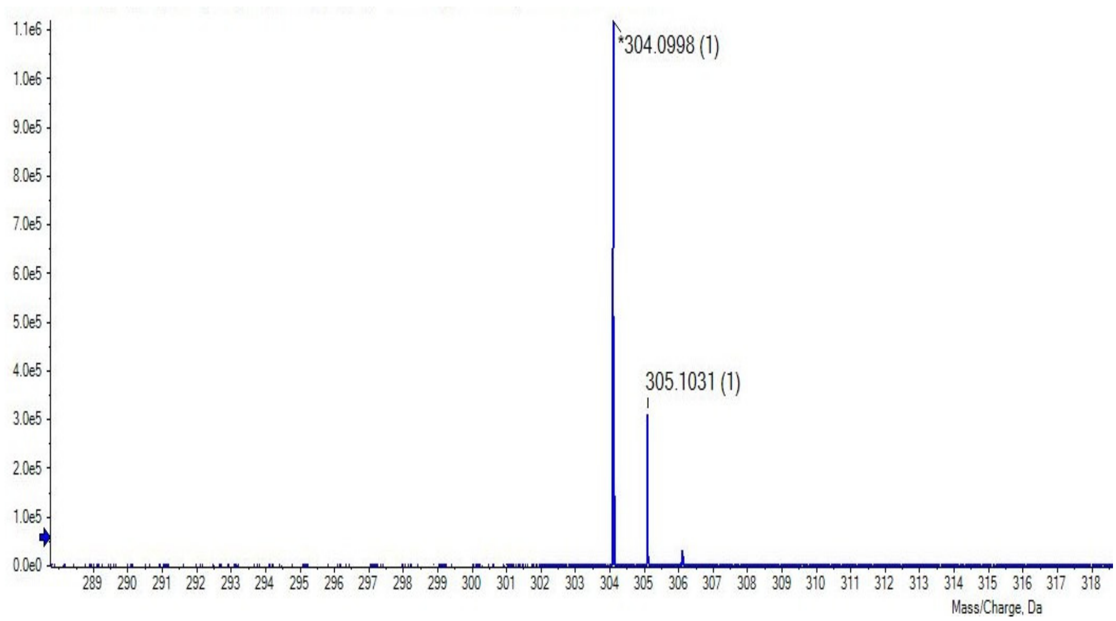

Figure S51. HRMS spectrum (ESI -) of compound **35b.**

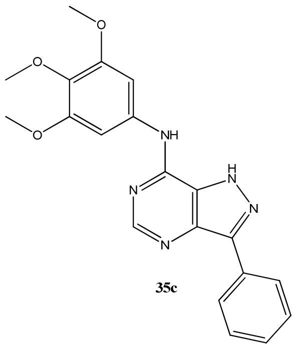

35c

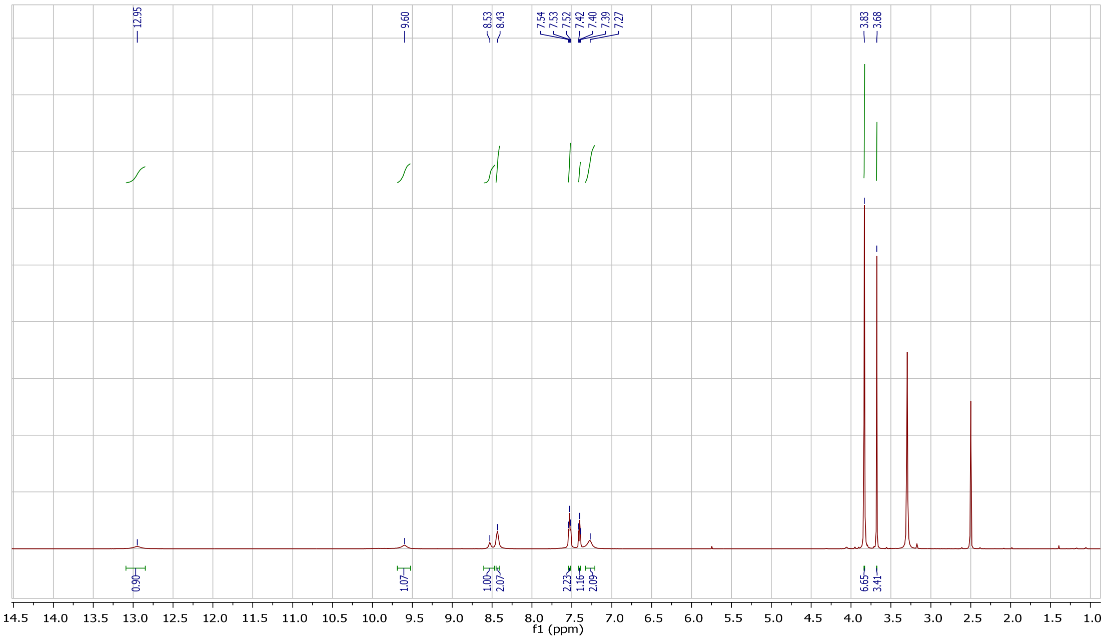

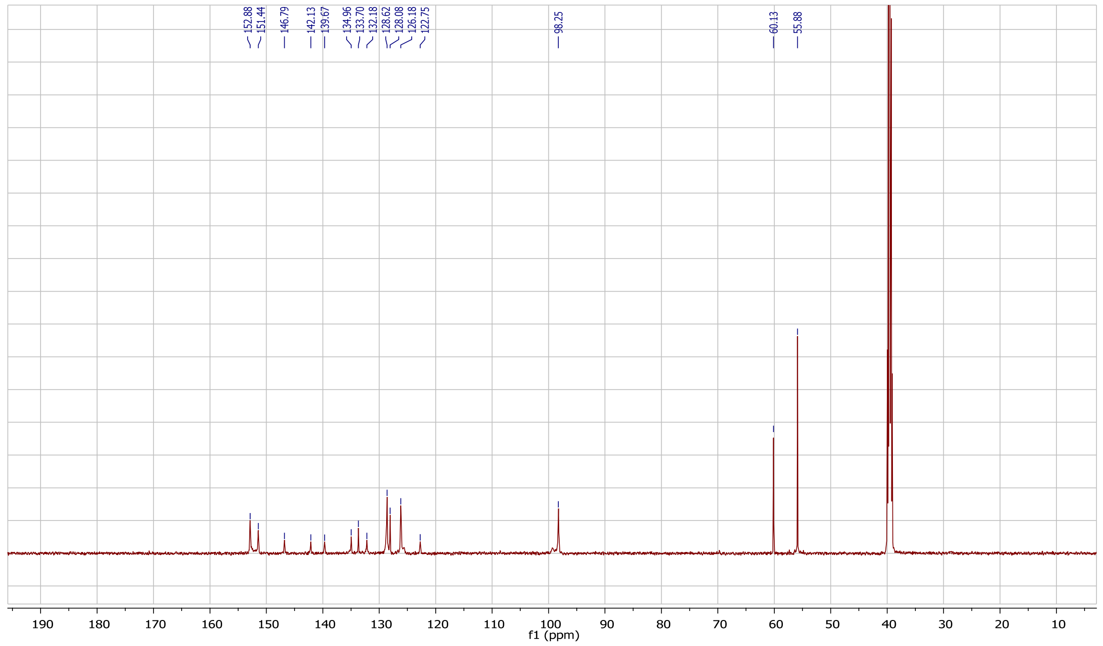

Figure S52. ^{1}H and ^{13}C NMR spectra of compound **35c** in DMSO-d_6.

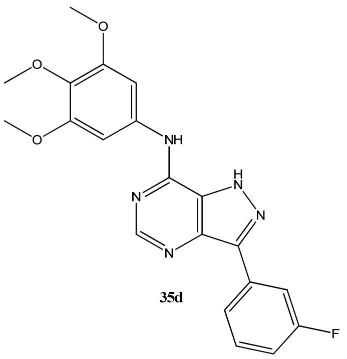

35d

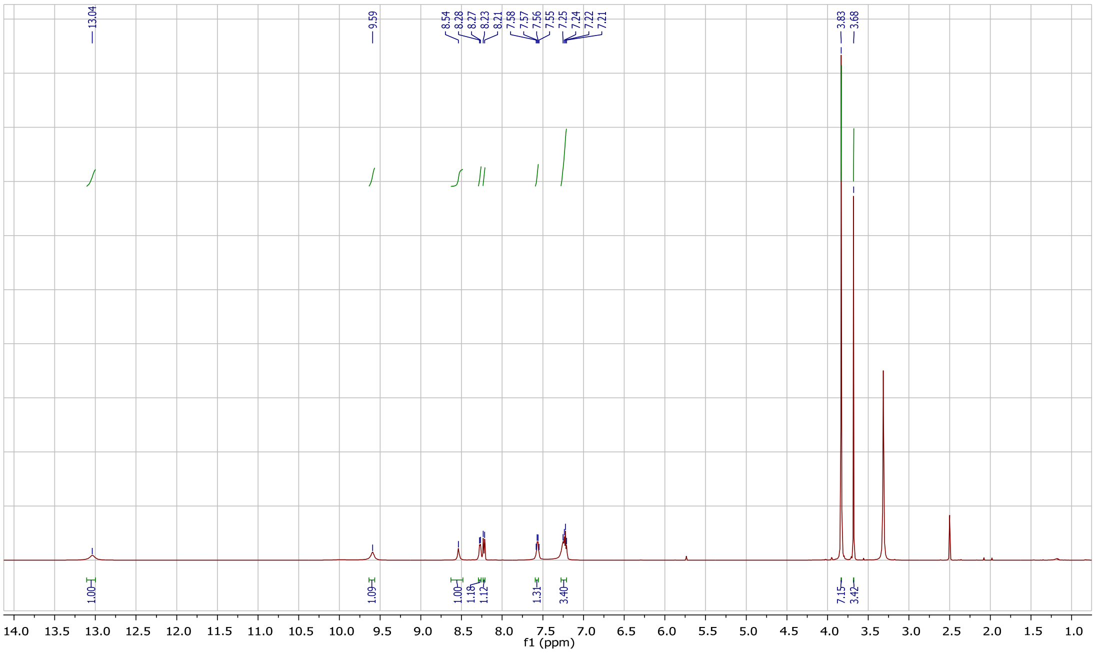

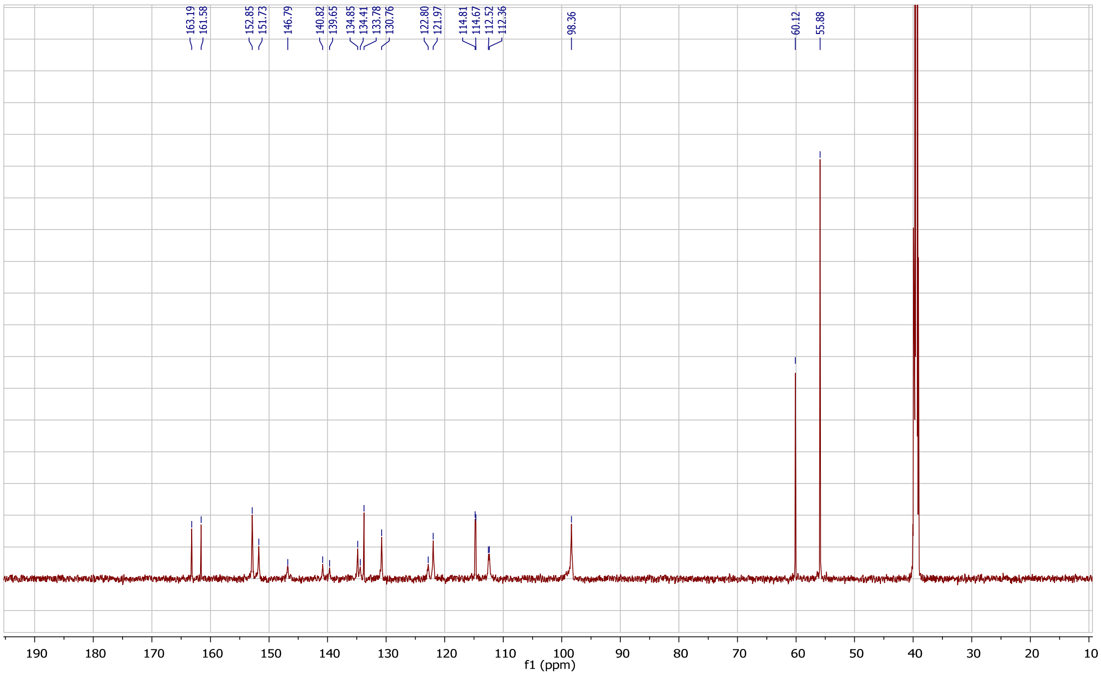

Figure S53. ^{1}H and ^{13}C NMR spectra of compound **35d** in DMSO-d_6.

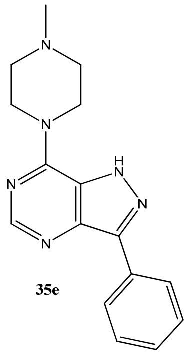

35e

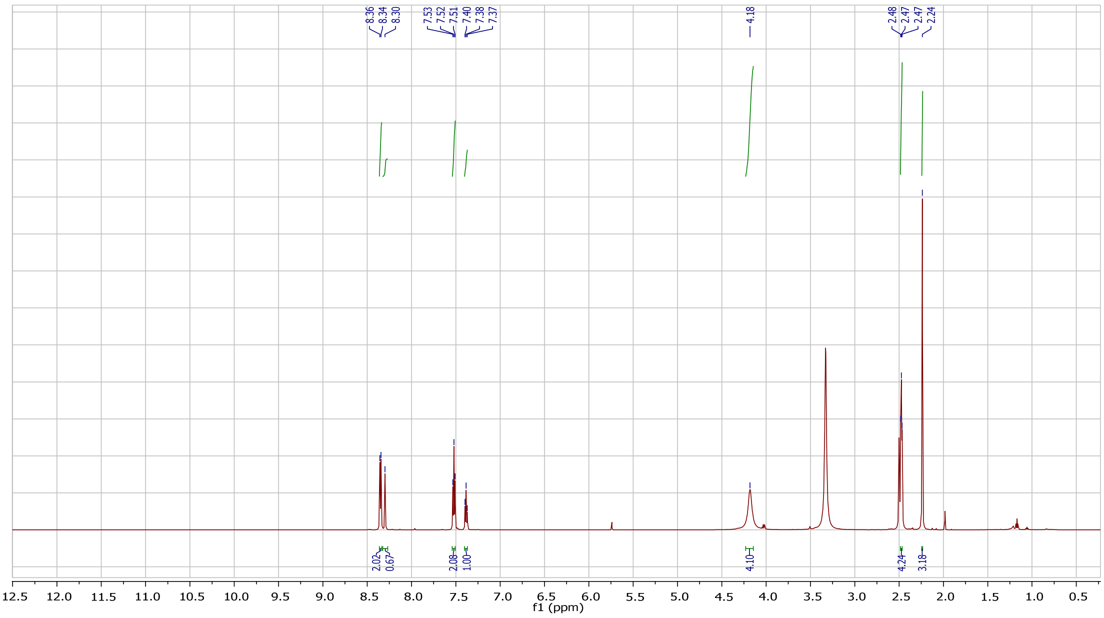

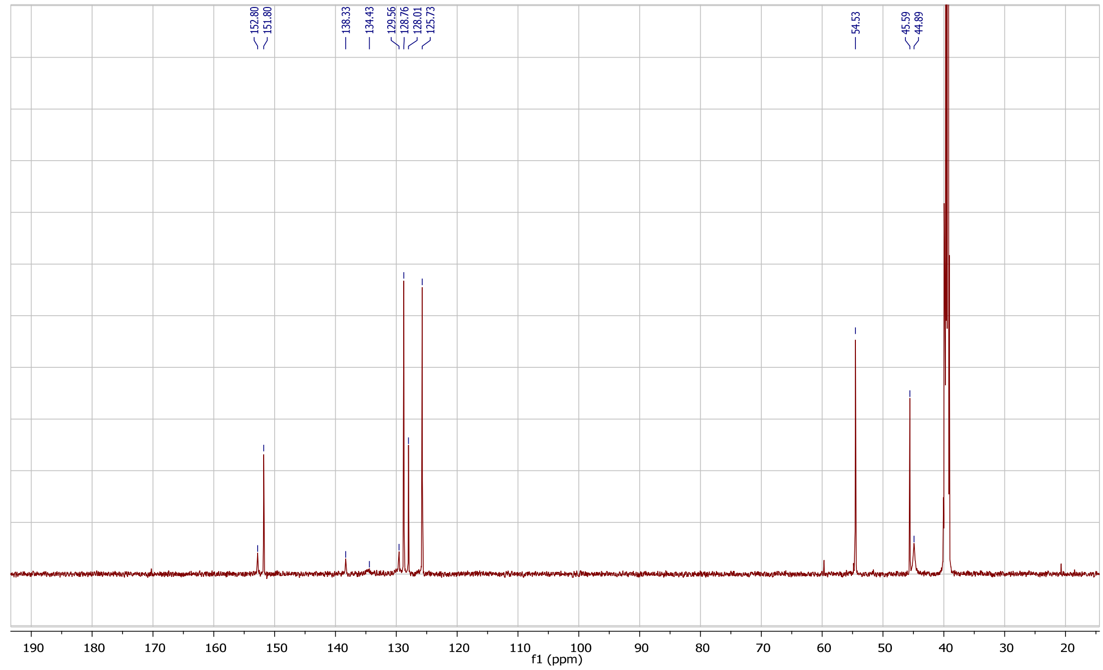

Figure S54. ^{1}H and ^{13}C NMR spectra of compound **35e** in DMSO-d_6.

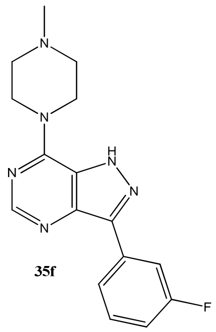

35f

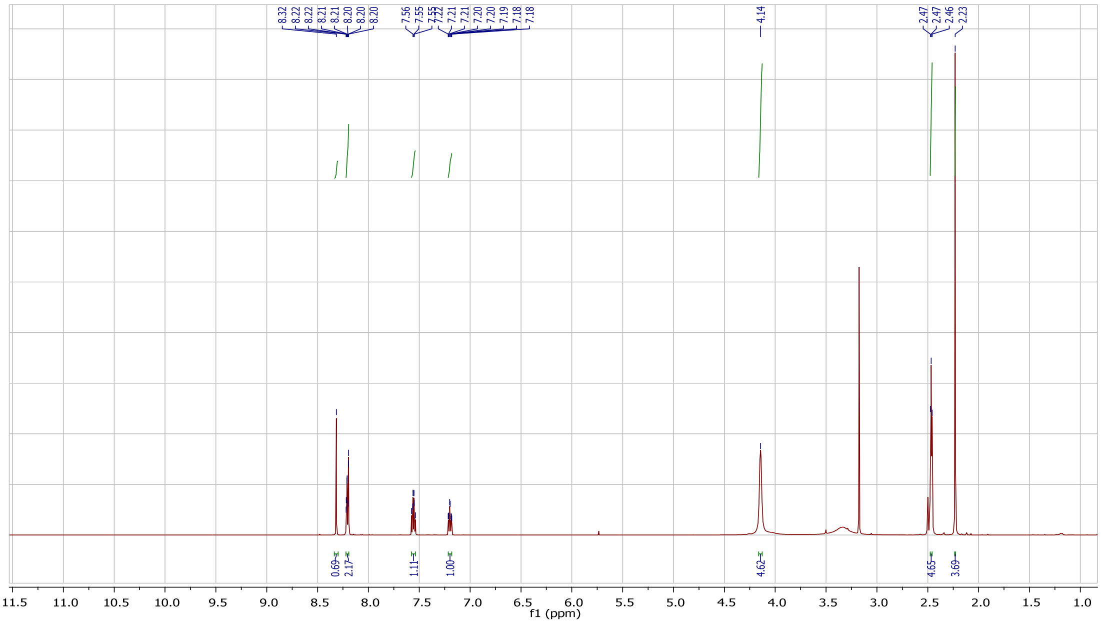

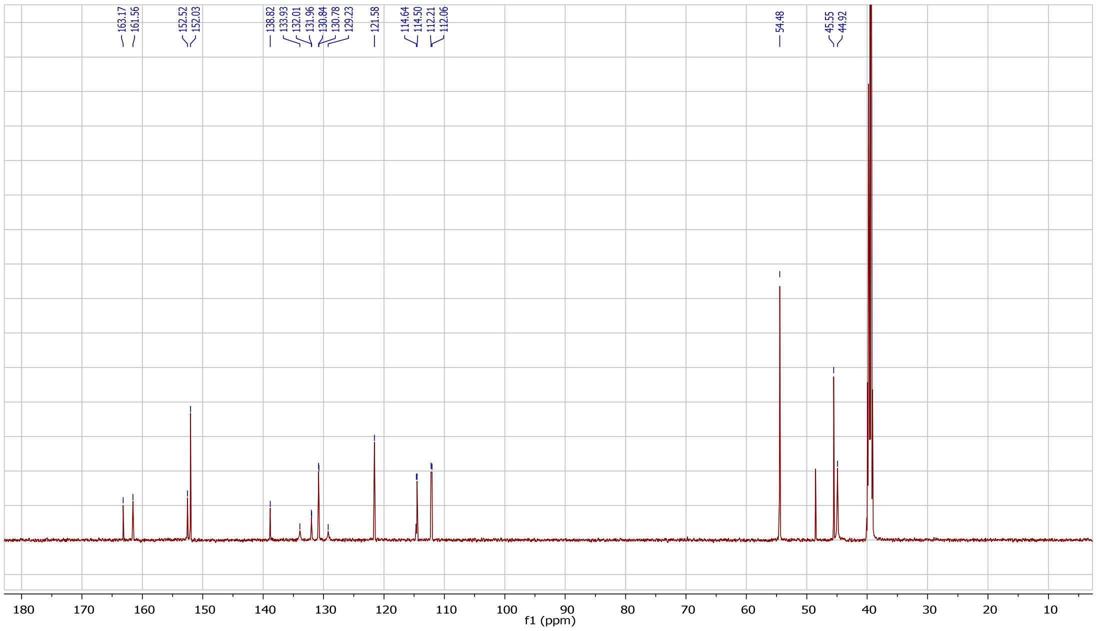

Figure S55. ^{1}H and ^{13}C NMR spectra of compound **35f** in DMSO-d_6.

Table S1. Calculated physicochemical characteristics of the tested compounds using SwissADME

Compound	MW	#Heavy atoms	#Aromatic heavy atoms	Fraction Csp3	#Rotatable bonds	#H-bond acceptors	#H-bond donors	MR	TPSA	iLOGP	XLOGP3	WLOGP	MLOGP	Silicos-IT Log P	Consensus Log P
10a	405.49	31	27	0.07	6	2	1	126.95	39.08	3.83	6	6.5	4.11	5.19	5.13
10b	423.48	32	27	0.07	6	3	1	126.91	39.08	3.89	6.1	7.06	4.47	5.6	5.43
10c	495.57	37	27	0.17	9	5	1	146.43	66.77	4.27	5.91	6.53	3.05	5.38	5.03
10d	513.56	38	27	0.17	9	6	1	146.39	66.77	4.33	6.01	7.09	3.42	5.81	5.33
13a	412.53	31	21	0.27	5	3	0	133.67	33.53	4.12	4.39	3.75	3.02	3.93	3.84
13b	430.52	32	21	0.27	5	4	0	133.63	33.53	4.13	4.49	4.31	3.39	4.35	4.13
14a	285.34	22	21	0	3	1	2	91.07	40.71	2.23	4.48	4.97	3.19	4.29	3.83
14b	303.33	23	21	0	3	2	2	91.03	40.71	2.39	4.58	5.53	3.57	4.68	4.15
14c	375.42	28	21	0.14	6	4	2	110.55	68.4	2.9	4.39	5	2.17	4.39	3.77
14d	393.41	29	21	0.14	6	5	2	110.51	68.4	3	4.49	5.56	2.55	4.81	4.08
14e	292.38	22	15	0.28	2	2	1	97.79	35.16	2.57	2.87	2.22	2.06	3.04	2.55
14f	310.37	23	15	0.28	2	3	1	97.75	35.16	2.94	2.97	2.78	2.45	3.44	2.92
24a	406.48	31	27	0.08	6	3	1	124.75	51.97	3.83	5.44	5.9	3.75	4.61	4.71
24b	424.47	32	27	0.08	6	4	1	124.71	51.97	3.93	5.54	6.46	4.12	5.03	5.01
24c	496.56	37	27	0.17	9	6	1	144.22	79.66	4.54	5.35	5.92	2.75	4.81	4.68
24d	514.55	38	27	0.17	9	7	1	144.18	79.66	4.17	5.45	6.48	3.11	5.23	4.89
25a	413.51	31	21	0.28	5	4	0	131.47	46.42	3.95	3.83	3.15	2.27	3.36	3.31
25b	431.51	32	21	0.28	5	5	0	131.42	46.42	4.13	3.93	3.7	2.64	3.77	3.64
26a	286.33	22	21	0	3	2	2	88.87	53.6	2.15	3.92	4.37	2.78	3.72	3.39
26b	304.32	23	21	0	3	3	2	88.83	53.6	2.3	4.02	4.93	3.17	4.12	3.71
26c	376.41	28	21	0.14	6	5	2	108.34	81.29	2.75	3.83	4.39	1.83	3.82	3.32
26d	394.4	29	21	0.14	6	6	2	108.3	81.29	2.96	3.93	4.95	2.2	4.24	3.66
26e	293.37	22	15	0.29	2	3	1	95.58	48.05	2.7	2.31	1.62	1.67	2.48	2.15
26f	311.36	23	15	0.29	2	4	1	95.54	48.05	2.81	2.41	2.17	2.06	2.88	2.47
35a	287.32	22	21	0	3	3	2	86.66	66.49	1.74	3.46	3.76	2.53	3.16	2.93
35b	305.31	23	21	0	3	4	2	86.62	66.49	2.03	3.56	4.32	2.91	3.56	3.28
35c	377.4	28	21	0.15	6	6	2	106.14	94.18	2.37	3.37	3.79	1.63	3.26	2.88
35d	395.39	29	21	0.15	6	7	2	106.1	94.18	2.55	3.47	4.35	2.01	3.68	3.21
35e	294.35	22	15	0.31	2	4	1	93.38	60.94	2.38	1.85	1.01	1.42	1.93	1.72
35f	312.34	23	15	0.31	2	5	1	93.34	60.94	2.53	1.95	1.57	1.81	2.33	2.04

Table S1. cont.

compound	ESOL Log S	ESOL Solubility (mg/ml)	ESOL Solubility (mol/l)	Ali Log S	Ali Solubility (mg/ml)	Ali Solubility (mol/l)	Silicos-IT LogSw	Silicos-IT Solubility (mg/ml)	Silicos-IT Solubility (mol/l)	log Kp (cm/s)
10a	-6.38	1.68E-04	4.14E-07	-6.6	1.02E-04	2.52E-07	-10.14	2.96E-08	7.29E-11	-4.51
10b	-6.54	1.23E-04	2.90E-07	-6.7	8.41E-05	1.99E-07	-10.4	1.68E-08	3.97E-11	-4.55
10c	-6.58	1.30E-04	2.62E-07	-7.09	4.06E-05	8.20E-08	-10.43	1.84E-08	3.71E-11	-5.13
10d	-6.74	9.30E-05	1.81E-07	-7.19	3.32E-05	6.46E-08	-10.69	1.05E-08	2.05E-11	-5.17
13a	-5.33	1.91E-03	4.63E-06	-4.81	6.38E-03	1.55E-05	-7.73	7.65E-06	1.85E-08	-5.7
13b	-5.49	1.38E-03	3.21E-06	-4.91	5.24E-03	1.22E-05	-7.99	4.37E-06	1.01E-08	-5.74
14a	-4.94	3.28E-03	1.15E-05	-5.06	2.51E-03	8.81E-06	-7.99	2.91E-06	1.02E-08	-4.86
14b	-5.08	2.50E-03	8.25E-06	-5.16	2.10E-03	6.94E-06	-8.26	1.66E-06	5.46E-09	-4.9
14c	-5.09	3.04E-03	8.09E-06	-5.54	1.08E-03	2.86E-06	-8.32	1.79E-06	4.76E-09	-5.47
14d	-5.25	2.22E-03	5.65E-06	-5.65	8.87E-04	2.25E-06	-8.59	1.02E-06	2.59E-09	-5.51
14e	-3.83	4.29E-02	1.47E-04	-3.27	1.58E-01	5.40E-04	-5.59	7.53E-04	2.58E-06	-6.05
14f	-3.99	3.21E-02	1.03E-04	-3.37	1.32E-01	4.25E-04	-5.86	4.28E-04	1.38E-06	-6.08
24a	-6.04	3.74E-04	9.21E-07	-6.29	2.10E-04	5.16E-07	-9.76	7.00E-08	1.72E-10	-4.92
24b	-6.19	2.74E-04	6.45E-07	-6.39	1.72E-04	4.06E-07	-10.03	3.99E-08	9.40E-11	-4.96
24c	-6.24	2.89E-04	5.82E-07	-6.78	8.32E-05	1.68E-07	-10.06	4.35E-08	8.77E-11	-5.53
24d	-6.4	2.07E-04	4.02E-07	-6.88	6.79E-05	1.32E-07	-10.32	2.49E-08	4.83E-11	-5.57
25a	-4.99	4.25E-03	1.03E-05	-4.5	1.31E-02	3.16E-05	-7.36	1.81E-05	4.38E-08	-6.1
25b	-5.15	3.08E-03	7.13E-06	-4.6	1.07E-02	2.49E-05	-7.62	1.03E-05	2.39E-08	-6.14
26a	-4.59	7.31E-03	2.55E-05	-4.74	5.16E-03	1.80E-05	-7.62	6.88E-06	2.40E-08	-5.26
26b	-4.74	5.58E-03	1.83E-05	-4.85	4.31E-03	1.42E-05	-7.89	3.91E-06	1.29E-08	-5.3
26c	-4.75	6.76E-03	1.80E-05	-5.23	2.20E-03	5.85E-06	-7.95	4.23E-06	1.12E-08	-5.88
26d	-4.9	4.95E-03	1.26E-05	-5.34	1.82E-03	4.61E-06	-8.21	2.41E-06	6.11E-09	-5.92
26e	-3.49	9.57E-02	3.26E-04	-2.96	3.24E-01	1.10E-03	-5.22	1.78E-03	6.07E-06	-6.45
26f	-3.64	7.14E-02	2.29E-04	-3.06	2.70E-01	8.69E-04	-5.49	1.01E-03	3.25E-06	-6.49
35a	-4.31	1.41E-02	4.90E-05	-4.54	8.33E-03	2.90E-05	-7.25	1.63E-05	5.68E-08	-5.6
35b	-4.45	1.07E-02	3.52E-05	-4.64	6.97E-03	2.28E-05	-7.52	9.26E-06	3.03E-08	-5.63
35c	-4.46	1.30E-02	3.45E-05	-5.03	3.55E-03	9.42E-06	-7.58	1.00E-05	2.65E-08	-6.21
35d	-4.62	9.54E-03	2.41E-05	-5.13	2.93E-03	7.42E-06	-7.84	5.72E-06	1.45E-08	-6.25
35e	-3.2	1.84E-01	6.27E-04	-2.75	5.23E-01	1.78E-03	-4.84	4.22E-03	1.43E-05	-6.78
35f	-3.36	1.38E-01	4.41E-04	-2.85	4.37E-01	1.40E-03	-5.12	2.40E-03	7.67E-06	-6.82
